# New Carbamates and Ureas: Comparative Ability to Gel Organic Solvents

**DOI:** 10.3390/gels8070440

**Published:** 2022-07-14

**Authors:** Gabriela Martínez-Mejía, Brenda Afrodita Bermeo-Solórzano, Silvia González, José Manuel del Río, Mónica Corea, Rogelio Jiménez-Juárez

**Affiliations:** 1 Departamento de Química Orgánica, Escuela Nacional de Ciencias Biológicas, Instituto Politécnico Nacional, Prolongación de Carpio y Plan de Ayala S/N, Miguel Hidalgo, C.P., Ciudad de México 11340, Mexico; gmmejia@outlook.es (G.M.-M.); brenda_solorzanoo@hotmail.com (B.A.B.-S.); 2 Laboratorio de Investigación en Polímeros y Nanomateriales, Escuela Superior de Ingeniería Química e Industrias Extractivas, Instituto Politécnico Nacional, San Pedro Zacatenco, Alcaldía Gustavo A. Madero C.P., Ciudad de México 07738, Mexico; 3 Departamento de Química, Universidad Técnica Particular de Loja (UTPL), Loja 1101608, Ecuador; sylvyagp@gmail.com; 4 Departamento de Ingeniería en Metalurgia y Materiales, Escuela Superior de Ingeniería Química e Industrias Extractivas, Instituto Politécnico Nacional, San Pedro Zacatenco, Alcaldía Gustavo A. Madero C.P., Ciudad de México 07738, Mexico; jm.delrio.garcia@gmail.com

**Keywords:** amphiphilic compound, organogel, self-assembly, carbamates, ureas, organogelator, non-covalent interactions, gelling ability

## Abstract

Two series of novel amphiphilic compounds were synthesized based on carbamates and ureas structures, using a modification of the synthesis methods reported by bibliography. The compounds were tested for organic solvent removal in a model wastewater. The lipophilic group of all compounds was a hexadecyl chain, while the hydrophilic substituent was changed with the same modifications in both series. The structures were confirmed by FT-IR, NMR, molecular dynamic simulation and HR-MS and their ability to gel organic solvents were compared. The SEM images showed the ureas had a greater ability to gel organic solvents than the carbamates and formed robust supramolecular networks, with surfaces of highly interwoven fibrillar spheres. The carbamates produced corrugated and smooth surfaces. The determination of the minimum gelation concentration demonstrated that a smaller quantity of the ureas (compared to the carbamates, measured as the weight percentage) was required to gel each solvent. This advantage of the ureas was attributed to their additional N-H bond, which is the only structural difference between the two types of compounds, and their structures were corroborated by molecular dynamic simulation. The formation of weak gels was demonstrated by rheological characterization, and they demonstrated to be good candidates for the removal organic solvents.

## 1. Introduction

A great problem in the world is the water pollution by organic solvents in several industries. To solve this problem, purification and reuse processes of wastewater have been developed. A way to eliminate organic solvents of wastewater is adding amphiphilic compounds (low molecular weight organogelators) able to capture and remove them by filtration [1].

The design of new low molecular weight organogelators (LMWGs) has attracted a great attention due to their wide range of applications. They are currently used in organic waste recovery and consequently environmental sanitation [2,3] as vehicles for the transport and drugs delivery in medicine [4,5,6,7,8] as scaffolds in tissue engineering for regenerative medicine [9,10], preparation of nanoparticles for nanoreactors [11,12], as well as sensors of chemical bond breakage in hydrogels [13].

The chemical structures of gelators capable of forming organogels present groups as amides [14,15,16], carbamates [17,18,19,20], amino acids [21], carbohydrates [22,23,24,25,26,27], ureas [28,29,30,31,32,33] or a combination of these functional groups [34,35,36,37,38,39,40]. For this reason, all gelators of this study are composed of a hydrophilic and hydrophobic part, resulting in amphiphilic behavior [41,42]. These amphiphilic compounds can trap polar and non-polar solvents to form supramolecular networks by means of weak non-covalent interactions donor-acceptor as gelator-gelator, gelator-solvent and solvent-solvent intermolecular interactions [43,44,45,46,47,48,49,50,51], which are carried out by hydrogen bonding, dipole-dipole interactions, van der Waals molecular interactions, molecular stacking, π-π interactions, C-H… π interactions, solvophobic effects [52] or molecular surface stress. The literature indicates that a gelator is efficient if immobilizes to the solvent at concentrations ≤2 wt.% [53].

**Figure 1:**
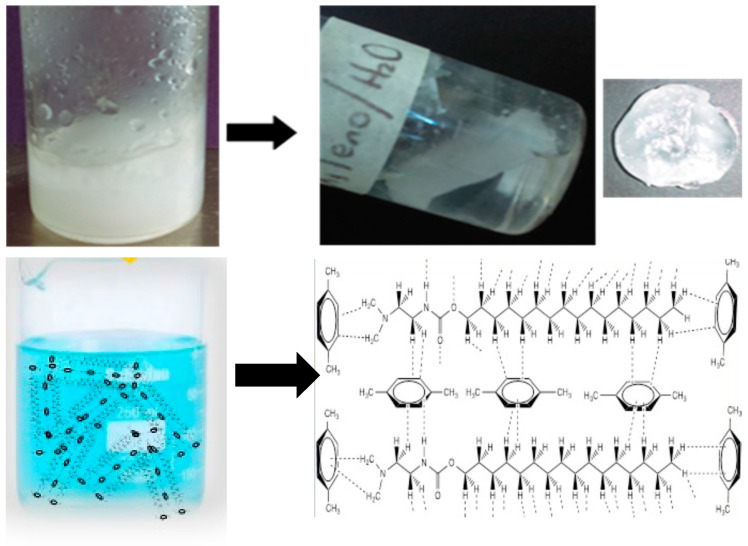
Scheme of formation of the gel and the subsequent removal of organic solvents.

A process of self-organization is known to be responsible for forming the supramolecular networks, which can pick up or arrest organic solvents. However, this process is still not clear. This phenomenon has been explored by few researchers and they have established some hypothesis [54,55,56]. For example, Lara Estroff and Andrew Hamilton [57,58] suggested that the organogels self-assembly process occurs in stages, beginning with the formation of a primary structure through molecular recognition, thus promoting anisotropic aggregates in one or two dimensions on a molecular scale ranging from Angstroms to nanometres. In the second stage, the morphology of the aggregates is more defined due to the formation of micelles, vectors, fibers, ribbons, leaves or other elements, depending on the molecular structure of the organogelator. The structure ranges from nanometers to micrometers. In the final stage, the tertiary structure of the gel is formed by the molecular interaction of the aggregates, with a scale ranging from microns to millimeters. Scheme gel formation and the removal of organic solvents is shown in Figure 1.

The aim of this study was to synthesize two series of novel organic compounds, which have not been reported in the literature: one of carbamates and the other of ureas, using a modification of synthesis methods reported by bibliography to increase the reaction efficiency. Five compounds were synthesized in each series, the hydrophobic part was the same (with a 16 carbons length of the hydrocarbon linear chain), while the hydrophilic substituent was changed and, the same modifications were made for the carbamates and ureas with the purpose to compare their ability to form supramolecular networks. The compounds were characterized by known methods and their supramolecular network’s ability was tested with four organic solvents (toluene, 1-4 dioxane, xylene and carbon tetrachloride). These solvents were chosen according to the latest WHO global report on contamination of organic solvent waste by the chemical industry sector reports them as persistent organic pollutants (POPs) [59].

Gel structures were examined by FT-IR and NMR, while their morphologies were observed by SEM. The stability of the organogels was evaluated by determining the breaking point, revealing that urea gels form the stronger supramolecular networks than the carbamate gels. This was attributed to the additional N-H bond in the urea compounds. The gel structures were also corroborated by molecular dynamic simulation. The removal efficiency of solvents in water was tested comparing the best carbamate with the best urea.

## 2. Results and Discussion

### 2.1. Synthesis of the New Carbamates and New Ureas

Two series of compounds were synthesized: five carbamates and five ureas which, according SciFinder they have never been reported except the **5b** structure, which has not been reported to be used as gelator. The hydrophobic part of all the compounds was the same (with 16 carbons hydrocarbon linear chain) whereas the hydrophilic part was changed. The used synthesis methods were modified from those reported by bibliography, allowing to obtain 85% and 90% of yield for carbamates and ureas, respectively (see Supplementary Scheme S1 and Supplementary Scheme S2).

The carbamate and urea structures were corroborated by HR-MS and the results of the values of the experimental and calculated molecular weights are close to each other, confirming the obtaining of desired product (Tables S1 and S2, Supplementary Materials).

### 2.2. Gelation Test of New Carbamates and New Ureas

The ability of carbamates and ureas to self-assemble and form gels tested in presence of organic solvents (xylene, toluene and 1,4-dioxane) using the inverting tube method. The gelator was evaluated by weight rather than volume. In this way, the required minimum gelation concentration (MGC) to form supramolecular networks was determined (see Figures S44–S47, Tables S3–S5 and S7, Supplementary Materials).

The results show that the ureas have a greater capacity than the carbamates to gel organic solvents, because it was always necessary to use a larger quantity of carbamates to form the gel. This was observed for example, by comparing **3d** carbamate and **5d** urea as gelators with the same organic solvent (toluene). The required quantity of carbamate for this case was 6.8 wt.%, while urea was only 1.1 wt.% (Tables S3 and S4, Supplementary Materials).

A way to confirm the gel formation was analyzing the pure compounds (Figures S1–S40, Supplementary Materials) and gelators by FT-IR. The spectra of carbamates pure show an absorption band close to 1691 cm^−1^ attributed to the carbonyl group, while for the urea compounds this band appears at around 1620 cm^−1^. This is attributed to the resonance effect of the oxygen and nitrogen atoms on the double bond of the carbonyl, being this phenomenon stronger for the urea than the carbamate. Consequently, the urea bond is longer, more polarized and weaker [60], meaning it is the carbonyl undergoes stretching with a lower frequency energy (Scheme S3, Supplementary Materials).

Comparing the FT-IR spectra for the best gelator compounds, the spectrum of **3a** carbamate organogel with xylene shows an intense and wide absorption band at 3350 cm^−1^ corresponds to the vibration of the NH stretch (Figures S42 and S43, Supplementary Materials). The spectrum of **5a** urea with carbon tetrachloride shows a broad and very weak band at 3347 cm^−1^ to the NH stretching vibration (Figures S45 and S46, Supplementary Materials). This means that the NH groups of the ureas are more involved in the formation of bonds by hydrogen bridges than the NH groups of the carbamates [61,62]. Another relevant characteristic was the decrement in the intensity of the absorption bands of the NH and carbonyl groups when the gels were formed. This effect is greater in ureas than carbamates and it is related to the formation of molecular networks.

The materials were also characterized by ^1^H NMR. The spectra of pure carbamates showed chemical shifts at δ close to 4.0 ppm for H_α_ protons, at about 3.3 ppm for H_β_ protons, except for **3e** whose H_α_ proton resounds at 4.28 ppm due it is adjacent to the aromatic system. Only carbamates **3a**, **3d** and **3e** showed H_β_ protons. On the other hand, **3b**, **3c** and **3e** compounds displayed additional signals owing to their aromatic protons (Table S1, Supplementary Materials).

On the other hand, the ^1^H NMR spectra of ureas presented chemical shifts at 3.3 ppm for the H_α_ protons and at 4.28 ppm for the H_β_ proton of the **5e** compound. The **5a**, **5d** and **5e** ureas presented signals for the H_β_ protons, while **5b**, **5c** and **5e** showed signals for aromatic protons (Tables S2 and S6, Supplementary Materials).

The structural identity of the carbamates and of the ureas corroborated by HR-MS, where their experimental and calculated molecular weights were very close to each other (Tables S1 and S2, Supplementary Materials).

**Figure 2:**
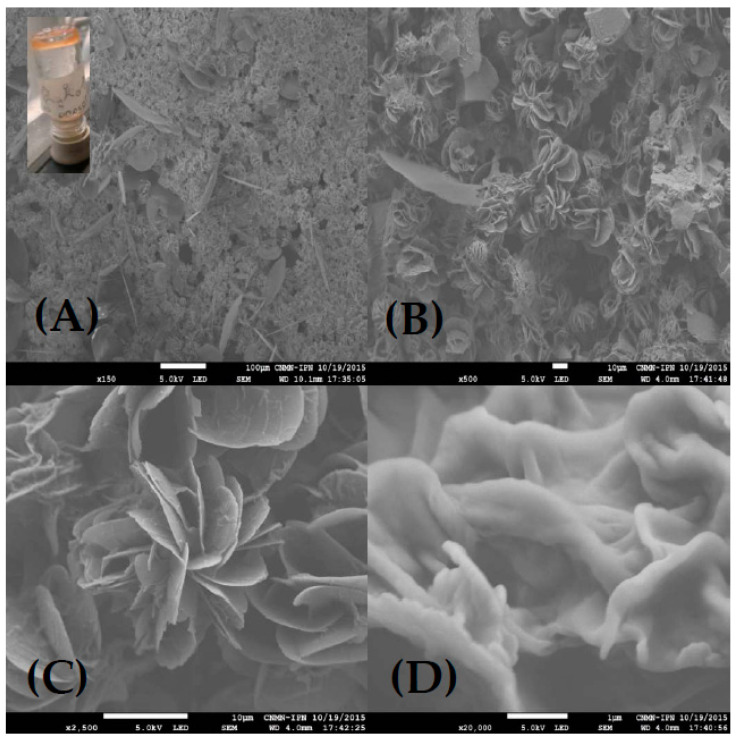
Micrographs of **3c** carbamate with 1,4-dioxane: (**A**) ×150, (**B**) × 500, (**C**) × 2500 and (**D**) × 20,000.

Gel morphology was analyzed by scanning electron microscopy. Results show clear differences between surfaces of the formed supramolecular networks by carbamates and ureas (see Figures S48 and S49, Supplementary Materials). Figure 2 shows SEM images of the supramolecular structures in the gels prepared from **3c** carbamate in dioxane. As can be seen, entanglement network structures of different type are formed. Specifically, Figure 2 shows gels an irregular entanglement network with average of 15 µm.

**Figure 3:**
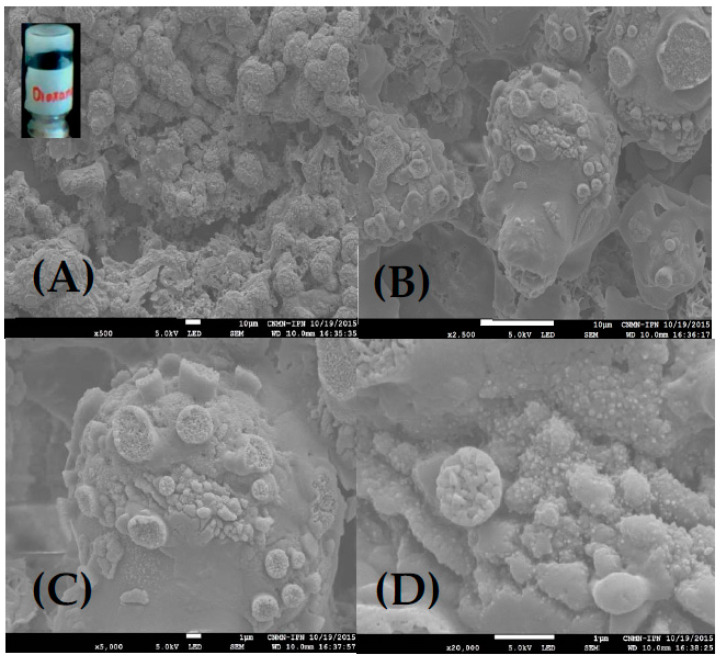
Micrographs of **5c** urea with 1,4-dioxane: (**A**) ×500, (**B**) × 2500, (**C**) × 5000 and (**D**) × 20,000.

SEM images of the supramolecular structures in the prepared from **5c** urea in dioxane are shown in Figure 3. Structures with irregular hollow shape and average sizes close to 10 µm exhibit a mixture of shapes.

The gel formation for ureas was also corroborated by Yang et al. in 2021, where structures of urea organogels were obtained as fibers and spheres, attribute to NH groups of compounds [63].

**Figure 4:**
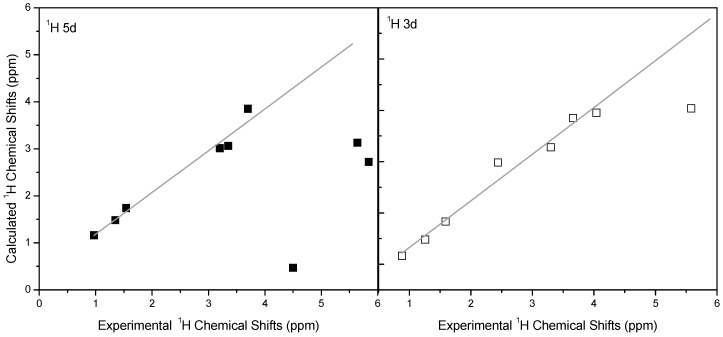
Linear correlation plots of 1H chemical shifts values of organogels with **5d** urea and **3d** carbamate.

To support the obtained NMR results of the organogels, the data of carbamates and ureas were processed by computational displacement calculations and the formed supramolecular interactions in the organogels were compared. Figure 4 presents a linear correlation plot of calculated and experimental ^1^H chemical shifts values of organogels to **5d** urea and **3d** carbamate as example. The results show that protons (^1^H) are significantly affected by intermolecular interactions, especially if they are compared with ^13^C chemical shifts [64]. These results could confirm the possible interactions of carbamates and ureas protons during the formation of the organogel (see Supplementary Figure S41). The linear correlation for all organogels is presented in Figures S50–S59 of Supplementary Materials.

The ^1^H chemical shifts between 4.5–6 ppm show the largest deviation to the **5d** urea and **3d** carbamate. The **5d** urea presents a signal at 4.3 ppm (NH) attributed to the formed hydrogen bonds between the organogel and the solvent, which cause a loss of correlation between calculated and experimental data. This has been reported by Baryshnikov [65]. For **3d** carbamate, this signal does not appear. For signals higher than 5 ppm attributed to the short chain attached to OH, the deviation could be to the electronegative property of oxygen atoms and a weak van der Waals interaction [66]. These findings can be supported by the structural analysis of **3d** carbamate and **5d** urea, which shows that the only difference between them is the additional NH bonding for the ureas. In this way, a greater amount of hydrogen bonds created to form supramolecular networks, either with the solvent or between the molecules of the compound itself, increasing its gelling efficiency [67].

The self-assembly ability of the best compounds (**3d** and **5d**) was also proved for the removal of organic solvents in water. Mixtures of toluene, xylene and 1,4-dioxane were tested by separated in water at concentrations of 20 wt.% of solvent, while mixtures of two and three organic solvents were prepared with water, where the concentration of each solvent was 20 wt.% (see Figures S69 and S70, Supplementary Material). The organic compound (carbamate or urea) was added following the same procedure described in gelation test section. The formed organogel was separated and weighed to obtain the removal efficiency of solvents. The gel was heated and the temperature at which it broke down was also recorded. The experiments were made by triplicate and the results are shown in Table 1.

In the same way, when the mixture was made with mixture of solvents in water, the **5d** urea had removal efficiencies higher than 70%, meanwhile the **3d** carbamate had removal efficiencies close to 60% except to dioxane where in both cases the compounds do not remove the solvent.

The efficiency results of the organic solvents removal were confirmed by FT-IR. The spectra of the initial mixture (water-solvent) and the final mixture (after capture of the solvent) were analyzed, see Figures S60–S67, Supplementary Materials. The band around 720 cm^−1^ was considered to differentiate between the used organic solvents. The area under the signal curve before and after the capture was calculated to obtain the experimental capture efficiency of solvents and they were compared with the gravimetric results (Table 2).

The rheological properties of the organogels were studied by oscillatory rheology at 25 °C. From the amplitude sweep experiments, storage modulus (G’) and loss modulus (G’’) were determined as a function of strain (see Figure S68, Supplementary Materials). The results show that G’ was higher than G’’ at low strain for all tested solvents systems in water. However, these modulus present higher values when the gel was made with **5d** urea than those made with **3d** carbamate, except to the mixture toluene-xylene-water, where both properties had the same values. Results show that the flow point is presented at low strains, typical behavior of weak gels [68,69]. However, these values are higher for gels made with urea than those made with carbamate.

**Figure 5:**
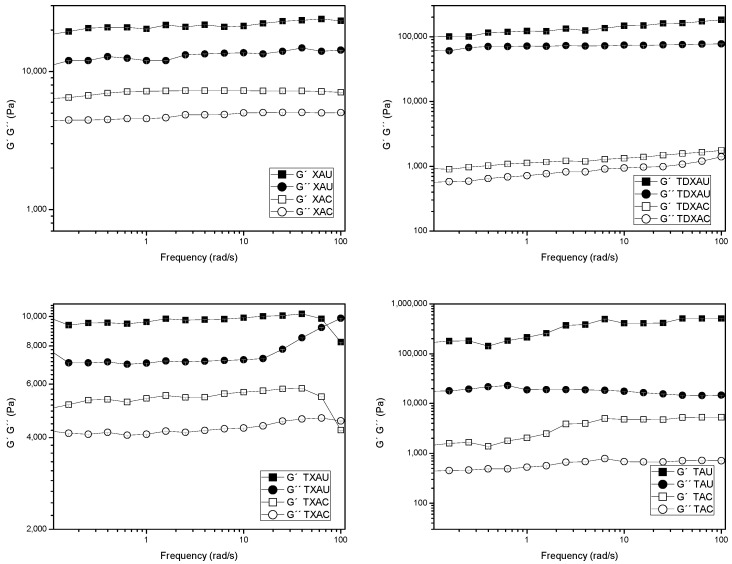
Frequency sweeps for organogels made with 5d urea (U) and **3d** carbamate (C), XA-xylene-water, TDXA-toluene-dioxane-xylene-water, TXA-toluene-xylene-water and TA- toluene-water.

It has reported to establish the gel structure, the storage modulus (G’) must dominate loss modulus (G’’) both contributions independent of frequency [70]. For this reason, G’ and G’’ for the gel were determined as a function of frequency (Figure 5) from 0.1 to 100 rad/s.

The results confirmed the elastic modulus was higher than viscose modulus at this range of frequency for all systems but, organogels **5d** urea presented higher values than those with **3d** carbamate. This is attributed to the formed hydrogen bonds are more important than van der Waals interactions. The materials were not affected by the frequency, which indicates that the organogels can support external forces in the range of used frequency, except to the system toluene-xylene-water. This behavior was reported by L.E. Ojeda-Serna [71], where oil-in-water organogels had an elasticity modulus higher than viscous, also attributed to van der Waals forces between alkyl chains that are related to aggregation to hydrophilic glycerol heads, which produces strong gels.

The results show that G’ and G’’ of organogels made of **5d** urea and toluene and are higher than those with xylene, indicating that formed organogels with toluene are more rigid because they have more physical interactions.

**Figure 6:**
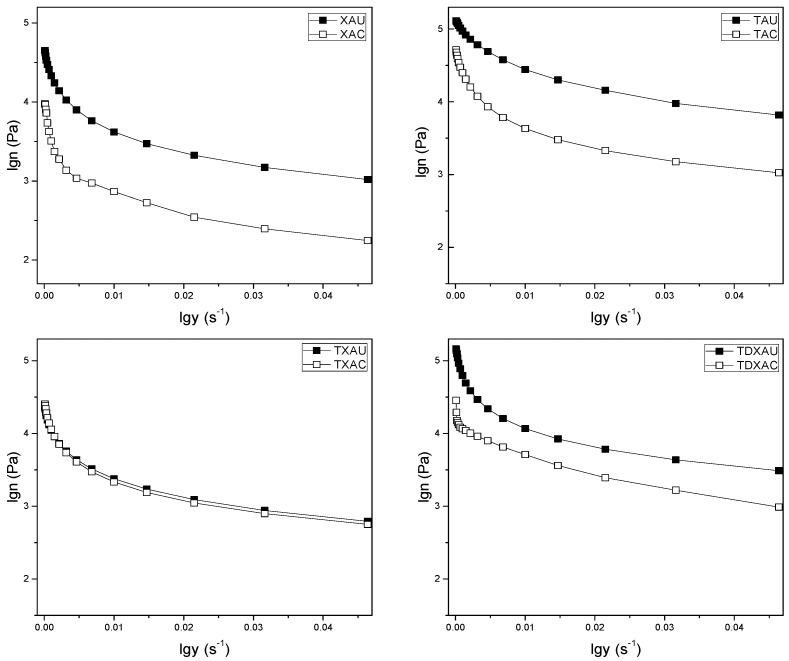
Apparent viscosity (η) as a function of shear rate (γ) for organogels of urea **5d** (■) and carbamate **3d** (☐) with mixture of solvents with water. Urea (U) and carbamate (C). Xylene-water (X-A), toluene- water (T-A), toluene-xylene-water (T-X-A) and toluene-dioxane-xylene-water (T-D-X-A).

Results of apparent viscosity (η) as a function of shear rate (y) for organogels made of **5d** urea and **3d** carbamate with mixture of solvents in water are presented in Figure 6. All the gel samples showed a prominent shear thinning flow behavior (*n* < 1) characteristic of a non-Newtonian behavior, but the degree of shear thinning was highest for urea organogels. This means that urea organogels have greater resistance to gradual deformations than carbamate organogels, because they have a greater interaction with all the solvents by the presence of more hydrogen bond in their structure that was demonstrated with the capture efficiency of solvents which was higher for ureas than the carbamates.

## 3. Conclusions

Two series of novel amphiphilic compounds (carbamates and ureas) were synthesized using a modification of synthesis methods reported by bibliography, which increased the reaction efficiency. The lipophilic part of both series was a hexadecyl chain and the hydrophilic substituent was changed, undergoing the same modifications in the carbamates and ureas. The structures were confirmed by several techniques and their ability to gel organic solvents was evaluated and compared. The ureas showed the greatest ability to self-assemble, which was attributed to the additional N-H bond. It was observed that hydrogen bonds worked cooperatively to stabilize and strengthen the supramolecular network. The results of NMR for gel formation were confirmed by computational displacement calculations, showing that the protons (^1^H) are significantly affected by intermolecular interactions. The results of one solvent removal in water showed that the urea had removal efficiencies above 80%, while with a mixture of solvents they had removal efficiencies higher than 70%. The rheological properties of gels showed typical behaviors of weak gels.

## 4. Materials and Methods

### 4.1. Materials

Hexadecyl chloroformate (96%), hexadecyl isocyanate (97%), N, N-dimethylethylenediamine (99%), ethanolamine (99%), 2-aminophenol (99%), 1,2-phenylenediamine (99%), palladium on carbon (10 wt%), hydroxylamine hydrochloride (99%), and vanillin (99%) were purchased from Sigma-Aldrich (Burlington, MA, USA) and used without further purification. Dichloromethane, ethyl acetate, hexane, acetone and ethyl alcohol were reagent grade from Alveg (Tlalnepantla de Baz, Mexico).

### 4.2. Methods

#### 4.2.1. Synthesis of New Carbamates and Ureas

**Scheme 1:**
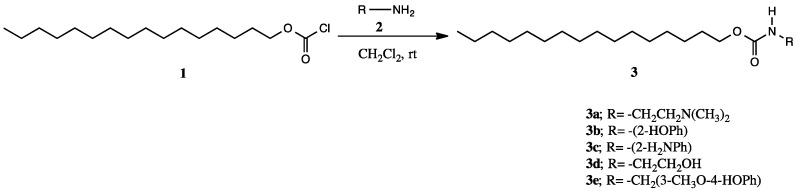
Synthesis of carbamates **3** starting from hexadecyl chloroformate **1** and amines **2**.

The general procedure for synthesizing the carbamates began by dissolving alkyl or aryl amines **2** (0.8 equiv.) in dichloromethane (DCM) in a round-bottomed flask under vigorous stirring. Then, hexadecyl chloroformate **1** (1 equiv.) in dichloromethane was added dropwise during 30 min at room temperature. The reaction progress was monitored by thin layer chromatography (TLC). After two hours, the reaction mixture was extracted with 5 *v*/*v*% aqueous hydrochloric acid solution (3 × 15 mL) and water (2 × 15 mL). The organic phase was dried (Na_2_SO_4_) and filtered and the solvent was evaporated under reduced pressure. The product was purified by recrystallization or by silica gel (70–230 mesh) column chromatography. The carbamates were obtained as a white or beige solid in a yield greater than 85% (see Supplementary Materials and Scheme 1).

**Scheme 2:**
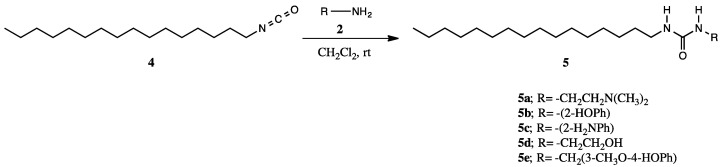
Synthesis of ureas **5** starting from hexadecyl isocyanate **4** and amines **2**.

The ureas were synthesized and purified following a procedure similar to carbamates synthesis. Hexadecyl isocyanate **4** and the alkyl or aryl amines **2** reacted to form ureas **5**. The ureas were obtained as a white solid with a yield greater than 90% (see Supplementary Materials and Scheme 2).

#### 4.2.2. Characterization of New Carbamates and Ureas

Melting points were determined by A.KRÜSS melting point meter (Model KSPIN, Berlin, Germany). Purification of the reaction mixtures was accomplished by recrystallization or column chromatography over silica gel (Merck 70-230 Boston, MA, USA) as a solid support. The reaction progress was monitored by TLC (thin layer chromatography) on silica gel 60 F254 aluminum plates (see Supplementary Materials). Fourier transform infrared spectroscopy (FT-IR) was recorded using a double-beam Perkin-Elmer Model 1605 FT/IR spectrometer (Whaltham, MA, USA) with ATR equipment. The area under the signal curve was calculated at 720 cm^−1^, before and after the capture of the solvents by an Origin LabPro (Northampon MA, USA).

NMR spectra were recorded in CDCl_3_ or DMSO-d6 solution on a Varian (now Agilent) NMR System 500 spectrometer (Agilent Technologies, Inc., Santa Clara, CA, USA), with 300 or 500 MHz for ^1^H NMR and 75 or 125 MHz for ^13^C, respectively. Chemical shifts were reported in parts per million (ppm) relative to Me4Si as internal standard. The J -coupling constants were expressed in Hz.

High-resolution mass spectroscopy (HR-MS) was analyzed on a micrOTOF-Q II with electrospray ionization (ESI) (BrukerDaltonics, Billerica, MA, USA).

#### 4.2.3. Gelation Test

The gelation properties were examined to each compound with four solvents. Briefly, a sample of 1 mL of solvent was put in a capped vial and weighed. The compound was added to solvent in quantities of 2 mg until saturation was reached. The mixture was heated in a thermal bath until the solid was dissolved and a clear solution was determined. The solution was cooled until gel formation and its temperature was registered. Finally, the vial was inverted to assure that there was not flow of organic solvent out of the gel [12]. The gel was weighed, and the minimum gelation concentration (MGC) was obtained. After that, the gel was heated and the temperature at which it broke down was recorded. All the experiments were performed by triplicate.

#### 4.2.4. Scanning Electron Microscopy

A gel sample was placed on a copper sample holder. The sample was submerged in liquid nitrogen for 10 min. With this treatment, the morphology of gels was maintained. Finally, the sample was put a vacuum condition and a layer of gold-palladium was sprayed on the sample [72,73]. The samples were placed in a JEOL scanning electron microscope (model JSM 7800F, Tokio, Japan) at 1 kV.

#### 4.2.5. Capture of Solvents in Wastewater Test

The organic solvent capture was examined for the best compounds: **3d** (carbamate) and **5d** (urea). Four samples of solvents-water were tested. The sample was prepared with 20 wt.% of solvent in water. Then, it was put in a capped vial and weighed. The compound addition (carbamate or urea) was made following the same procedure mentioned in the gelation test section. Finally, the formed organogel was separated and weighed. The gel was heated and the temperature at which it broke down was recorded. The experiments were made by triplicate.

#### 4.2.6. Rheological Analysis

Rheological properties were evaluated with a Modular Compact Rheometer model MCR-502, Anton Paar (Graz, Austria) using PP25 parallel plate geometry (25 mm diameter, 0°). Samples were placed in the center of the bottom plate. The upper plate was immediately lowered to a gap of 1 mm and the measurement was performed. Amplitude sweeps (strain = 0.001–100%) were carried out to determine the linear viscoelastic region (LVR). Oscillation stress sweep was conducted at 0.01% of strain and range of frequency of 0.1–100 rad/s was applied. In the frequency sweep, the angular frequency was varied from 10−1 to 102 rad/s at constant 0.01% strain. Apparent viscosity of samples at shear rate from 1 to 50 1/s was measured as a function of applied stress to check the structural loss of the gel. All experiments were made at 25 °C by triplicate.

#### 4.2.7. DFT Results of NMR Chemical Shift and IR Spectra

DFT calculations were performed using Gaussian 09 (Revision C.01) software (Wallington, CT, USA) and the results were visualized with GaussView 6.0 (Wallington, CT, USA). Structures were full optimized from the experimental data, using the Becke’s three-parameter (B3) hybrid exchange functional with the correlation functional of Lee Yang and Parr (LYP) [67,74]. The geometry optimizations for each species were calculated using 6-31G basis set with polarization functions in all atoms; IR frequencies were calculated using TZV basis sets of Ahlrichs and for calculate NMR chemical shifts the CC-pVDZ basis sets were employed. The reported NMR chemical shifts were calculated using GIAO-B3LYP method as implemented in Gaussian, all chemical shift values were corrected with scaling factors [67] for ^1^H and ^13^C, respectively. The optimization and computational IR spectra were calculated in vacuum and solvent effect, specifically chloroform was considered in NMR chemical shifts calculations.

## Figures and Tables

**Table 1:** Results of organic solvents removal (toluene, xylene and 1,4-dioxane) in water.

	Carbamate Compound				Urea Compound			
Mixture	Solvent (g)	Water (g)	3d (g)	Removal Efficiency (%)	Solvent (g)	Water (g)	5d (g)	Removal Efficiency (%)
D-W	0.2353	2.8036	0.0159	0.0	0.23	2.0783	0.0208	0.0
T-W	0.4884	2.3495	0.0201	28.00	0.4124	2.3160	0.1706	80.18
X-W	0.7671	3.2431	0.0252	82.53	0.7317	3.1273	0.0150	92.72
T-X-W	0.8819	1.2958	0.0258	58.95	0.0923	1.2584	0.0215	90.72
D-T-X-W	1.3522	1.0093	0.0256	62.40	1.3614	1.0147	0.0124	73.12

D = Dioxane; X = Xylene; T = Toluene; W= Water.

**Table 2:** Results of the evaluation of the traces of the organic solvents in water before and after the treatment with **3d** and **5d**.

	Carbamate Compound 3d		Urea Compound 5d	
Mixture	Gravimetry Removal Efficiency (%)	FT-IR Removal Efficiency (%)	Gravimetry Removal Efficiency (%)	FT-IR Removal Efficiency (%)
T-W	28.00	19.30	80.18	80.70
X-W	82.53	79.3	92.72	89.80
T-X-W	58.95	58.7	90.72	90.40
D-T-X-W	62.40	64.90	73.12	65.40

## Data Availability

Data are contained within the article or supplementary material.

## Supplementary Materials

The following supporting information can be downloaded at: https://www.mdpi.com/article/10.3390/gels8070440/s1, Experimental carbamate methods, Scheme S1. Synthesis of carbamates 3 starting from hexadecyl chloroformate 1 and amines 2; Figure S1. FT-IR of carbamate 3a; Figure S2. ^1^H NMR of carbamate 3a; Figure S3. ^13^C NMR of carbamate 3a; Figure S4. HR-MS of carbamate 3a; Figure S5. FT-IR of carbamate 3b; Figure S6. 1^H^ NMR of carbamate 3b; Figure S7. ^13^C NMR of carbamate 3b; Figure S8. HR-MS of carbamate 3b; Figure S9. FT-IR of carbamate 3c; Figure S10. 1^H^ NMR of carbamate 3c; Figure S11. ^13^C NMR of carbamate 3c; Figure S12. HR-MS of carbamate 3c; Figure S13. FT-IR of carbamate 3d; Figure S14. 1^H^ NMR of carbamate 3d; Figure S15. ^13^C NMR of carbamate 3d; Figure S16. HR-MS of carbamate 3d; Figure S17. FT-IR of carbamate 3e; Figure S18. 1^H^ NMR of carbamate 3e; Figure S19. ^13^C NMR of carbamate 3e; Figure S20. HR-MS of carbamate 3e; Scheme S2. Synthesis of ureas 5 starting from hexadecyl isocyanate 4 and amines 2; Figure S21. FT-IR of carbamate 5a; Figure S22. ^1^H NMR of urea 5a; Figure S23. ^13^C NMR of urea 5;a Figure S24. HR-MS of urea 5a; Figure S25. FT-IR of carbamate 5b; Figure S26. ^1^H NMR of urea 5b; Figure S27. ^13^C NMR of urea 5b, Figure S28. HR-MS of urea 5b; Figure S29. FT-IR of carbamate 5c; Figure S30. ^1^H NMR of urea 5c; Figure S31. ^13^C NMR of urea 5c; Figure S32. HR-MS of urea 5c; Figure S33. FT-IR of carbamate 5d; Figure S34. ^1^H NMR of urea 5d; Figure S35. ^13^C NMR of urea 5d; Figure S36. HR-MS of urea 5d; Figure S37. FT-IR of urea 5e; Figure S38. ^1^H NMR of urea 5e; Figure S39. ^13^C NMR of urea 5e; Figure S40. HR-MS of urea 5e; Table S1. Results of carbamates 3 in FT-IR, ^1^H NMR, and HR-MS; Table S2. Results of ureas 5 in FT-IR, ^1^H NMR, and HR-MS; Table S3. Comparative gelation properties of carbamates 3 (minimum gelation concentration, measured as the percentage of weight) with different organic solvents (CH_3_-(CH_2_)_14_-CH_2_-(OCONH)-R ^1^); Table S4. Comparative gelation properties of ureas 5 (minimum gelation concentration, measured as the percentage of weight) with different organic solvents (CH_3_-(CH_2_)_14_-CH_2_- (HNCONH)-R ^1^); Table S5. Gel formation temperatures and breaking temperatures (Tg)/(Tb) for carbamates 3a-3e and ureas 5a-5e; Table S6. Chemical shifts (δ ppm) of the N-H bonds of carbamates 3a-3e and ureas 5a5e; Table S7. Solubility parameter, molar volume and the Flory-Huggins parameter for several solvents; Figure S41. The only difference between the carbamates and the ureas is an additional NH bond in the latter 3e/5e; Figure S42. FT-IR spectrum of neat carbamate 3a; Figure S43. FT-IR spectrum of the gel formed by carbamate 3a with xylene; Figure S44. Supramolecular network formed by carbamate 3a with xylene; Figure S45. FT-IR spectrum of neat urea 5a; Figure S46. FT-IR spectra of the gel formed by urea 5a with carbon tetrachloride; Figure S47. Supramolecular networks formed by urea 5a with carbon tetrachloride; Figure S48. Photographs of the flasks and scanning electron microscopy micrographs of the gels obtained by interacting carbamates 3a-3e with different organic solvents; Figure S49. Photographs of the flasks and scanning electron microscopy micrographs of the gels obtained by interacting ureas 5a-5e with different organic solvents; Scheme S3. Resonant effect on the nitrogen and oxygen atoms on the double bond of the carbonyl; Figure S50. Linear correlation plots of a ^13^C and ^1^H chemical shifts values of organogel with urea 5a; Figure S51. Linear correlation plots of a ^13^C and ^1^H chemical shifts values of organogel with urea 5b; Figure S52. Linear correlation plots of a ^13^C and ^1^H chemical shifts values of organogel with urea 5c; Figure S53. Linear correlation plots of a ^13^C and ^1^H chemical shifts values of organogel with urea 5d; Figure S54. Linear correlation plots of a ^13^C and ^1^H chemical shifts values of organogel with urea 5e; Figure S55. Linear correlation plots of a ^13^C and ^1^H chemical shifts values of organogel with carbamate 3a; Figure S56. Linear correlation plots of a ^13^C and ^1^H chemical shifts values of organogel with carbamate 3b; Figure S57. Linear correlation plots of a ^13^C and ^1^H chemical shifts values of organogel with carbamate 3c; Figure S58. Linear correlation plots of a ^13^C and ^1^H chemical shifts values of organogel with carbamate 3d; Figure S59. Linear correlation plots of a ^13^C and ^1^H chemical shifts values of organogel with carbamate 3e; Figure S60. FT-IR spectrum of the traces of toluene in water before and after the treatment with 3d; Figure S61. FT-IR spectrum of the traces of toluene in water before and after the treatment with 5d; Figure S62. FT-IR spectrum of the traces of xylene in water before and after the treatment with 3d; Figure S63. FT-IR spectrum of the traces of xylene in water before and after the treatment with 5d; Figure S64. FT-IR spectrum of the traces of toluene- xylene in water before and after the treatment with 3d; Figure S65. FT-IR spectrum of the traces of toluene- xylene in water before and after the treatment with 5d; Figure S66. FT-IR spectrum of the traces of dioxane-toluene- xylene in water before and after the treatment with 3d; Figure S67. FT-IR spectrum of the traces of dioxane-toluene- xylene in water before and after the treatment with 5d; Figure S68. Storage modulus (G’) and loss modulus (G’’) as a function of strain for organogels of urea 5d (G’ -■)) (G’’-●) and carbamate 3d (G’-♦) (G’’-ϒ) with mixture of solvents with water. Urea (U) and carbamate (C). Xylene-water (X-A), toluene- water (TA), toluene-xylene-water (T-X-A) and toluene-dioxane-xylene-water (T-D-X-A); Figure S69. Photographs of removal organogels solvent of carbamate 3d with mixture of solvents with water. (A). Dioxane-water, (B) toluene-water, (C) xylene-water, (D) toluene-xylene-water and (E) dioxane-toluene-xylene-water; Figure S70. Photographs of removal organogels solvent of urea 5d with mixture of solvents with water. (A). Dioxane-water, (B) toluene-water, (C) xylene-water, (D) toluene-xylene-water and (E) dioxane-toluene-xylene-water.
