# Supplementary material for: New Carbamates and Ureas: Comparative Ability to Gel Organic Solvents
Source: Gels. 2022 Jul 14;8(7):440. doi: 10.3390/gels8070440 (PMC9316452; doi:10.3390/gels8070440)
Supplement: Supplementary file 1 [file gels-08-00440-s001.zip › gels-1753386-supplementary.pdf]

# New Carbamates and ureas: Comparative ability to gel organic solvents

Gabriela Martínez-Mejía[1,2], Brenda Afrodita Bermeo-Solórzano[1], Silvia González[2], José Manuel del Río[3], Mónica Corea[4†], Rogelio Jiménez-Juárez[1*]

[1] Departamento de Química Orgánica, Escuela Nacional de Ciencias Biológicas, Instituto Politécnico Nacional, Prolongación de Carpio y Plan de Ayala S/N, Miguel Hidalgo, C.P. 11340, Ciudad de México, México.

[2] Departamento de Química, Universidad Técnica Particular de Loja (UTPL), Loja 1101608, Ecuador.

[3] Departamento de Ingeniería en Metalurgia y Materiales, Escuela Superior de Ingeniería Química e Industrias Extractivas, Instituto Politécnico Nacional, San Pedro Zacatenco, Alcandía Gustavo A. Madero C.P. 07738, Ciudad de México, México.

[4] Laboratorio de Investigación en Polímeros y Nanomateriales. Escuela Superior de Ingeniería Química e Industrias Extractivas, Instituto Politécnico Nacional, San Pedro Zacatenco, Alcandía Gustavo A. Madero C.P. 07738, Ciudad de México, México.

# Electronic Supplementary Information (ESI)

TABLE OF CONTENTS


Experimental carbamate methods

Supplementary Scheme S1

Supplementary Figure S1

Supplementary Figure S2

Supplementary Figure S3

Supplementary Figure S4

Supplementary Figure S5

Supplementary Figure S6

Supplementary Figure S7

Supplementary Figure S8

Supplementary Figure S9

Supplementary Figure S10

Supplementary Figure S11

Supplementary Figure S12

Supplementary Figure S13

Supplementary Figure S14

Supplementary Figure S15

Supplementary Figure S16

Supplementary Figure S17

Supplementary Figure S18

Supplementary Figure S19

Supplementary Figure S20

Experimental urea methods

Supplementary Scheme S2

Supplementary Figure S21

Supplementary Figure S22

Supplementary Figure S23

Supplementary Figure S24

Supplementary Figure S25

Supplementary Figure S26

Supplementary Figure S27

Supplementary Figure S28

Supplementary Figure S29

Supplementary Figure S30

Supplementary Figure S31

Supplementary Figure S32

Supplementary Figure S33

Supplementary Figure S34

Supplementary Figure S35

Supplementary Figure S36

Supplementary Figure S37
Supplementary Figure S38
Supplementary Figure S39
Supplementary Figure S40
Supplementary Table S1
Supplementary Table S2
Supplementary Table S3
Supplementary Table S4
Supplementary Table S5
Supplementary Table S6
Supplementary Table S7
Supplementary Figure S41
Supplementary Figure S42
Supplementary Figure S43
Supplementary Figure S44
Supplementary Figure S45
Supplementary Figure S46
Supplementary Figure S47
Supplementary Figure S48
Supplementary Figure S49
Supplementary Figure S50
Supplementary Figure S51
Supplementary Figure S52
Supplementary Figure S53
Supplementary Figure S54
Supplementary Figure S55
Supplementary Figure S56
Supplementary Figure S57
Supplementary Figure S58
Supplementary Figure S59
Supplementary Figure S60
Supplementary Figure S61
Supplementary Figure S62
Supplementary Figure S63
Supplementary Figure S64
Supplementary Figure S65
Supplementary Figure S66
Supplementary Figure S67
Supplementary Figure S68
Supplementary Figure S69
Supplementary Figure S70

## 1. EXPERIMENTAL METHODS

*Materials*

Hexadecyl chloroformate (96%), hexadecyl isocyanate (97%), N,N-dimethylethylenediamine (99%), ethanolamine (99%), 2-aminophenol (99%), 1,2-phenylenediamine (99%), palladium on carbon (10wt%), hydroxylamine hydrochloride (99%), and vanillin (99%) were purchased from Sigma-Aldrich (USA) and used without further purification. Dichloromethane, ethyl acetate, hexane, acetone, ethyl alcohol were reagent grade, purchased from Alveg (México). Melting points were determined by a KRUSS melting point meter (Model KSPIN, Germany). Fourier transform infrared spectroscopy (FT-IR) was recorded using a double-beam Perkin-Elmer Model 1605 FT/IR spectrometer (USA) with ATR equipment. NMR spectra were recorded on a Varian Mercury spectrometer (Agilent Technologies, Inc., Santa Clara, CA, USA), (300 or 500 MHz for $^{1}H$ NMR, 75 or 125 MHz for $^{13}C$ respectively). Chemical shifts are reported in parts per million relative to $Me_4Si$ as internal standard. Coupling constants *J* are expressed in Hz. High resolution mass spectroscopy (HR-MS) was analyzed on a micrOTOF-Q II with electrospray ionization (ISI) (BrukerDaltonics, Billerica USA). All measurements were carried out by triplicate at room temperature. Purification of the reaction mixtures was carried out by column chromatography using silica gel (Merck 70-230 Mesh) as a solid support or by recrystallization. The progress of the reaction was followed by thin layer chromatography (TLC) on silica gel 60 $F_{254}$ Aluminium plates.

*General Procedures*

Synthesis of the new carbamates

The general procedure for synthesizing the carbamates began by dissolving alkyl or aryl amines **2** (0.8 equiv.) in dichloromethane (DCM) in a round-bottomed flask under vigorous stirring. Then, hexadecyl chloroformate **1** (1 equiv.) in dichloromethane was added dropwise during 30 min at room temperature. The reaction progress was monitored by thin layer chromatography (TLC). After two hours, the reaction mixture was extracted with 5v/v% aqueous hydrochloric acid solution (3x15 mL) and water (2x15 mL). The organic phase was dried ($Na_2SO_4$) and filtered and the solvent was evaporated under reduced pressure. The reaction crude product was purified by recrystallization or by silica gel (70-100 mesh) column chromatography. The carbamates were obtained as a white or beige solid in a yield greater than 85% (Scheme 1).

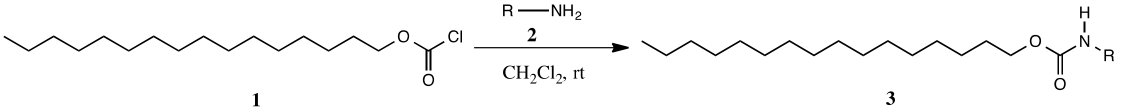


**1** **3**

**3a**; R= $-CH_2CH_2N(CH_3)_2$
**3b**; R= -(2-HOPh)
**3c**; R= -(2-$H_2$NPh)
**3d**; R= $-CH_2CH_2OH$
**3e**; R= $-CH_2$(3-$CH_3$O-4-HOPh)

Scheme S1. Synthesis of carbamates **3** starting from hexadecyl chloroformate **1** and amines **2**.

*O*-Hexadecyl-N,*N-Dimethylethylenediaminecarbamate (**3a**)*

The carbamate **3a** was obtained as white solid, 564 mg (94 %) yield; Table 1, M.p.: 79-80 °C; IR (KBr): ν 3311 (NH), 2917, 2852 (H-CH-), 1685 (O=C-NH) $cm^{-1}$; $^1$H NMR (500 MHz, $Cl_3CD$): δ = 0.88 (t, 3H, *J*=5.0 Hz, $CH_3R$), 1.25 (br, 26H, $(CH_2)_{13}$), 1.60 (br, 2H, $RCH_2C$), 2.92 (s, 6H, $(CH_3)_2N$), 3.28 (br, 2H, $CH_2N(CH_3)_2$), 3.65 (br, 2H, $NHCH_2$), 4.04

(br, 2H, $CH_2O$), 6.61 (br, 1H, NH) ppm; $^{13}C$ NMR (125 MHz, $Cl_3CD$): δ = 157.0 (OCON), 65.3, 57.8, 43.6, 36.1, 31.8, 29.5, 29.2, 28.8, 22.5, 13.9 ppm; HR-MS (m/z) experimental molecular weight (M+1) 357.3486 g/mol. Calculated molecular weight 357.3481 g/mol.

*O-Hexadecyl-N-(2-hydroxyphenyl) carbamate (**3b**)*

The **3b** carbamate was obtained as white solid, 574 mg (83 %) yield; Table 1, M.p.: 77-78 °C; IR (KBr): ν 3296 (NH), 2916 y 2850 (H-CH-), 1681 (O=C-NH) $cm^{-1}$; $^{1}H$ NMR (500 MHz, $Cl_3CD$): δ = 0.88 (t, 3H, *J*=7 Hz, $CH_3R$), 1.34 (br, 26H, $(CH_2)_{13}$), 1.68 (q, 2H, *J*=7 Hz, $RCH_2C$), 4.18 (t, 2H, *J*=7 Hz, $RCH_2O$), 6.79 (br, 1H, NH), 6.89 (t, 1H, *J*=7 Hz, $H_4Ar$), 6.97 (d, 1H, *J*=7 Hz, $H_5Ar$), 7.04 (t, 1H, *J*=7 Hz, $H_3Ar$), 7.19 (d, 1H, *J*=7 Hz, $H_6Ar$), 7.69 (br, 1H, OH) ppm; $^{13}C$ NMR (125 MHz, $Cl_3CD$): δ = 158.5 (OCON), 155.5, 147.2, 125.6, 125.2, 121.3, 120.8, 66.5, 31.8-20.0, 25.7, 22.6, 18.9, 13.9 ppm; HR-MS (m/z) experimental molecular weight (Sodium salt) 400.2822 g/mol. Calculated molecular weight 400.2827 g/mol.

*O-Hexadecyl-N-(2-aminophenyl) carbamate (**3c**)*

The **3c** carbamate was obtained as white solid, 582 mg (97 %) yield; Table 1, M.p.: 84-85 °C; IR (KBr): ν 3296 (NH), 2916, 2850 (H-CH-), 1681 (O=C-NH) $cm^{-1}$; $^{1}H$ NMR (500 MHz, $Cl_3CD$): δ = 0.88 (t, 3H, *J*=7 Hz, $CH_3R$), 1.34 (br, 26H, $(CH_2)_{13}$), 1.65 (q, 2H, *J*=7 Hz, $RCH_2C$), 3.73 (br, 2H, $NH_2$), 4.14 (m, 2H, $RCH_2O$), 6.32 (br, 1H, NH), 6.78 (c, 1H, *J*=7.6 Hz, $H_4Ar$), 7.02 (t, 1H, *J*=7.6 Hz, $H_5Ar$), 7.14 (t, 1H, *J*=3.5 Hz, $H_3Ar$), 7.15 (d, 1H, *J*=3.5 Hz, $H_6Ar$) ppm; $^{13}C$ NMR (125 MHz, $Cl_3CD$): δ = 154.3 (OCON), 154.0, 139.8, 124.9, 123.5, 117.3, 115.9, 64.5, 39.8-28.7, 28.5, 25.1, 21.8, 13.1 ppm; HR-MS (m/z)

experimental molecular weight (Sodium salt) 399.2982 g/mol. Calculated molecular weight 399.2987 g/mol.

*O-Hexadecyl-N-(2-hydroxyethyl) carbamate **(3d)***

The **3d** carbamate was obtained as white solid, 503 mg (90 %) yield; Table 1, M.p.: 73-74 °C; IR (ATR): ν 3309 (NH), 2917, 2851 (H-CH-), 1691, 1549 (O=C-NH) $cm^{-1}$; $^{1}H$ NMR (500 MHz, $Cl_3CD$): δ = 0.88 (t, 3H, *J*=5 Hz, $CH_3R$), 1.26 (br, 26H, $(CH_2)_{13}$), 1.59 (br, 2H, $RCH_2C$), 2.44 (br, 1H, OH), 3.30 (br, 2H, $NHCH_2$), 3.66 (br, 2H, $CH_2OH$), 4.04 (t, 2H, *J*=5 Hz, $CH_2O$), 5.58 (br, 1H, NH) ppm; $^{13}C$ NMR (125 MHz, $Cl_3CD$): δ = 156.9 (OCON), 64.7, 61.5, 43.3, 31.6, 29.4-29.0, 25.6, 22.4, 13.8 ppm; HR-MS (m/z) experimental molecular weight (Sodium salt) 352.2842 g/mol. Calculated molecular weight 352.2827 g/mol.

*O-Hexadecyl-N-(4-hydroxy-3-methoxyphenyl)methylcarbamate **(3e)***

The **3e** carbamate was obtained as white solid, 329 mg (55 %) yield; Table 1; M.p.: 79-80 °C; IR (ATR): ν 3505, 3313 (NH, OH), 2917, 2847 (H-CH-), 1683, 1534 (O=CNH) $cm^{-1}$; $^{1}H$ NMR (500 MHz, $Cl_3CD$-DMSO): δ = 0.88 (t, 3H, *J*=5 Hz, $CH_3R$), 1.25 (br, 26H, $(CH_2)_{13}$), 1.61 (m, 2H, $RCH_2C$), 3.88 (s, 3H, $CH_3O$), 4.08 (t, 2H, *J*=5 Hz, $RCH_2OCO$), 4.28 (d, 2H, *J*=5 Hz, $NCONCH_2Ar$), 4.87 (br, 1H, NH), 5.60 (s, 1H, OH), 6.78 (d, 1H, *J*=9 Hz, $H_6Ar$), 6.81 (br, 1H, $H_2Ar$), 6.87 (d, 1H, *J*=9 Hz, $H_5Ar$) ppm; $^{13}C$ NMR (125 MHz, $Cl_3CD$): δ = 156.6 (O-CN=O, 149.6, 145.0, 130.5, 120.4, 114.3, 110.3, 65.2, 55.9, 45.0, 31.9, 29.6-29.0, 25.8, 22.7, 14.1 ppm; HR-MS (m/z) experimental molecular weight (Sodium salt) 421.3433 g/mol. Calculated molecular weight 421.3192 g/mol.

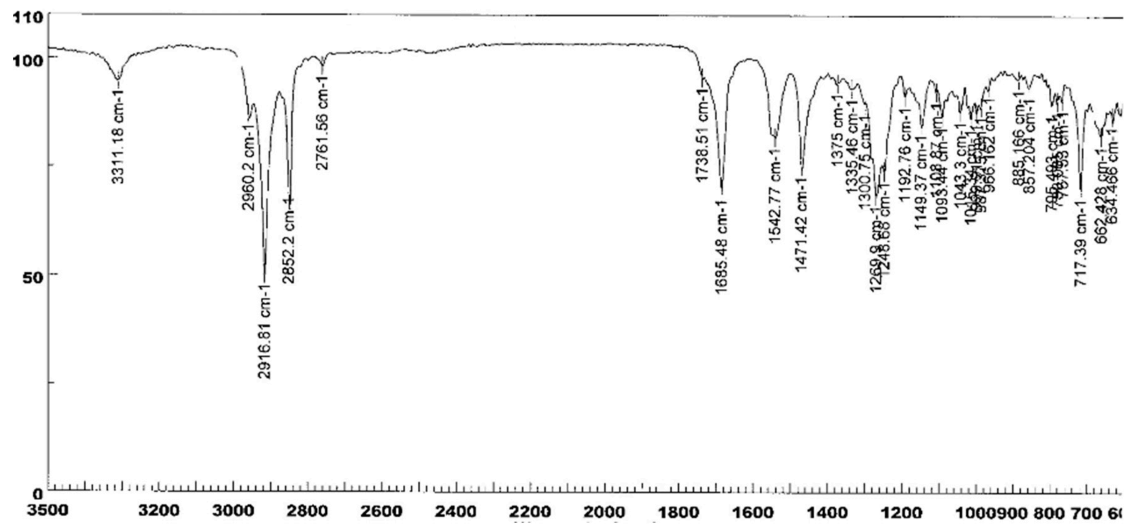

Figure S1. FT-IR of **3a** carbamate

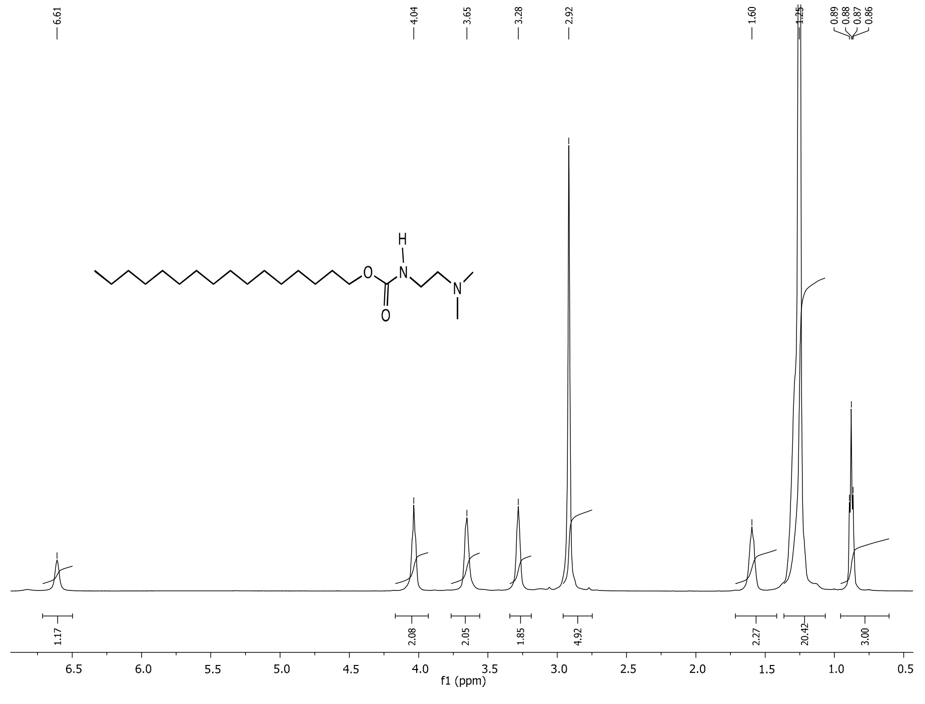

Figure S2. $^{1}$H NMR of **3a** carbamate

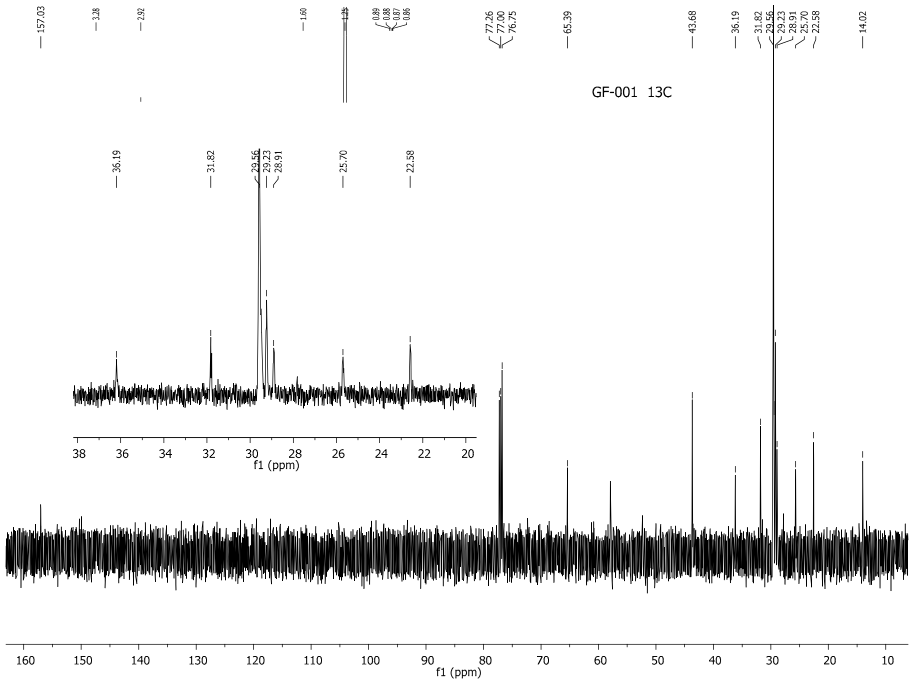


Figure S3. $^{13}$C NMR of **3a** carbamate

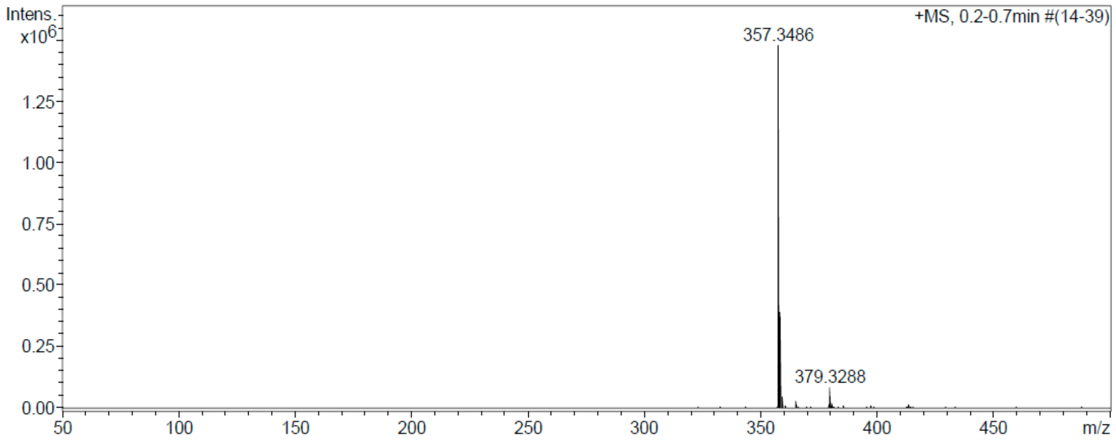


Figure S4. HR-MS of **3a** carbamate

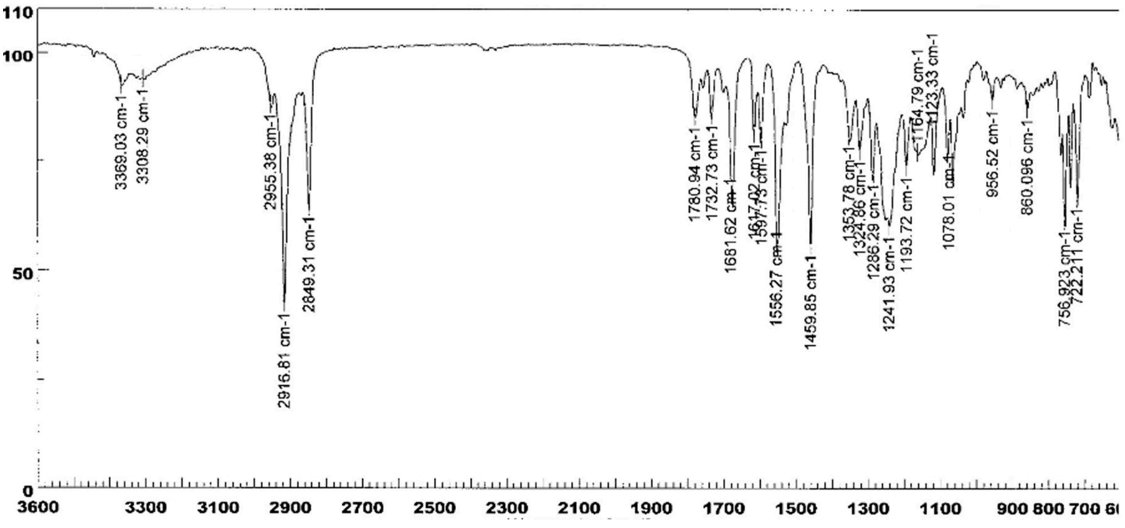


Figure S5. FT-IR of **3b** carbamate

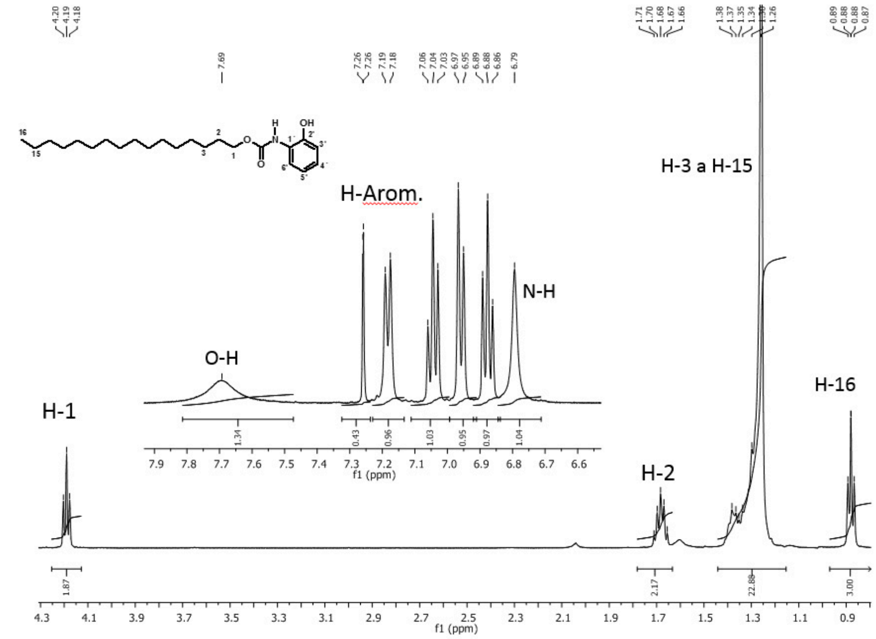


Figure S6. $^{1}$H NMR of **3b** carbamate

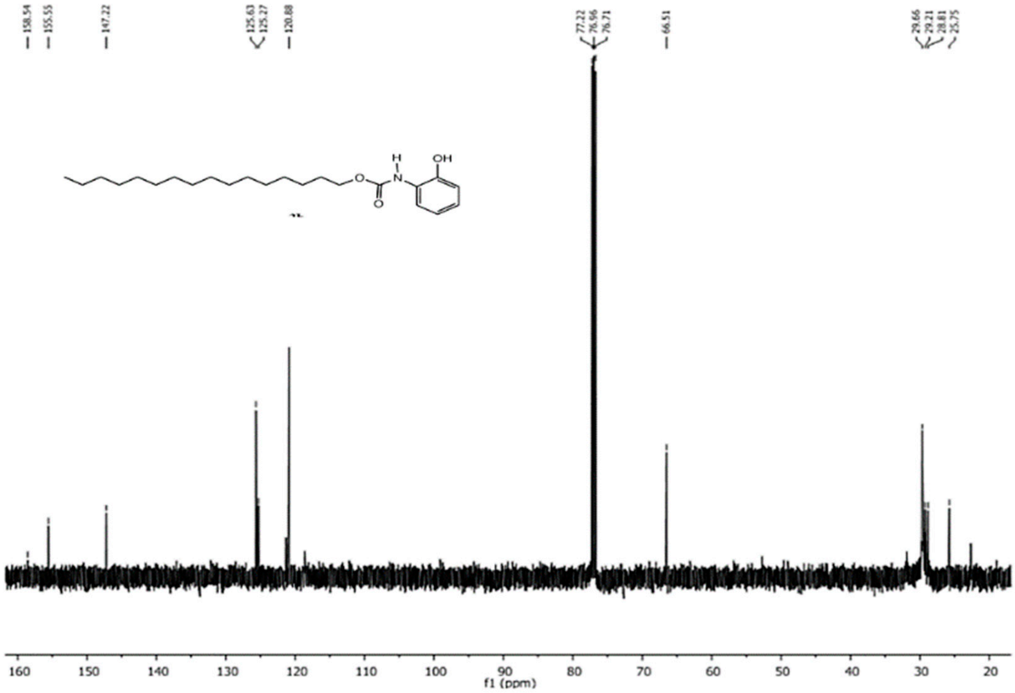


Figure S7. $^{13}$C NMR of **3b** carbamate

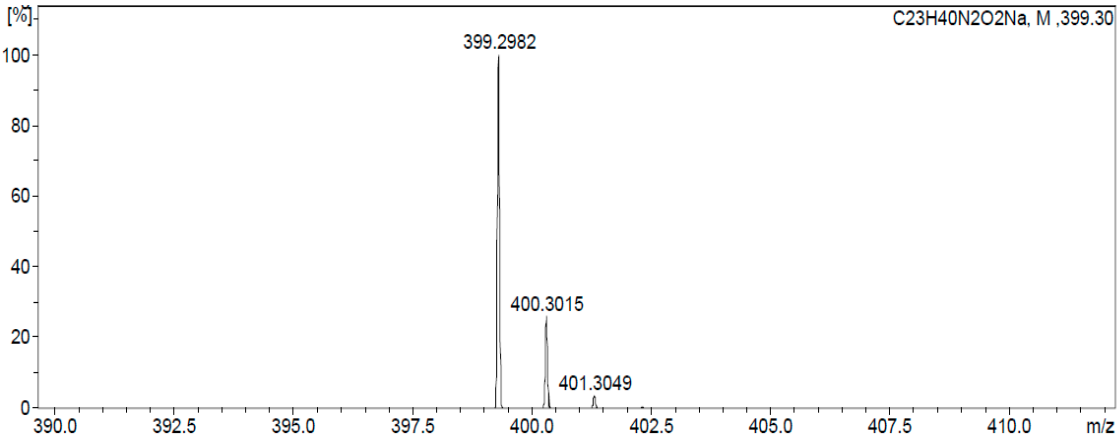


Figure S8. HR-MS of **3b** carbamate

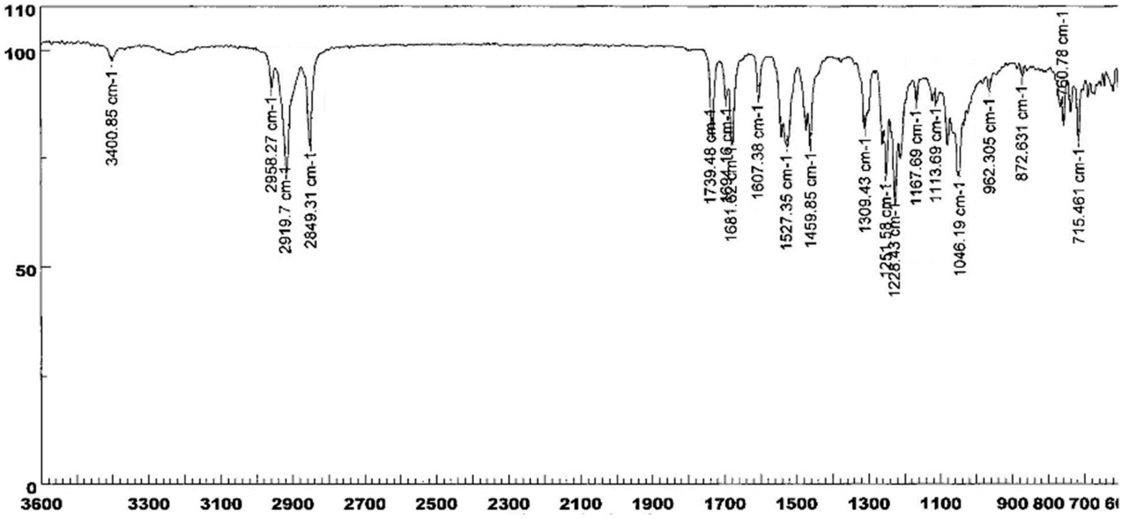


Figure S9. FT-IR of **3c** carbamate

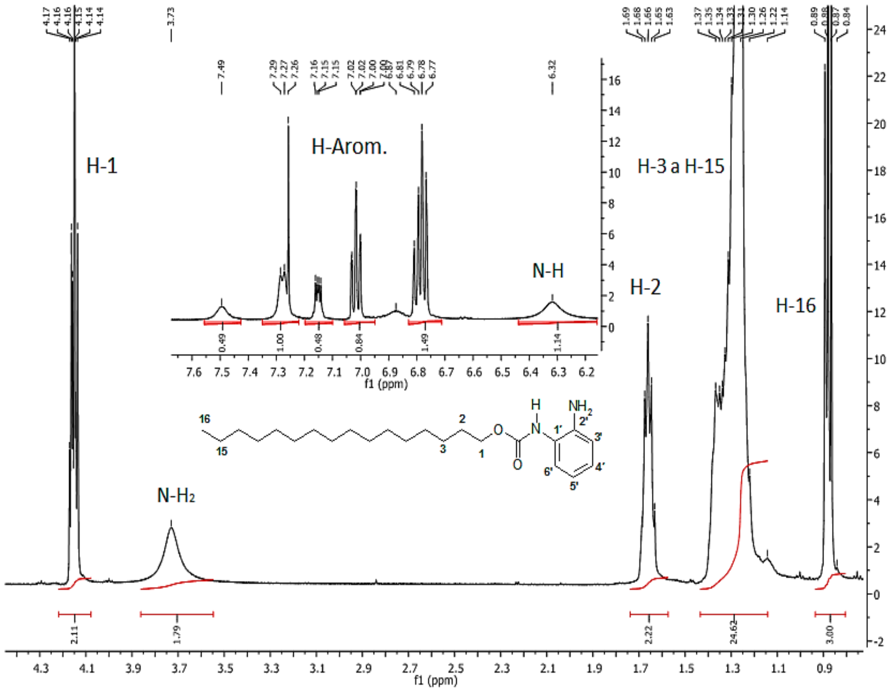


Figure S10. $^{1}$H NMR of **3c** carbamate

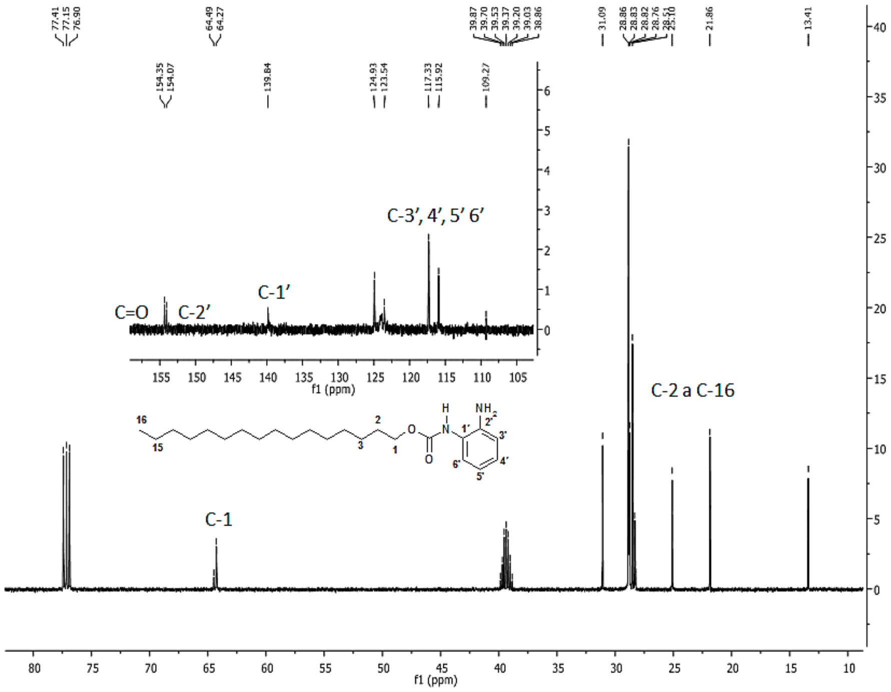


Figure S11. $^{13}$C NMR of **3c** carbamate

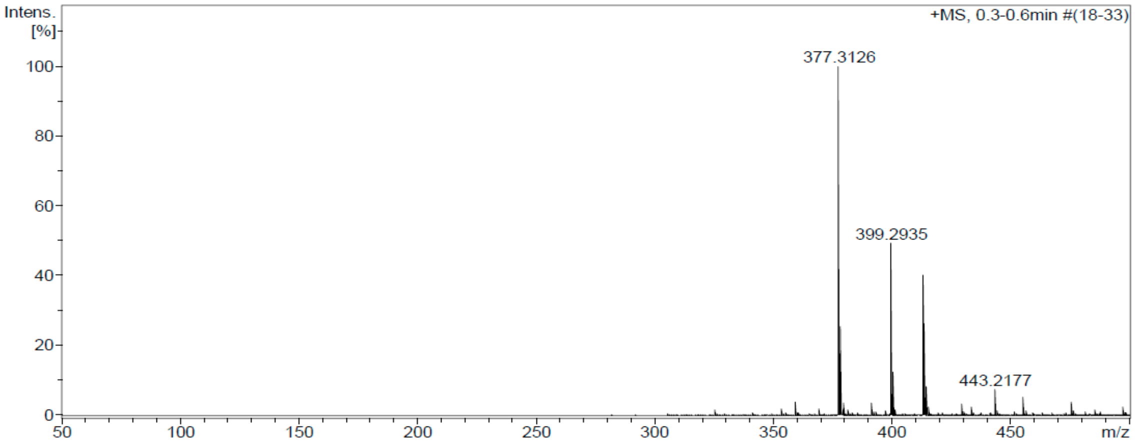


Figure S12. HR-MS of **3c** carbamate

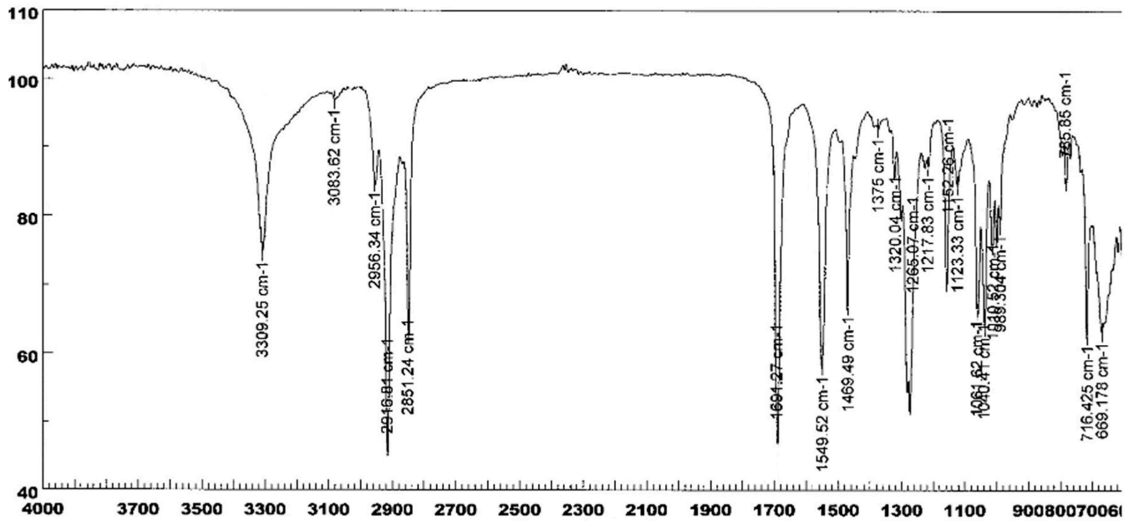


Figure S13. FT-IR of **3d** carbamate

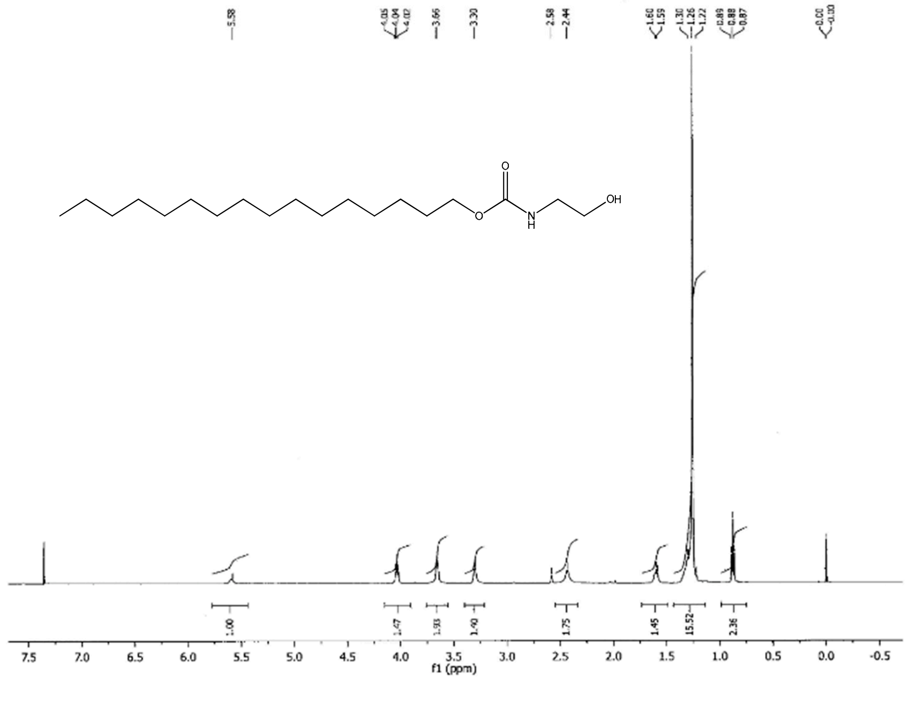


Figure S14. $^{1}$H NMR of **3d** carbamate

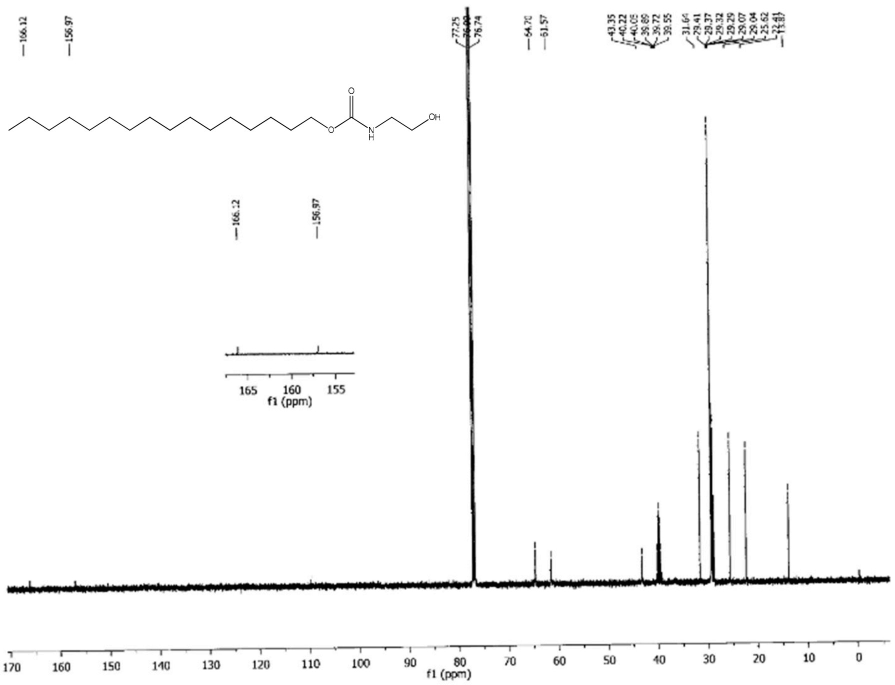

Figure S15. $^{13}$C NMR of **3d** carbamate

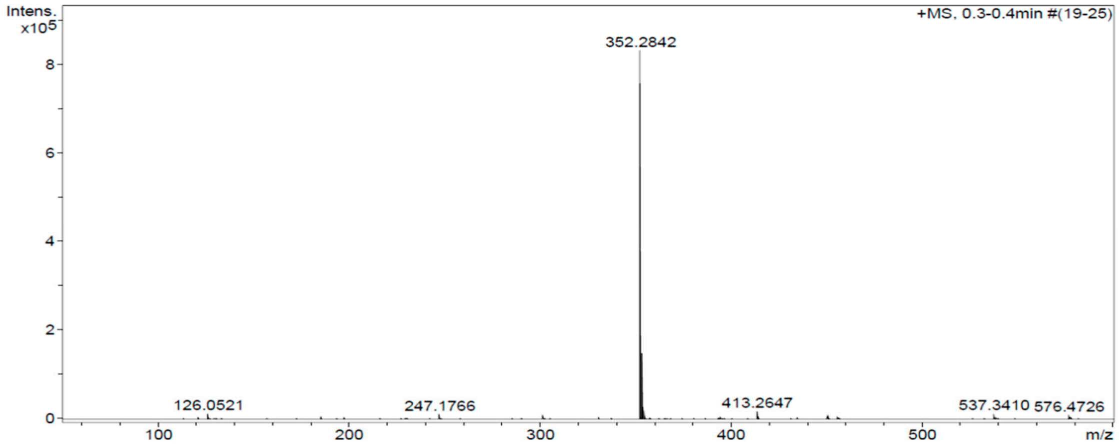


Figure S16. HR-MS of **3d** carbamate

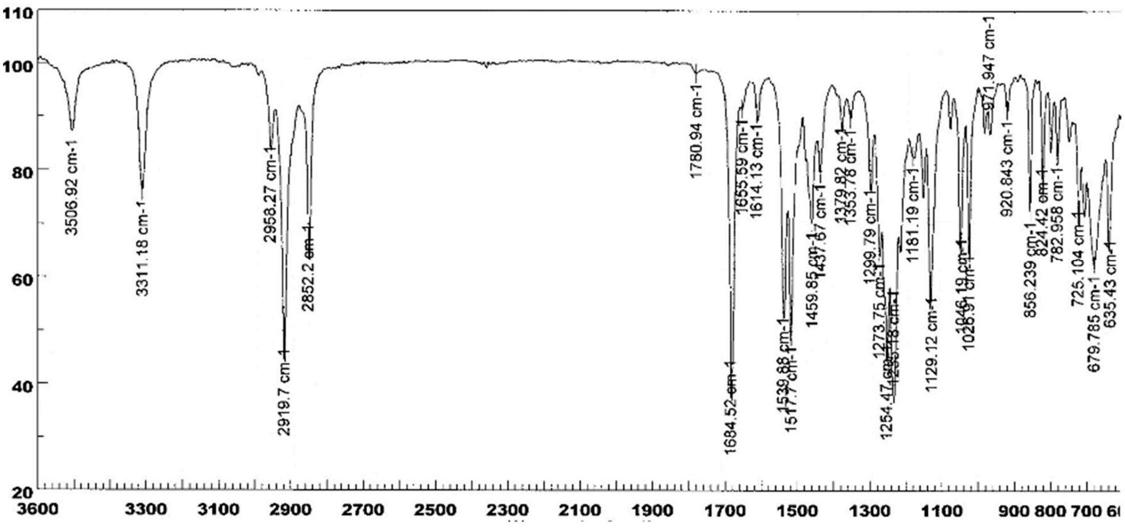

Figure S17. FT-IR of **3e** carbamate

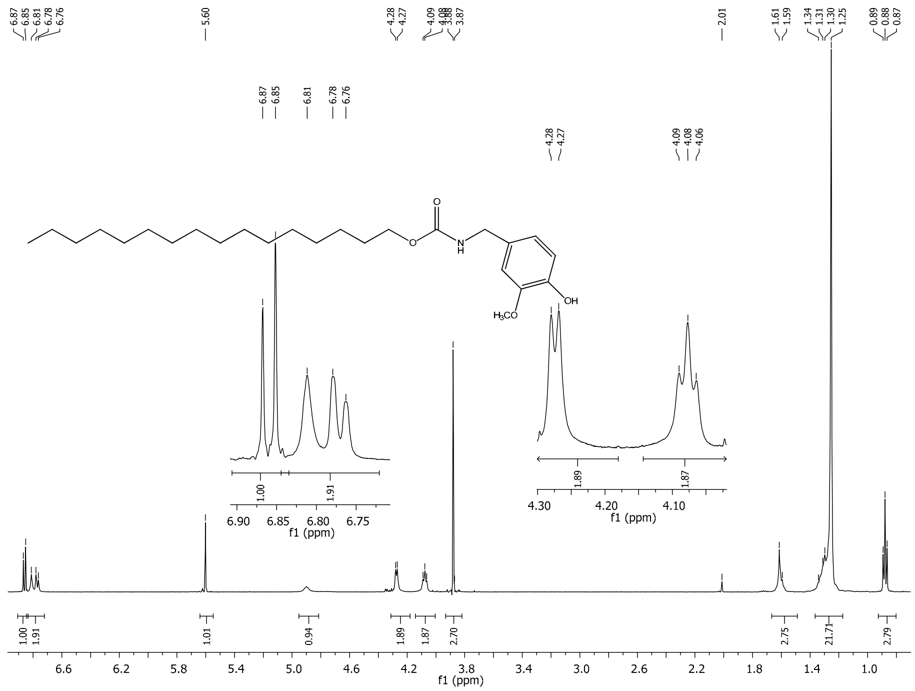


Figure S18. $^{1}$H NMR of **3e** carbamate

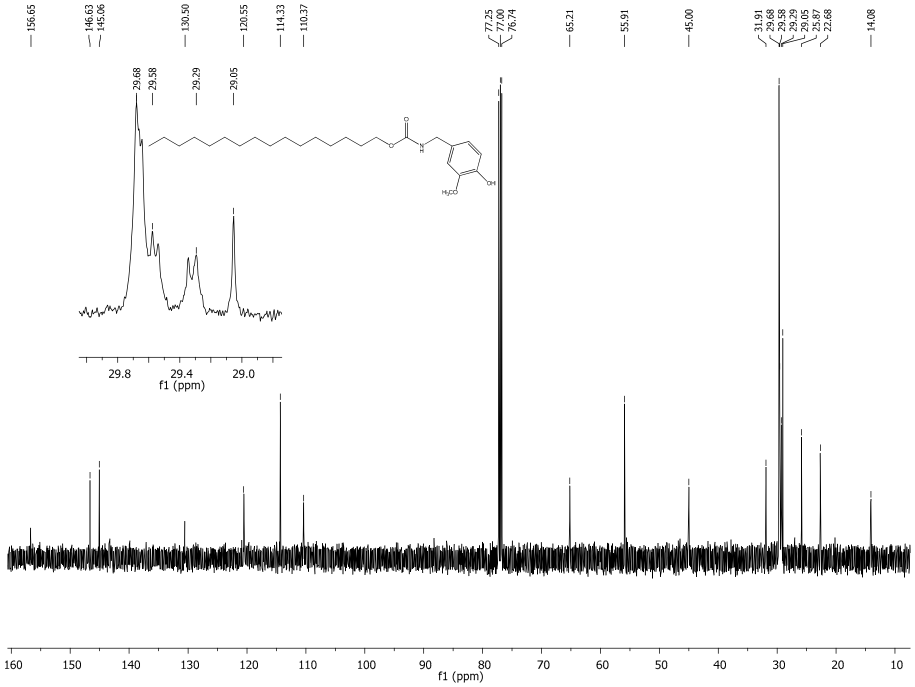


Figure S19. $^{13}$C NMR of **3e** carbamate

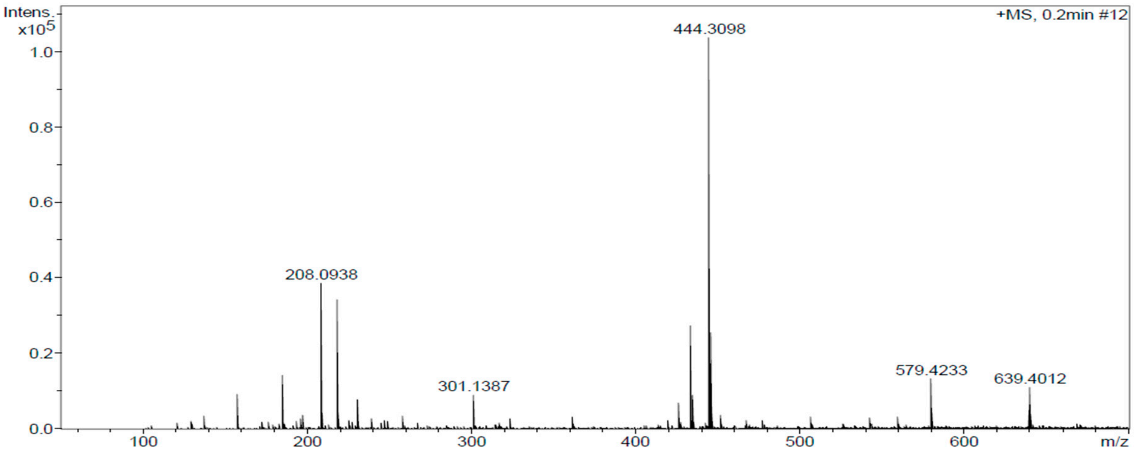


Figure S20. HR-MS of **3e** carbamate

Synthesis of the new ureas

The ureas were synthesized and purified by following a procedure similar to that employed for the carbamates. Hexadecyl isocyanate **4** and the alkyl or aryl amines **2** reacted to form

ureas **5**. The ureas were afforded as a white solid with a yield greater than 90% (see Supplementary Material and Scheme 2).

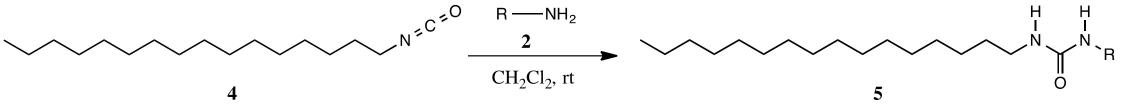


**5a**; R= $-CH_2CH_2N(CH_3)_2$
**5b**; R= -(2-HOPh)
**5c**; R= -(2-$H_2$NPh)
**5d**; R= $-CH_2CH_2OH$
**5e**; R= $-CH_2$(3-$CH_3$O-4-HOPh)

Scheme S2. Synthesis of ureas **5** starting from hexadecyl isocyanate **4** and amines **2**.

*N-Hexadecyl-N-(N,N-dimethylaminoethyl)urea **5a***

The urea **5a** was obtained as white solid, 379 mg (88 %) yield; Table 2; M.p.: 84-85 °C; IR (ATR): ν 3352, 3316 (NH), 2917, 2851 (H-CH-), 1618, 1578 (O=C-NH) $cm^{-1}$; $^{1}$H NMR (500 MHz, $Cl_3CD$): δ = 0.88 (t, 3H, *J*=6.5 Hz, $CH_3R$), 1.25 (br, 26H, $(CH_2)_{13}$), 1.47 (q, 2H, *J*=6.5 Hz, $RCH_2C$), 2.23 (s, 6H, $(CH_3)_2N$), 2.41 (t, 2H, *J*=6 HZ, $CH_2N(CH_3)_2$), 3.14 (c, 2H, *J*=6 Hz, $RCH_2NHCO$), 3.24 (c, 2H, $CONHCH_2$), 4.99 (br, 2H, NH) ppm; $^{13}$C NMR (125 MHz, $Cl_3CD$): δ = 158.8 (NCON), 59.1, 45.2, 40.5, 38.1, 31.9, 30.2, 29.6, 29.5, 26.9, 22.6, 14.0 ppm;  HR-MS (m/z) experimental molecular weight (M+1) 356.3651 g/mol. Calculated molecular weight 356.3640 g/mol.

*N-Hexadecyl-N-(2-hydroxyphenyl)urea **(5b)***

The **5b** urea was obtained as white solid, 587 mg (98 %) yield; Table 2; M.p.: 74-75 °C; IR (ATR): ν 3388, 3327 (NH, OH), 2913, 2846 (H-CH-), 1629 y 1560 (O=C-NH) $cm^{-1}$. $^{1}$H NMR (500 MHz, $Cl_3CD$): δ = 0.88 (t, 3H, *J*=5 Hz, $CH_3R$), 1.26 (br, 26H, $(CH_2)_{13}$), 1.49 (q, 2H, *J*=5 Hz, $RCH_2C$), 3.17 (t, 2H, $RCH_2NHCO$), 6.40 (d, 1H, *J*=5 Hz, $H_5$Ar), 6.73 (t,

1H, *J*=5 Hz, $H_4Ar$), 6.84 (d, 1H, *J*=5 Hz, $H_3Ar$), 7.35 (d, 1H, *J*=5 Hz, $H_6Ar$), 8.0 (br, 1H, NH), 9.97 (br, 1H, OH) ppm; $^{13}C$ NMR (125 MHz, $Cl_3CD$): δ = 156.3, 146.1, 127.1, 122.1, 119.2, 118.6, 116.1, 29.1, 28.6-28.2, 25.9, 21.6, 13.1 ppm; HR-MS (m/z) experimental molecular weight (Sodium salt) 399.2989 g/mol. Calculated molecular weight 399.2987 g/mol.

*N-Hexadecyl-N-(2-aminophenyl)urea **(5c)***

The **5c** urea was obtained as white solid, 528 mg (88 %) yield; Table 2; M.p.: 109-110 ºC; IR (ATR): ν 3280 (NH), 2920, 2851 (H-CH-), 1636, 1556 (O=C-NH) $cm^{-1}$; $^1H$ NMR (500 MHz, $Cl_3CD$): δ = 0.88 (t, 3H, *J*=5.0 Hz, $CH_3R$), 1.26 (br, 26H, $(CH_2)_{13}$), 1.48 (q, 2H, *J*=5 Hz, $RCH_2C$), 3.17 (q, 2H, *J*=5 Hz, $RCH_2NHCO$), 4.19 (br, 2H, $NH_2$), 5.71 (br, 1H, NH), 6.66 (dd, 1H, *J*=9 Hz, $H_5Ar$), 6.72 (d, 1H, *J*=9 Hz, $H_3Ar$), 6.88 (t, 1H, *J*=9 Hz, $H_4Ar$), 7.23 (d, 1H, *J*=9 Hz, $H_6Ar$), 7.23 (br, 1H, NH) ppm; $^{13}C$ NMR (125 MHz, $Cl_3CD$): δ = 156.0, 140.1, 124.6, 124.3, 124.0, 123.2, 117.4, 115.7, 29.3, 28.7-28.6, 28.4, 26.0, 21.7, 13.3 ppm; HR-MS (m/z) experimental molecular weight (Sodium salt) 398.3142 g/mol. Calculated molecular weight 398.3147 g/mol.

*N-Hexadecyl-N-(2-hydroxyethyl)urea **(5d)***

The **5d** urea was obtained as white solid, 503 mg (90 %) yield; Table 2; M.p.: 104-105 ºC; IR (ATR): ν 3318, 3176 (NH, OH), 2912, 2849 (H-CH-), 1617, 1591 (O=C-NH) $cm^{-1}$; $^1H$ NMR (500 MHz, $Cl_3CD$): δ = 0.97 (t, 3H, *J*=5 Hz, $CH_3R$), 1.35 (br, 26H, $(CH_2)_{13}$), 1.54 (q, 2H, *J*=5 Hz, $RCH_2C$), 3.20 (q, 2H, *J*=5 Hz, $RCH_2NHCO$), 3.35 (c, 2H, *J*=5 Hz, $CONHCH_2$), 3.70 (t, 2H, *J*=5 Hz, $CH_2OH$), 4.50 (br, 1H, OH); 5.64 (br, 1H, NH), 5.84 (br,

1H, NH), 5.75 (br, 1H, NH) ppm; $^{13}C$ NMR (125 MHz, $Cl_3CD$): δ = 159.0 (C=O), 62.1, 42.2, 31.1, 29.6-28.5, 26.2, 21.9, 13.4 ppm; HR-MS (m/z) experimental molecular weight (M+1) 329.3187 g/mol. Calculated molecular weight 329.3168 g/mol.

*N-Hexadecyl-N-(4-hydroxy-3-methoxyphenyl)methylurea (**5e**)*

The **5e** urea was obtained as white solid, 234 mg (39 %) yield; Table 2; M.p.: 98-99 ºC; IR (ATR): ν 3515, 3345, 3322 (NH, OH), 2920, 2851 (H-CH-), 1614, 1568 (O=C-NH) $cm^{-1}$.; $^{1}H$ NMR (500 MHz, $Cl_3CD$-DMSO): δ = 0.88 (t, 3H, *J*=5 Hz, $CH_3R$), 1.25 (br, 26H, $(CH_2)_{13}$), 1.46 (br, 2H, $RCH_2C$), 3.14 (q, 2H, *J*=5 Hz, $RCH_2N$), 3.86 (s, 3H, $CH_3O$), 4.26 (q, 2H, *J*=5 Hz, $NCH_2Ar$), 5.64 (br, 1H, NH), 5.29 (br, 1H, NH), 6.75 (d, 1H, *J*=10 Hz, $H_6Ar$), 6.82 (d, 1H, *J*=10 Hz, $H_5Ar$), 6.83 (s, 1H, $H_2Ar$), 7.45 (s, 1H, OH) ppm; $^{13}C$ NMR (125 MHz, $Cl_3CD$): δ = 158.5 (C=O), 147.8, 145.7, 132.2, 120.0, 115.6, 112.0, 56.0, 43.3, 31.8, 30.6-29.3, 26.9, 22.6, 14.5 ppm; HR-MS (m/z) experimental molecular weight (Sodium salt) 443.3248 g/mol. Calculated molecular weight 443.3249 g/mol.

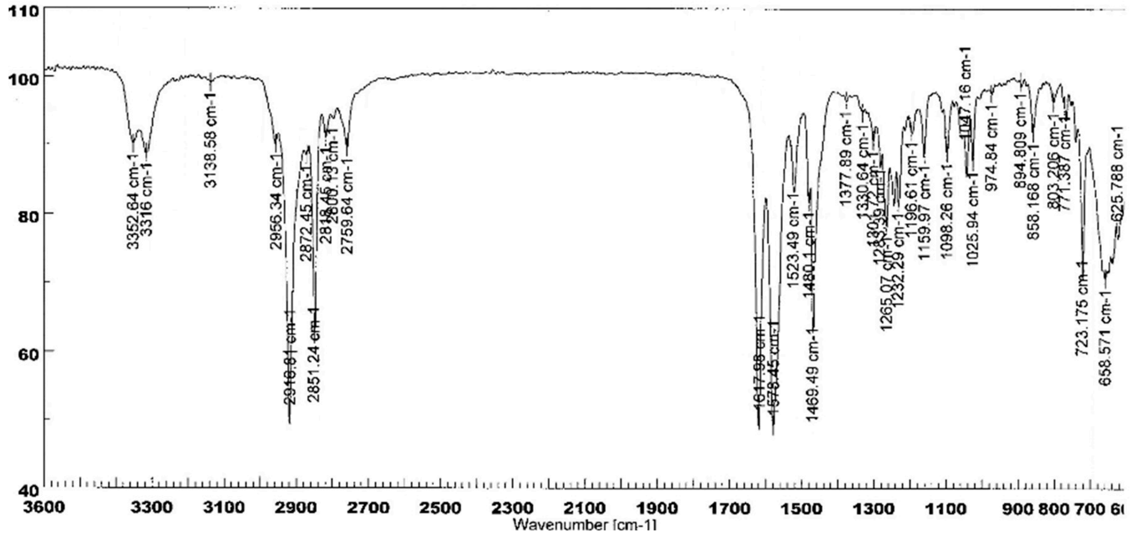


Figure S21. FT-IR of **5a** carbamate

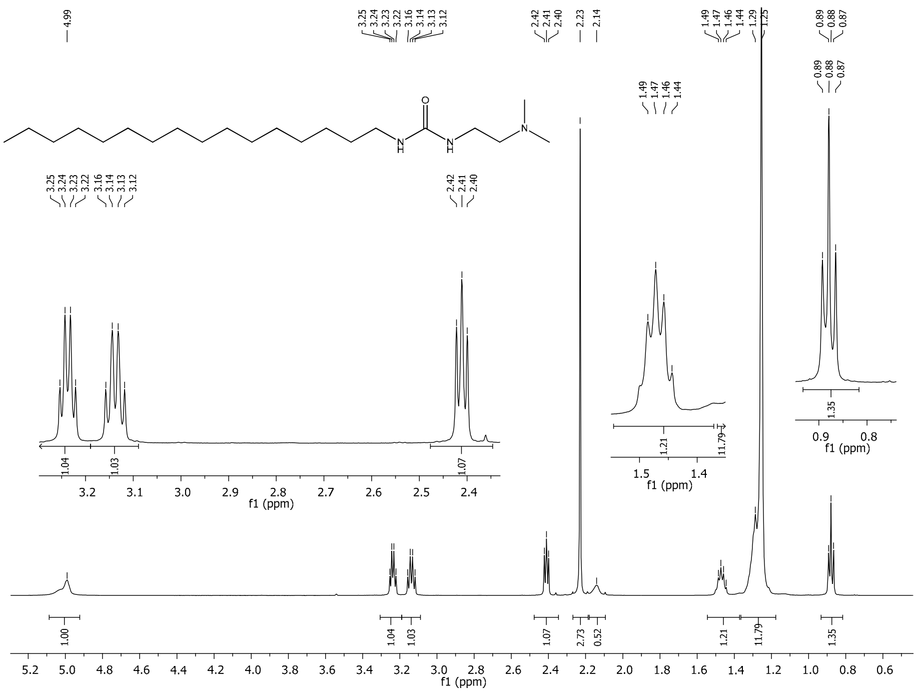

Figure S22.$^{1}$H NMR of **5a** urea

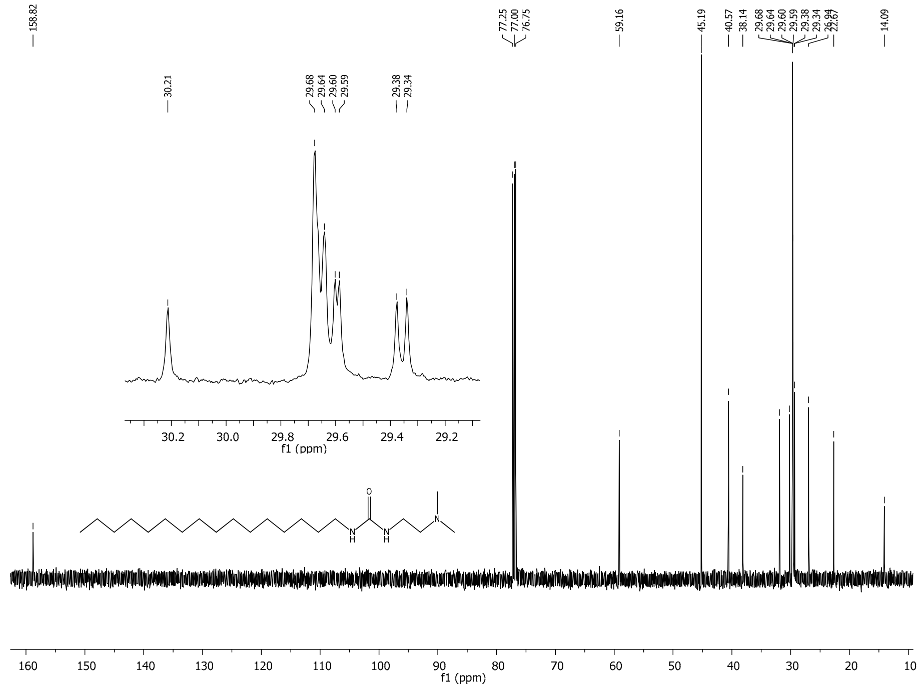

Figure S23.$^{13}$C NMR of **5a** urea

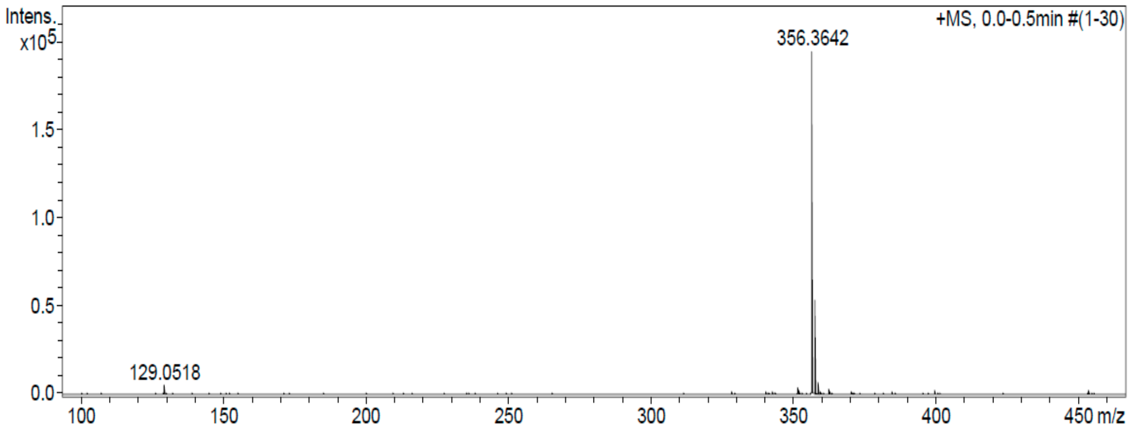


Figure S24. HR-MS of **5a** urea

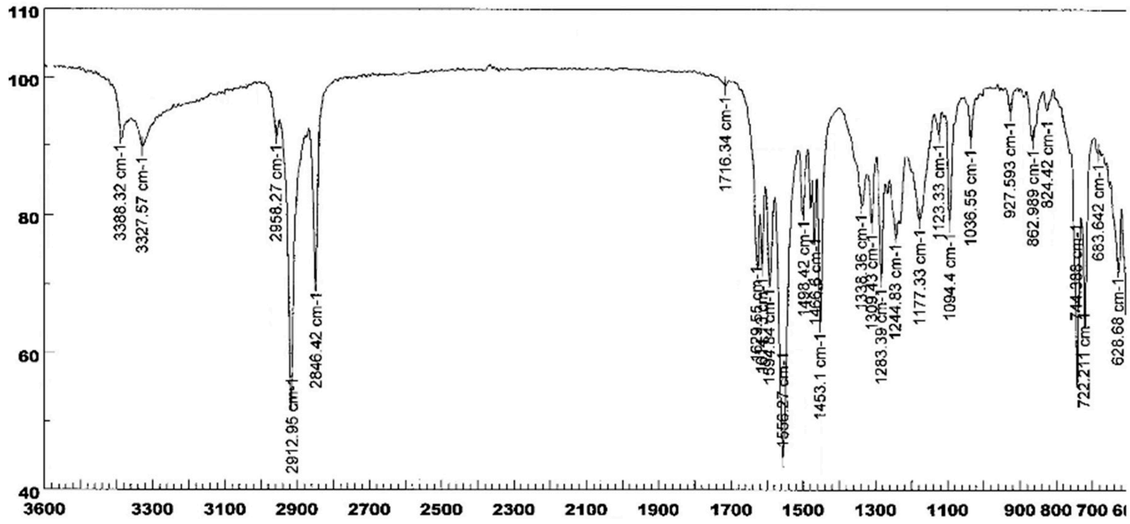


Figure S25. FT-IR of **5b** carbamate

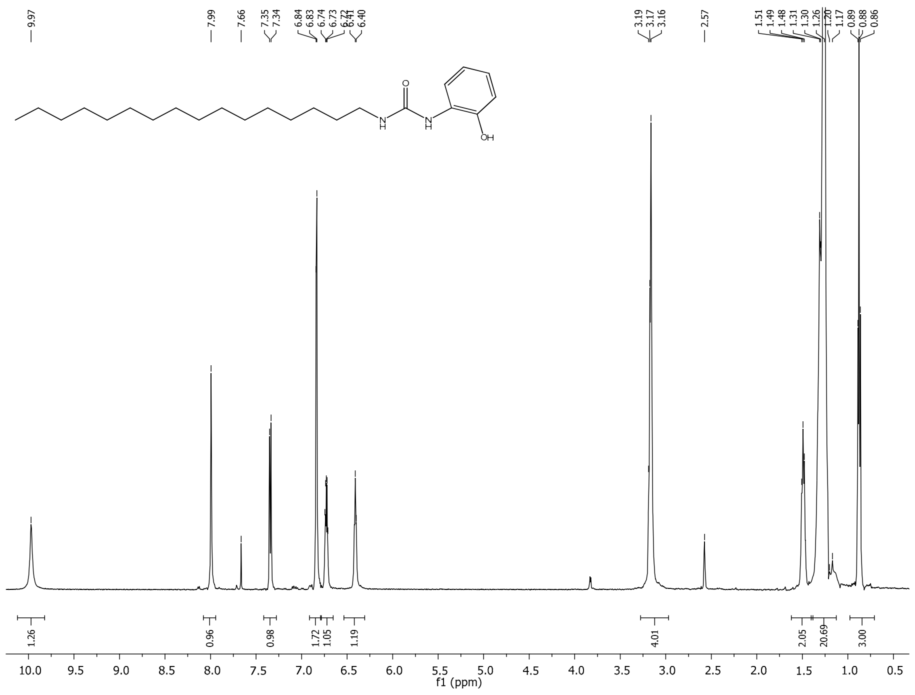

Figure S26.$^{1}$H NMR of **5b** urea

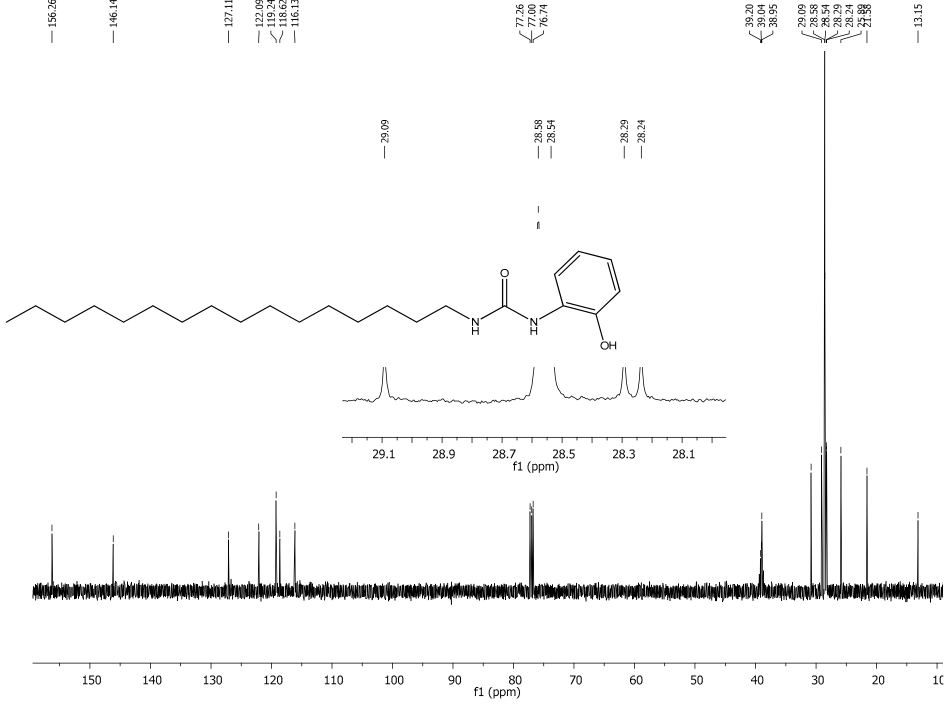

Figure S27. $^{13}$C NMR of **5b** urea

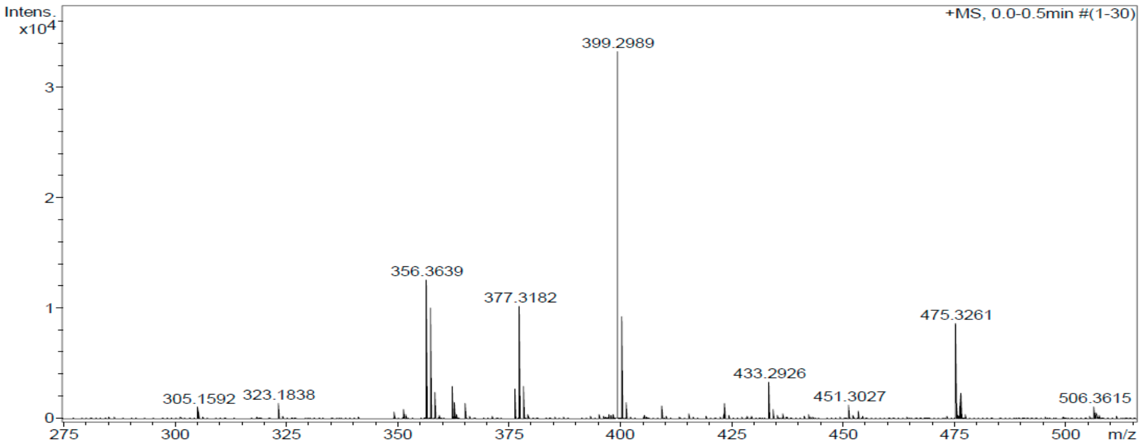


Figure S28. HR-MS of **5b** urea.

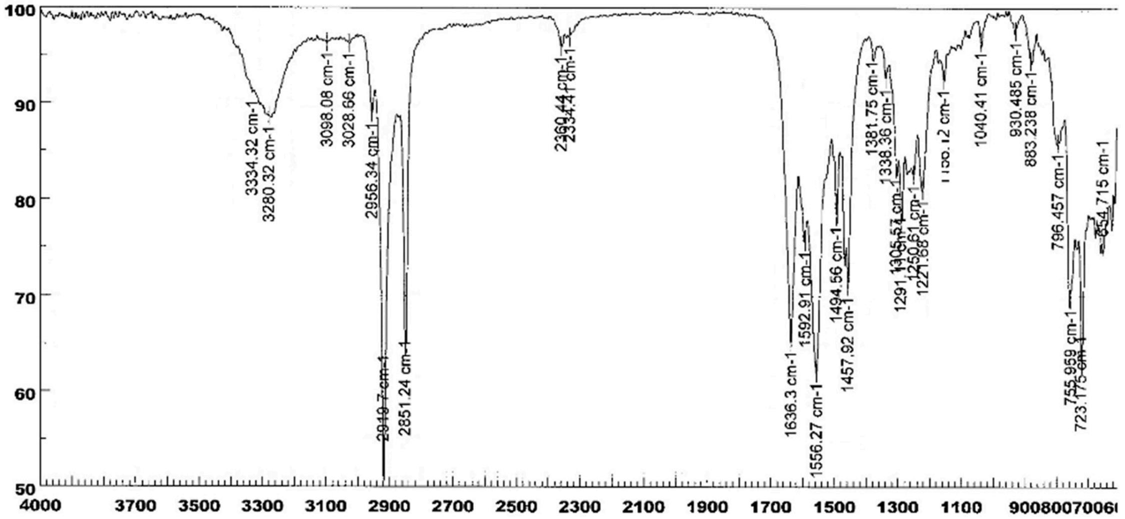


Figure S29. FT-IR of **5c** carbamate.

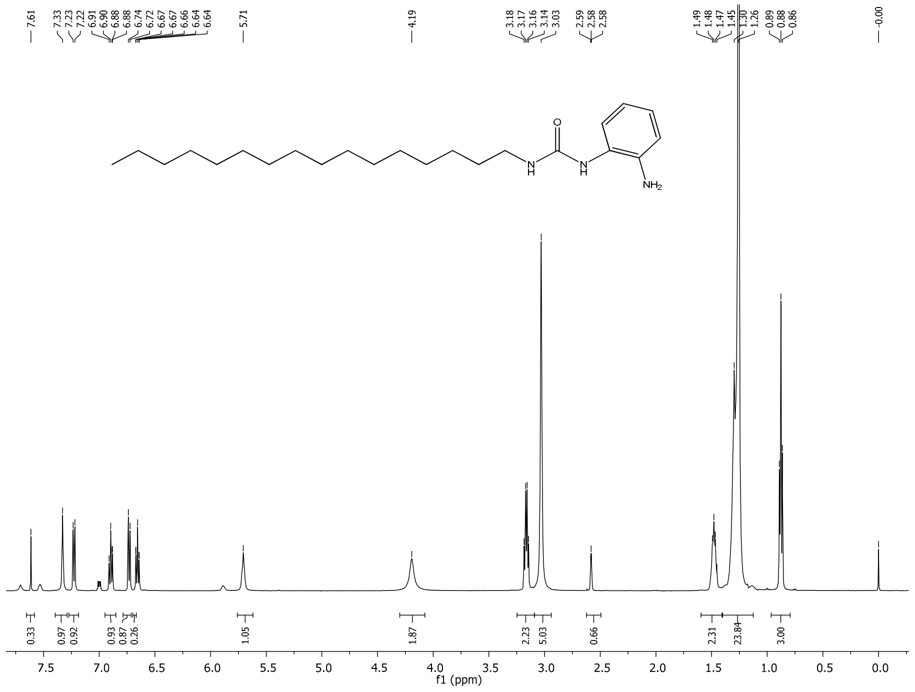

Figure S30.$^{1}$H NMR of **5c** urea.

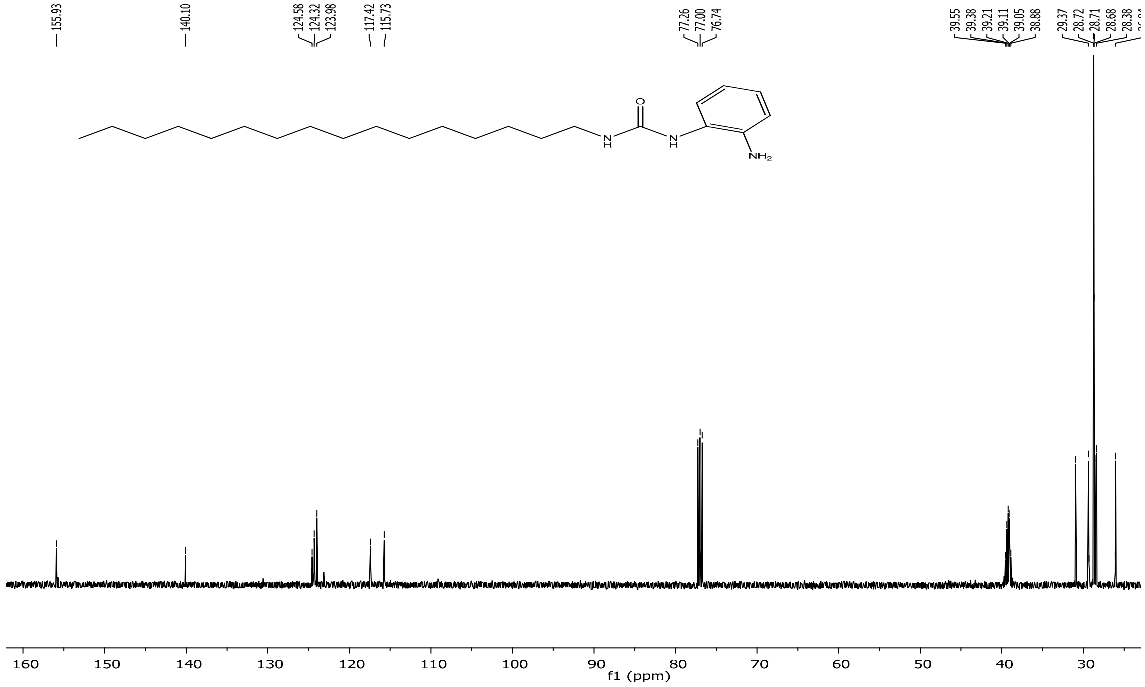

Figure S31. $^{13}$C NMR of **5c** urea.

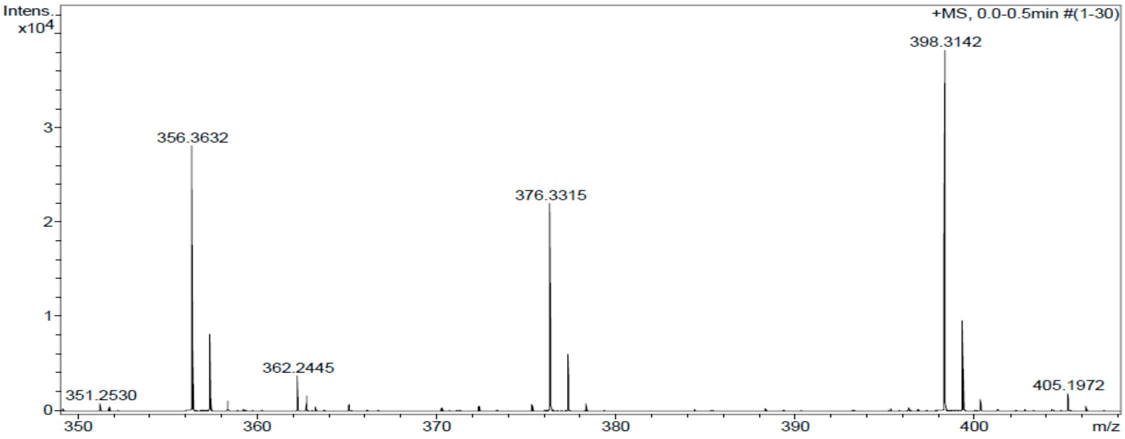


Figure S32. HR-MS of **5c** urea.

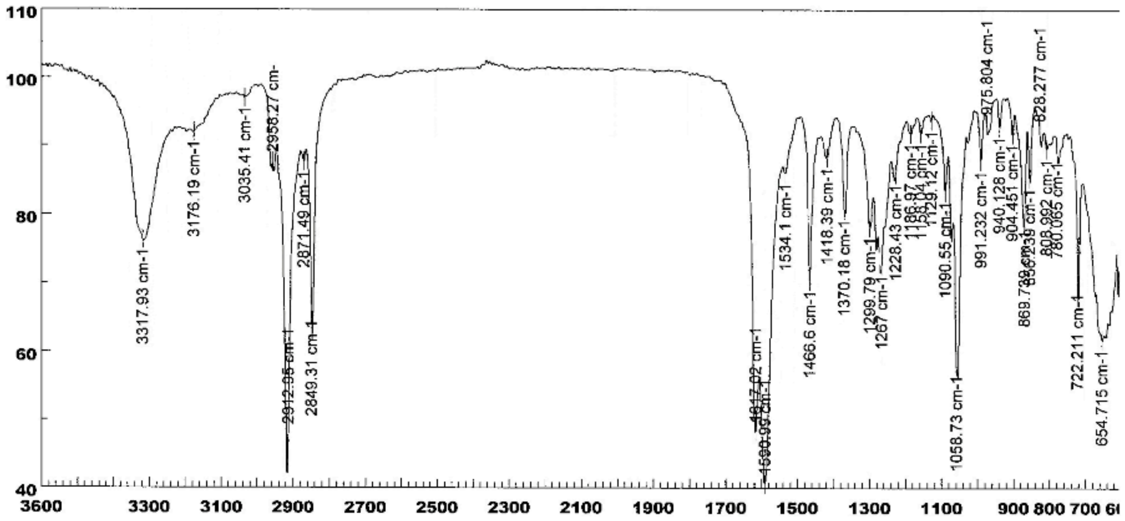


Figure S33. FT-IR of **5d** carbamate.

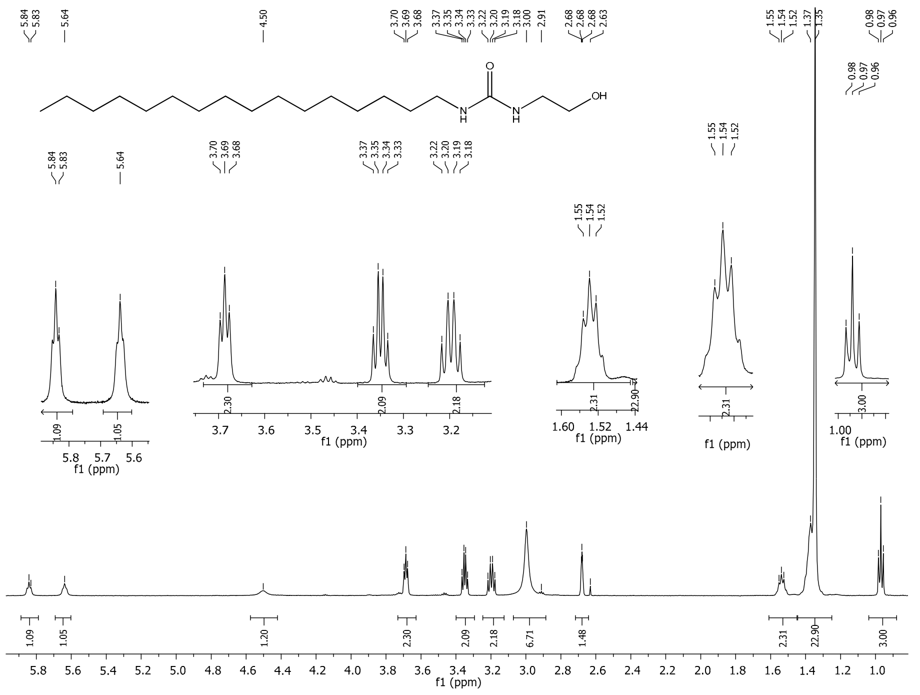

Figure S34. $^{1}$H NMR of **5d** urea.

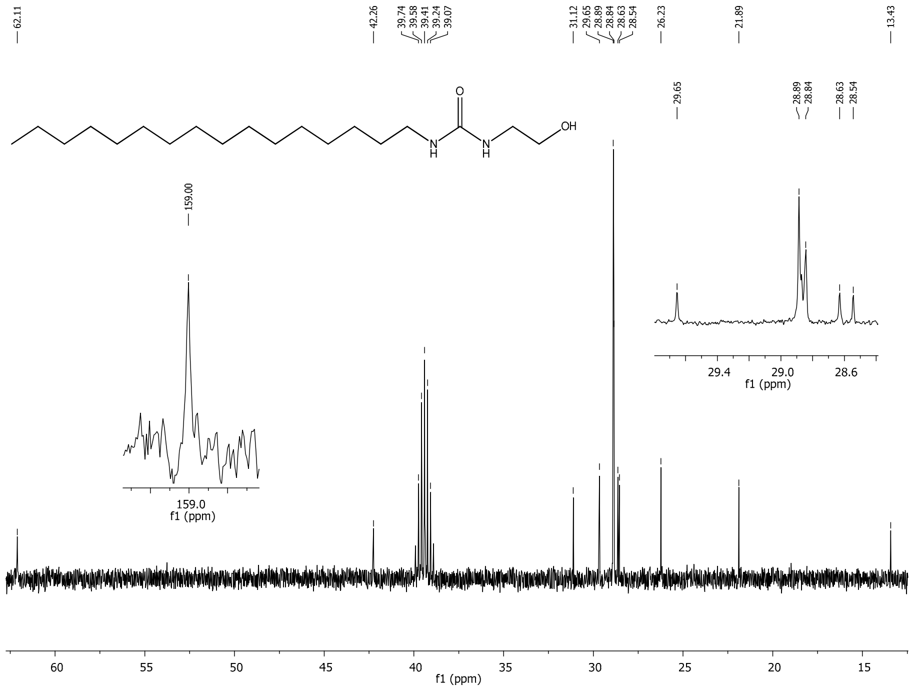

Figure S35. $^{13}$C NMR of **5d** urea.

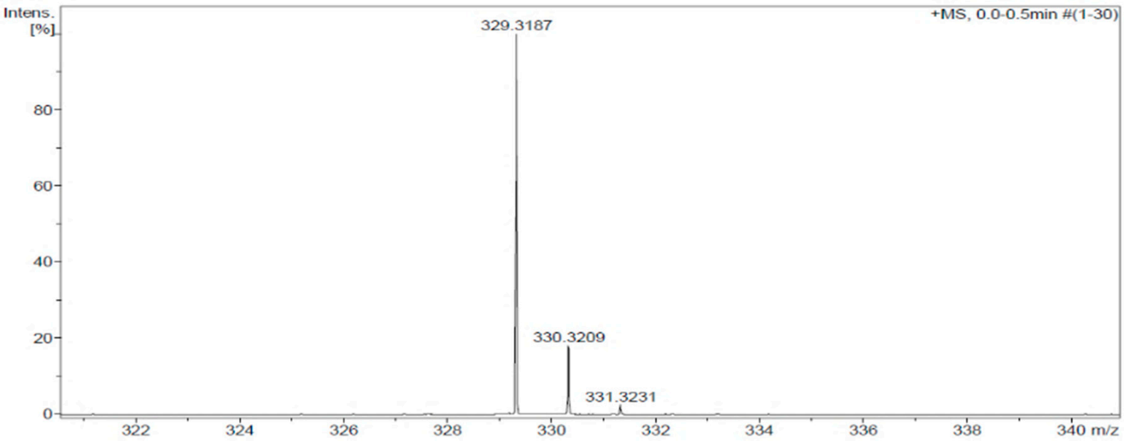


Figure S36. HR-MS of urea **5d**

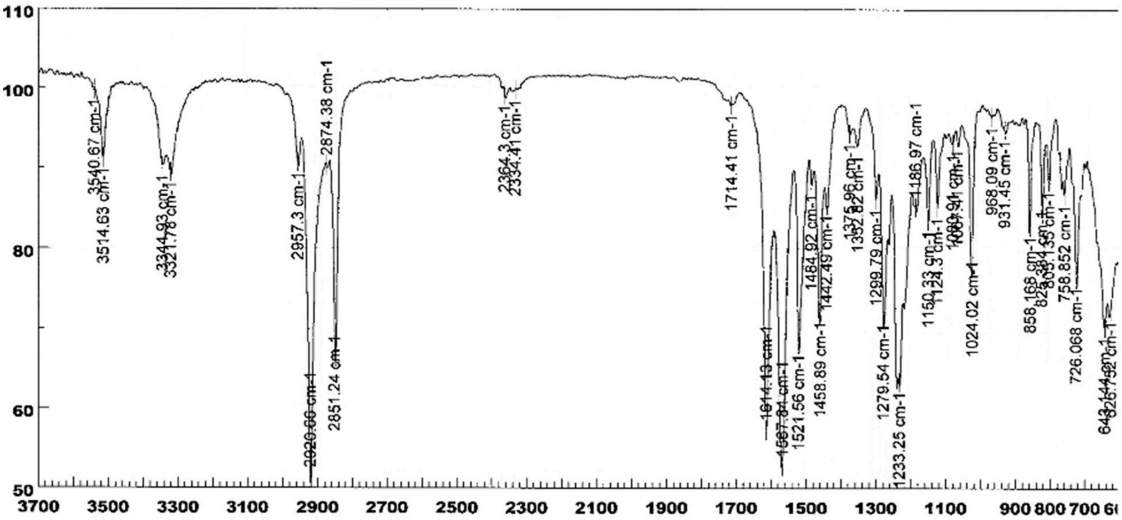


Figure S37. FT-IR of **5e** carbamate.

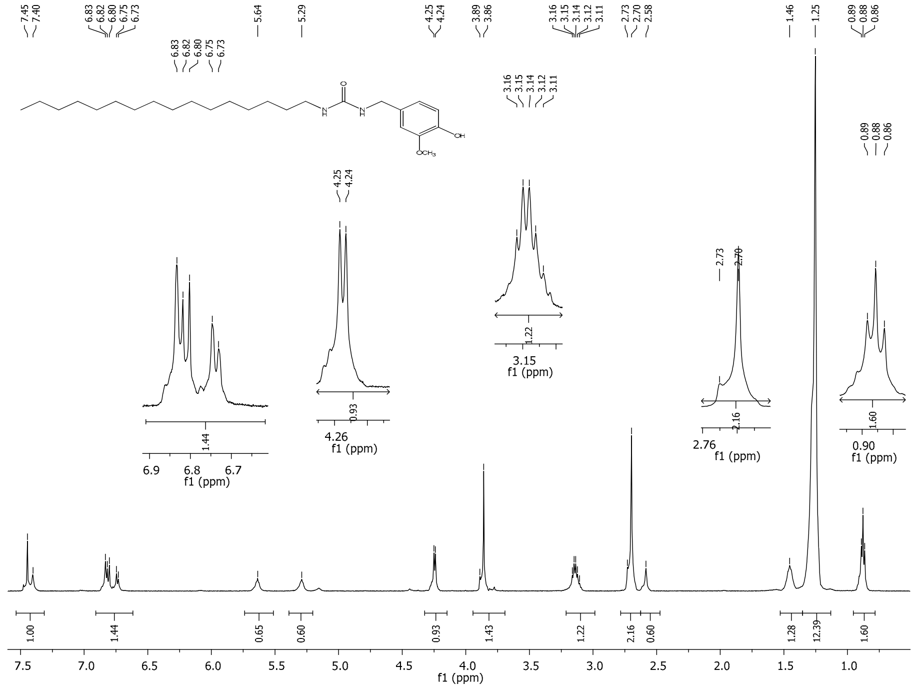

Figure S38. $^{1}$H NMR of **5e** urea.

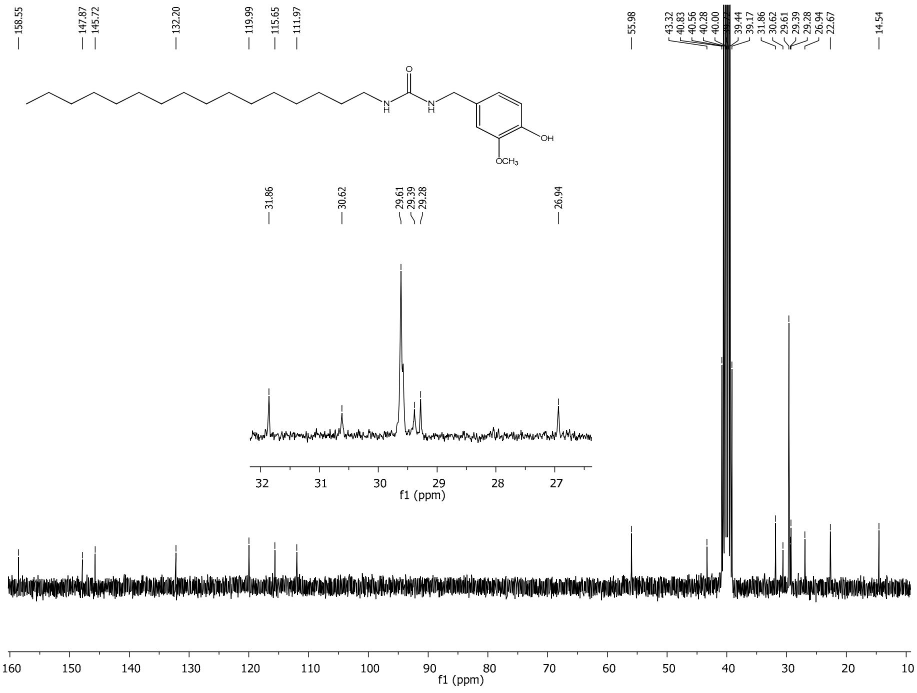

Figure S39. $^{13}$C NMR of **5e** urea.

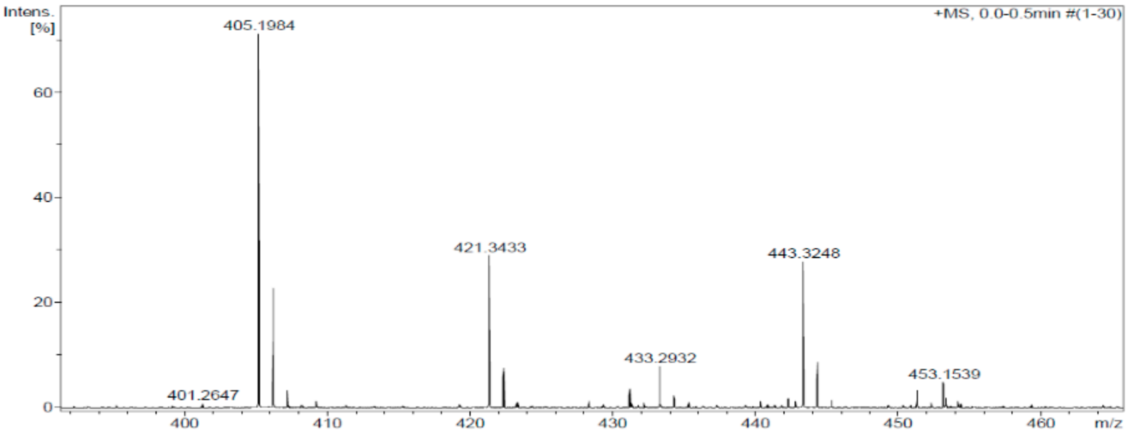


Figure S40. HR-MS of **5e** urea.

Table S1. Results of **3** carbamates in FT-IR, $^{1}$H NMR, and HR-MS

| Carbamate<br>α<br>R=$CH_3$-$(CH_2)_{14}$-$CH_2$- | ν: C=O<br>($cm^{-1}$) | δ: (ppm) | | | | | | mp<br>(°C) | (m/z)<br>Experimental<br>(Calculated) |
|---|---|---|---|---|---|---|---|---|---|
| **3a**<br>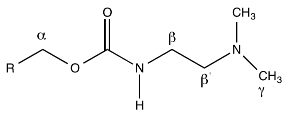 | 1685 | 4.04<br>($H\alpha$) | 3.65<br>($H\beta$) | 3.28<br>($H\beta$') | 2.92<br>($H\gamma$) | | | 79-80 | 357.3486<br>(357.3481)<br>M+1 |
| **3b**<br>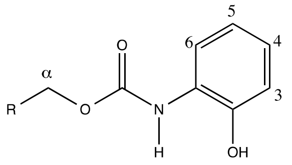 | 1681 | 4.18<br>($H\alpha$) | 7.04<br>($H_3$) | 6.89<br>($H_4$) | 6.97<br>($H_5$) | 7.19<br>($H_6$) | | 77-78 | 400.2822<br>(400.2827)<br>Sodium salt |
| **3c**<br>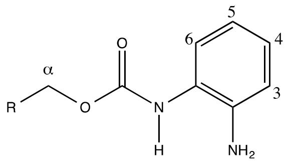 | 1681 | 4.14<br>($H\alpha$) | 7.14<br>($H_3$) | 6.78<br>($H_4$) | 7.02<br>($H_5$) | 7.15<br>($H_6$) | | 84-85 | 399.2982<br>(399.2987)<br>Sodium salt |
| **3d**<br>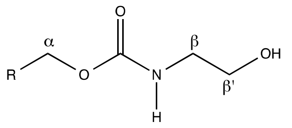 | 1691<br>1549 | 4.04<br>($H\alpha$) | 3.30<br>($H\beta$) | 3.66<br>($H\beta$') | | | | 73-74 | 352.2842<br>(352.2827)<br>Sodium salt |
| **3e**<br>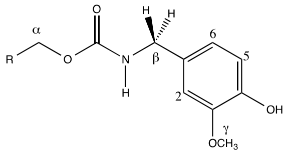 | 1683<br>1534 | 4.08<br>($H\alpha$) | 4.28<br>($H\beta$) | 6.81<br>($H_2$) | 6.87<br>($H_5$) | 6.78<br>($H_6$) | 3.88<br>($H\gamma$) | 79-80 | 421.3201<br>(421.3192) |

Table S2. Results of **5** ureas in FT-IR, $^{1}$H NMR, and HR-MS

| Urea<br>α<br>$R=CH_3-(CH_2)_{14}-CH_2-$ | ν:<br>C=O<br>($cm^{-1}$) | δ: (ppm) | | | | | | mp<br>(°C) | (m/z)<br>Experimental<br>(Calculated) |
|---|---|---|---|---|---|---|---|---|---|
| **5a**<br>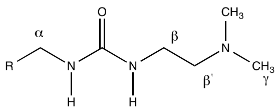 | 1618<br>1578 | 3.14<br>(Hα) | 3.24<br>(Hβ) | 2.41<br>(Hβ') | 2.23<br>(Hγ) | | | 84-85 | 356.3651<br>(356.3640)<br>M+1 |
| **5b**<br>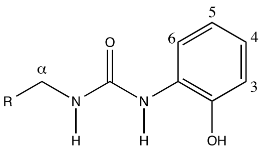 | 1629<br>1560 | 3,17<br>(Hα) | 6.84<br>($H_3$) | 6.73<br>($H_4$) | 6.40<br>($H_5$) | 7.35<br>($H_6$) | | 74-75 | 399.2989<br>(399.2987)<br>Sodium salt |
| **5c**<br>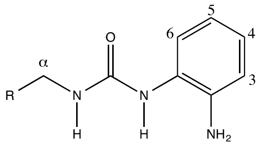 | 1636<br>1556 | 3,17<br>(Hα) | 6.72<br>($H_3$) | 6.88<br>($H_4$) | 6.66<br>($H_5$) | 7.23<br>($H_6$) | | 109-110 | 398.3142<br>(398.3147)<br>Sodium salt |
| **5d**<br>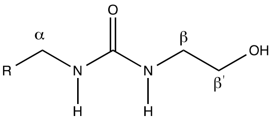 | 1617<br>1591 | 3.20<br>(Hα) | 3.35<br>(Hβ) | 2.70<br>(Hβ') | | | | 104-105 | 329.3187<br>(329.3168)<br>M+1 |
| **5e**<br>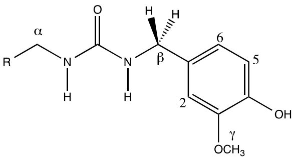 | 1614<br>1568 | 3.14<br>(Hα) | 4.26<br>(Hβ) | 6.83<br>($H_2$) | 6.82<br>($H_5$) | 6.75<br>($H_6$) | 3.86<br>(Hγ) | 98-99 | 443.3248<br>(443.3249)<br>M+1 |

Gelation test

The gelation properties were examined for each compound in relation to four solvents. Briefly, a sample of 1 mL of solvent was put in a capped vial and weighed. The respective compound was added to a solvent in quantities of 2 mg in 2 mg until saturation was reached. The mixture was heated in a thermal bath until the solid was dissolved and a clear solution was obtained. The solution was cooled until gel formation and its temperature was registered. Finally, the test vial was inverted to assure that there was no flow of the organic solvent out of the gel.[58] The gel was weighed and the loss of solvent was calculated. The gel was then heated and observed to record the temperature at which it broke down. The experiments were performed in triplicate.

Scanning electron microscopy

A gel sample was put in a cooper sample holder. The sample was cooled with liquid nitrogen and sputtered with platinum-gold. The samples were placed in a JEOL scanning electron microscope (model JSM 7800F, USA) at 1 kV.

Table S3. Comparative gelation properties of **3** carbamates (minimum gelation concentration, measured as the percentage of weight) with different organic solvents ($CH_3$-$(CH_2)_{14}$-$CH_2$-(OCONH)-$R^1$).

| Carbamates | Solvents | | | |
|---|---|---|---|---|
| | Carbon tetrachloride | Xylene | Toluene | 1,4-Dioxane |
| **3a** | I | G (15.17) | G (4.46) | G (7.03) |
| **3b** | I | I | I | G (10.33) |
| **3c** | I | I | I | G (9.77) |
| **3d** | I | G (3.6) | G (6.8) | G (6.5) |
| **3e** | I | I | I | G (5.1) |

G, gel; I, insoluble.

Table S4. Comparative gelation properties of **5** ureas (minimum gelation concentration, measured as the percentage of weight) with different organic solvents ($CH_3$-$(CH_2)_{14}$-$CH_2$-(HNCONH)-$R^1$).

| Ureas | Solvents | | | |
|---|---|---|---|---|
| | Carbon tetrachloride | Xylene | Toluene | 1,4-Dioxane |
| **5a** | G (2.8) | I | I | I |
| **5b** | G (2.1) | I | I | G (1.0) |
| **5c** | G (1.7) | G (0.5) | G (0.8) | G (4.9) |
| **5d** | I | G (1.4) | G (1.1) | G (0.9) |
| **5e** | I | I | G (3.2) | G (1.2) |

G, gel; I, insoluble.

Table S5. Gel formation temperatures and breaking temperatures (Tg)/(Tb) for carbamates **3a-3e** and ureas **5a-5e**.

| Carbamates | Ureas | Solvents | | | | | | | |
|---|---|---|---|---|---|---|---|---|---|
| | | Carbon tetrachloride | | Xylene | | Toluene | | 1,4-Dioxane | |
| **3a** | **5a** | - | 15/22 | 26/43 | - | 10/20 | - | 2/20 | - |
| **3b** | **5b** | - | 3/40 | - | - | - | - | 0/23 | 5/20 |
| **3c** | **5c** | - | 1/25 | - | 12/35 | - | 10/55 | 0/23 | 5/35 |
| **3d** | **5d** | - | - | 0/38 | 10/25 | 0/36 | 3/22 | 0/36 | 5/20 |
| **3e** | **5e** | - | - | - | - | - | 2/21 | 10/14 | 0/23 |

Table S6. Chemical shifts (δ ppm) of the N-H bonds of **3a-3e** carbamates and **5a-5e** ureas.

| R ($CH_3$-$(CH_2)_{14}$-$CH_2$-) | Carbamate δ (ppm) N-H bond | Urea δ (ppm) N-H bond |
|---|---|---|
| **3a/5a** | 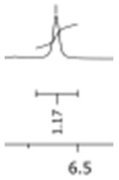  | 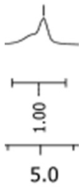  |
| **3b/5b** | 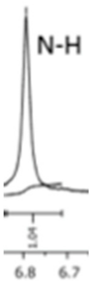  | 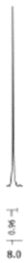  |
| **3c/5c** | 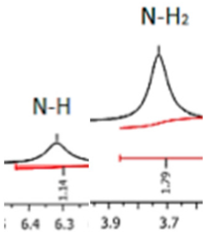  | 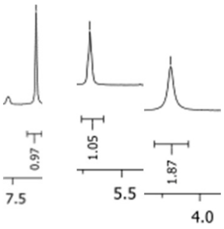  |
| **3d/5d** | 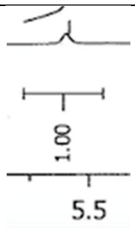  | 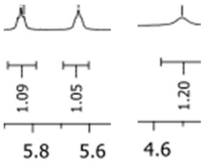  |

| | | |
|---|---|---|
| **3e/5e** | 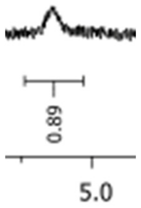 | 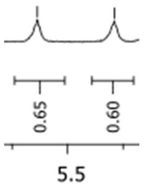 |





Table S7. Solubility parameter, molar volume and the Flory-Huggins parameter for several solvents.

| Solvent | Molar Volume $V_s$ ($cm^3$/mol) | Solubility parameter δ (cal $cm^{-3}$)$^{1/2}$ | Flory-Huggins interaction parameter $\chi_{SP}$ | |
|---|---|---|---|---|
| | | | Carbamate | Urea |
| Xylene | 122.78 | 8.90 | 8.24 | 3.59 |
| Toluene | 106.27 | 8.95 | 7.95 | 3.12 |
| 1,4-Dioxane | 85.53 | 10.02 | 6.42 | 2.39 |
| $CCl_4$ | 96.74 | 8.80 | | 2.96 |

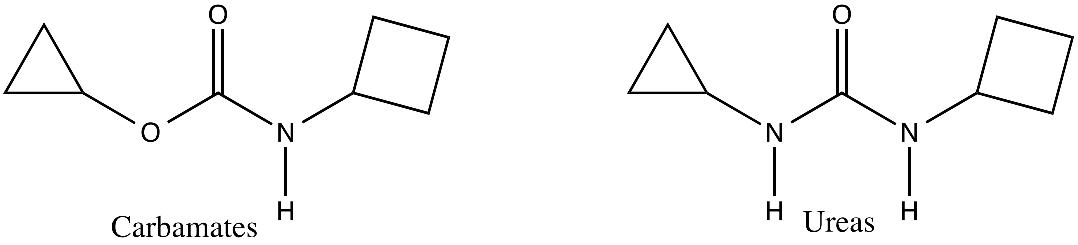


Figure S41. The only difference between the carbamates and the ureas is an additional N-H bond in the latter.

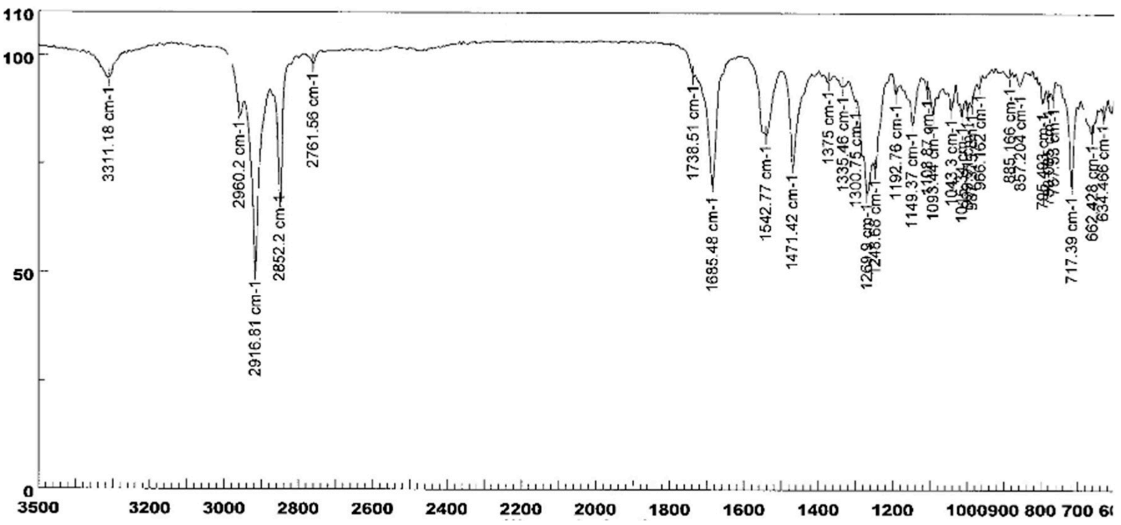


Figure S42. FT-IR spectrum of neat **3a** carbamate.

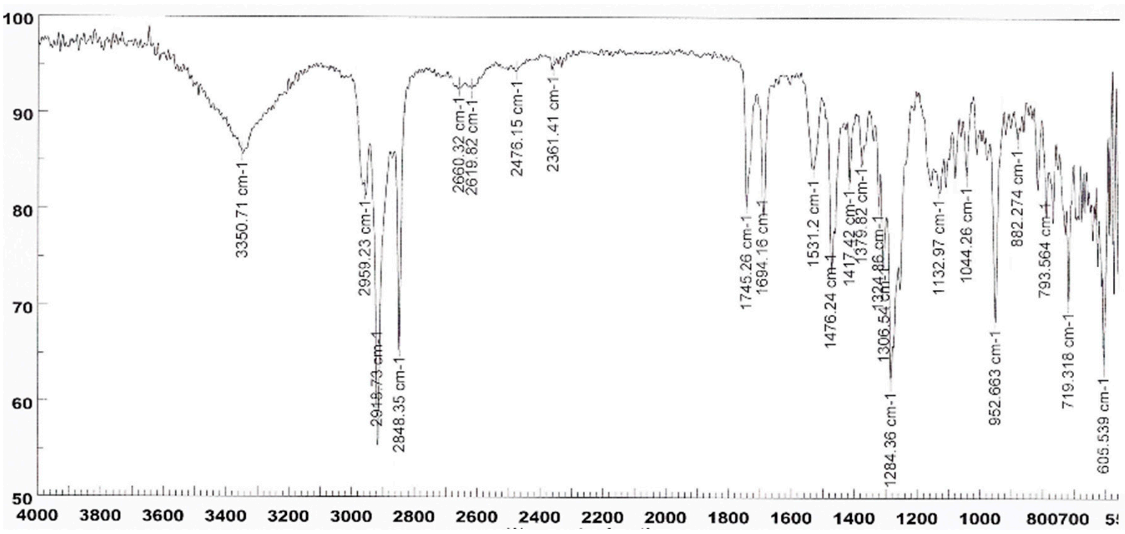


Figure S43. FT-IR spectrum of the gel formed by **3a** carbamate with xylene.

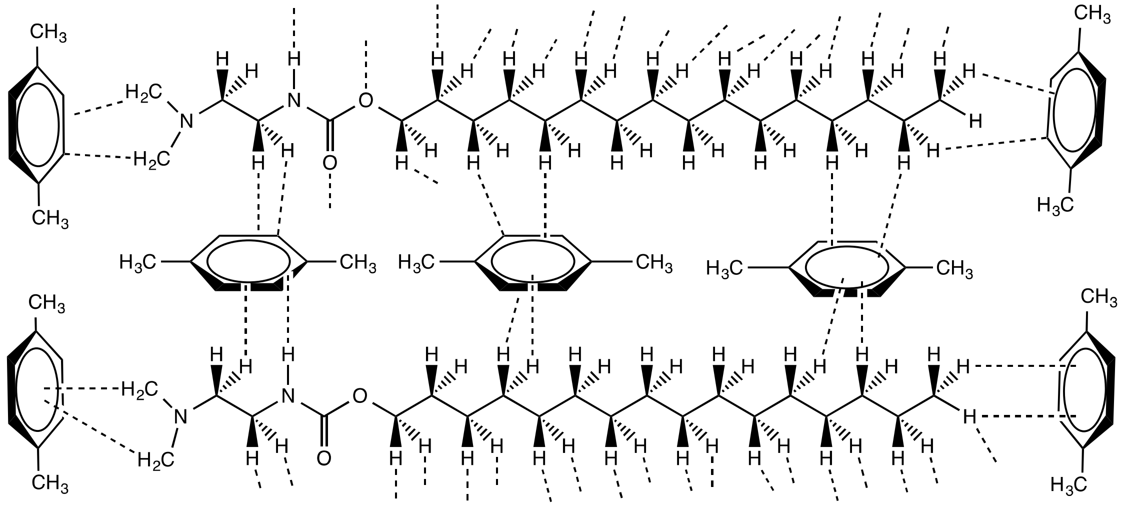

Figure S44. Supramolecular network formed by **3a** carbamate with xylene.

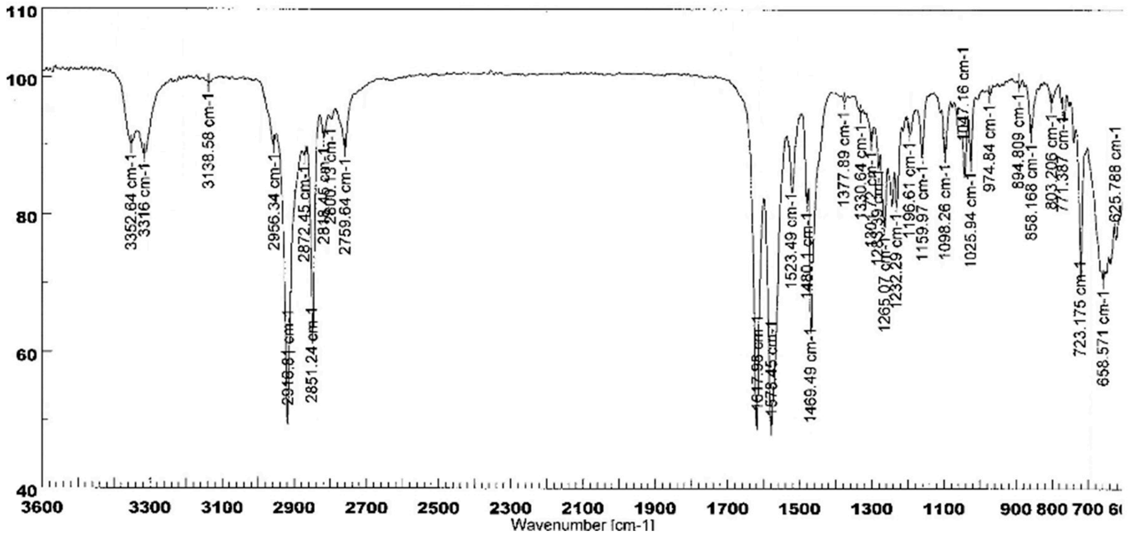


Figure S45. FT-IR spectrum of neat **5a** urea.

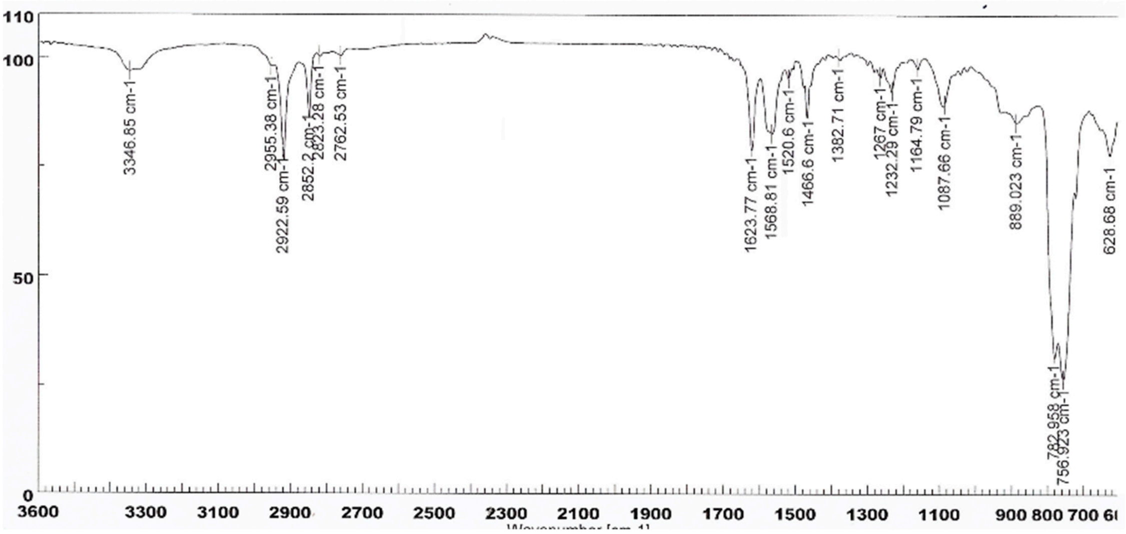


Figure S46. FT-IR spectra of the gel formed by **5a** urea with carbon tetrachloride.

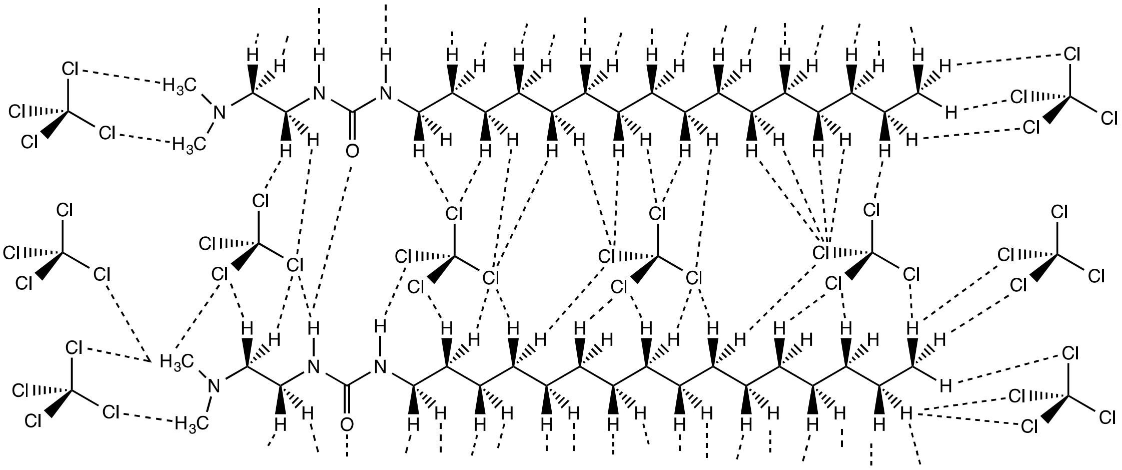

Figure S47. Supramolecular networks formed by **5a** urea with carbon tetrachloride.

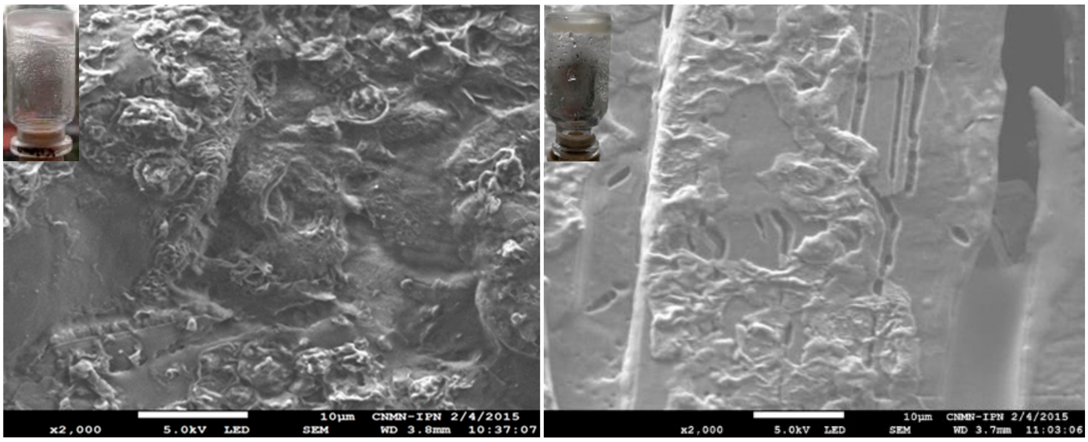


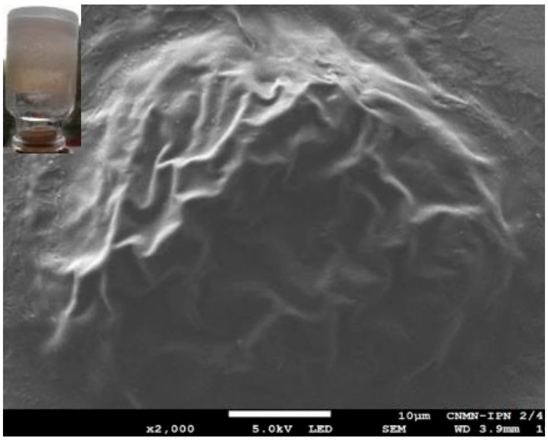


**3a c**arbamate with xylene, toluene and 1,4-dioxane, respectively.

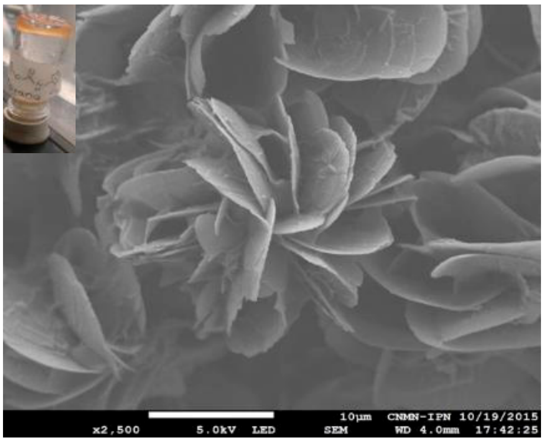


**3b** carbamate with 1,4-dioxane

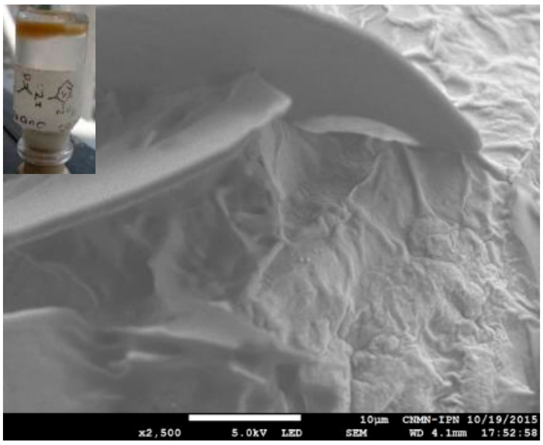


**3c** carbamate with 1,4-dioxane

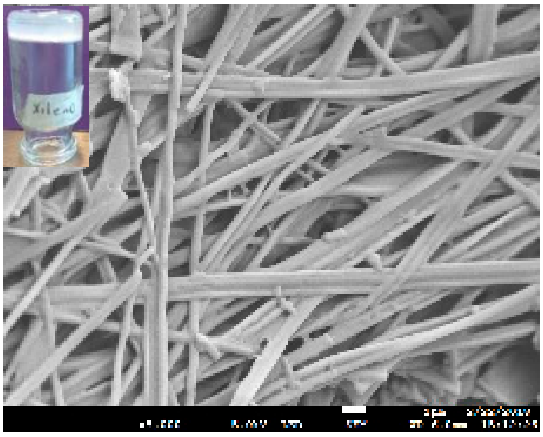

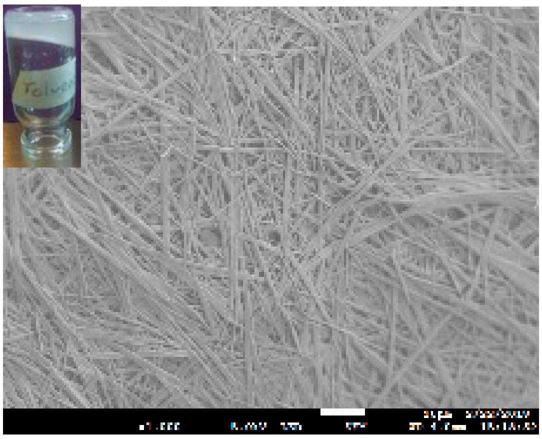

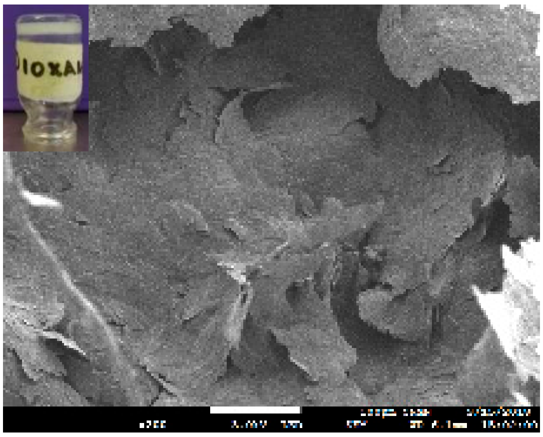

**3d** carbamate with xylene, toluene and 1,4-dioxane, respectively.

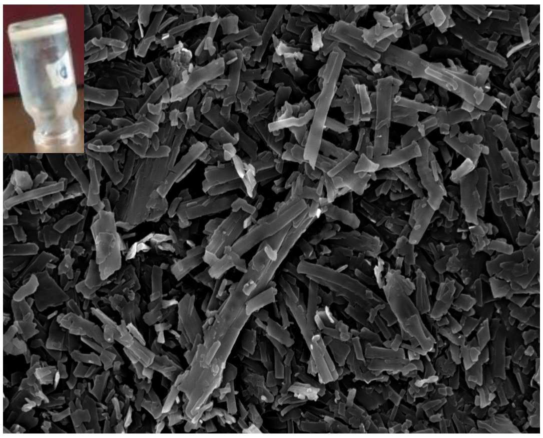

**3e** carbamate with 1,4-dioxane.

Figure S48. Photographs of the flasks and scanning electron microscopy micrographs of the gels obtained by interacting **3a**-**3e** carbamates with different organic solvents.

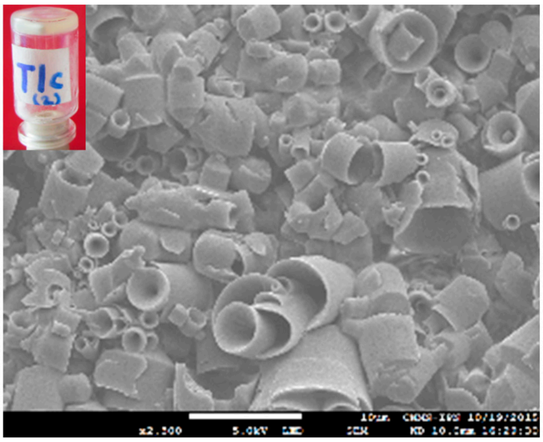

Urea **5a** with carbon tetrachloride

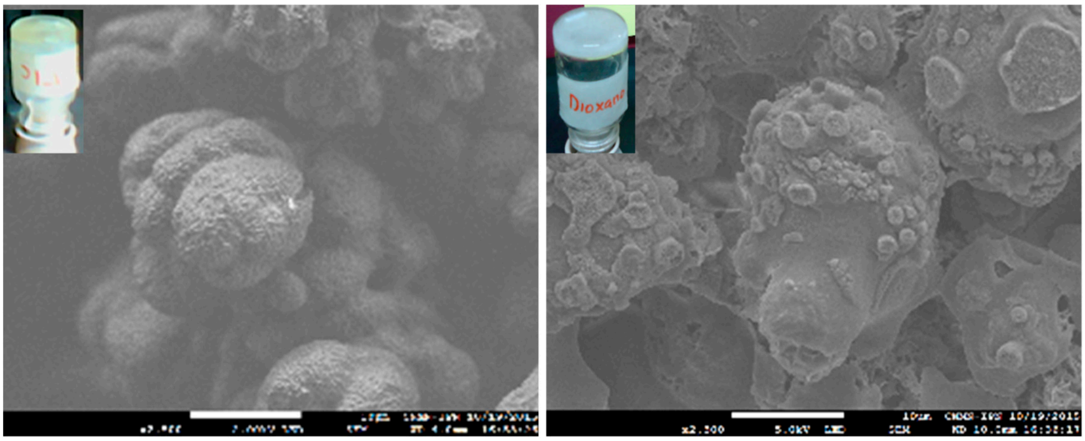


Urea **5b** with carbon tetrachloride and 1,4-dioxane

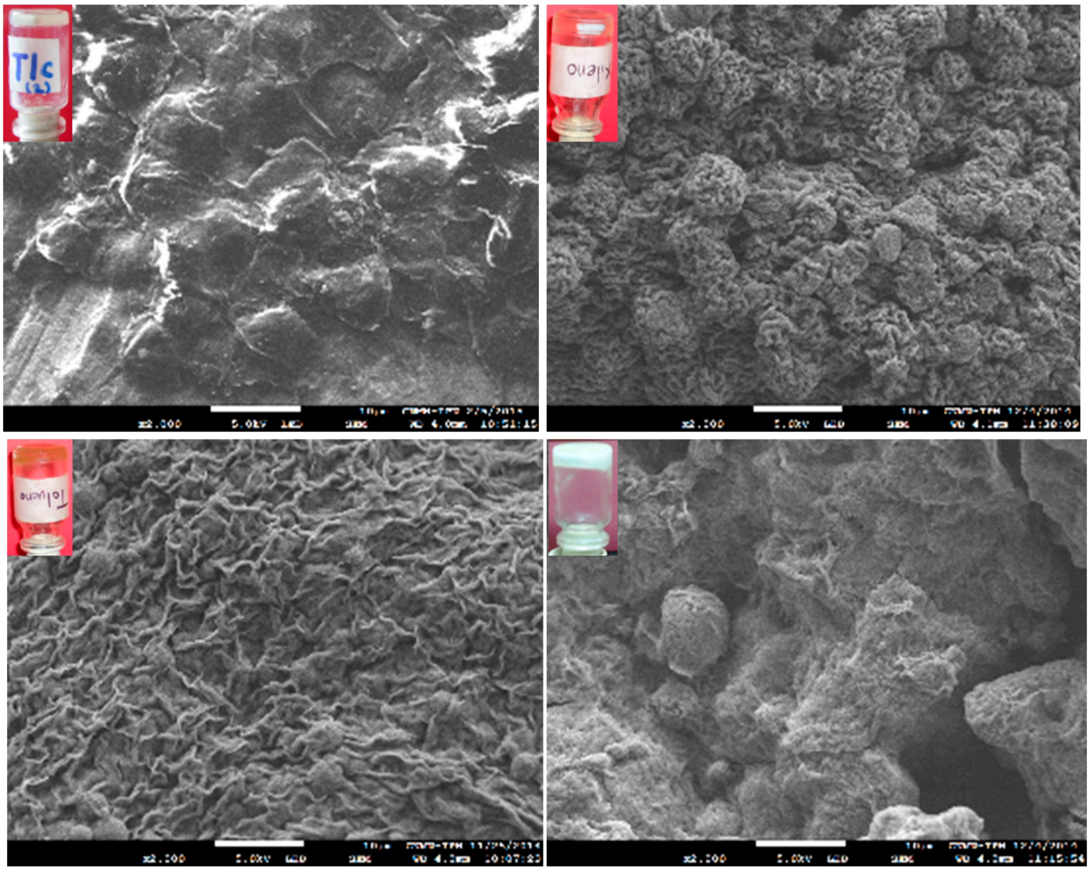

**5c** urea with carbon tetrachloride, xylene, toluene and 1,4-dioxane, respectively.

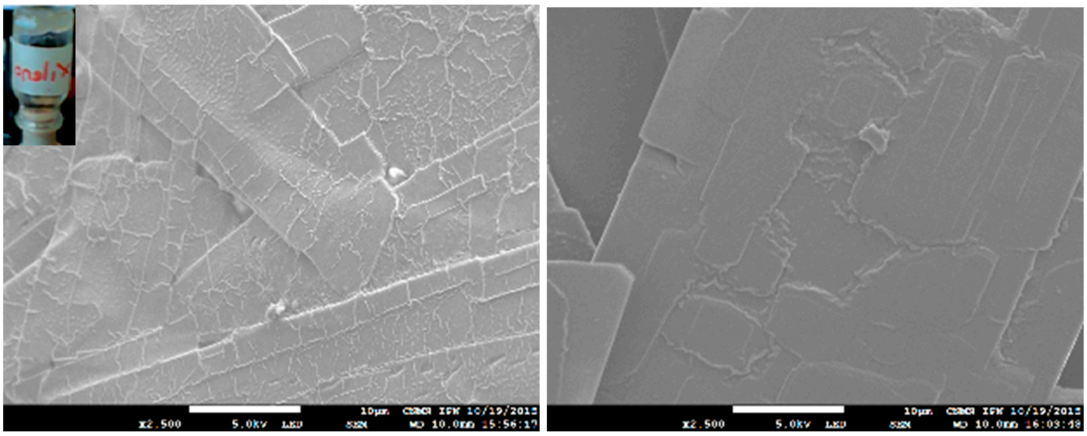

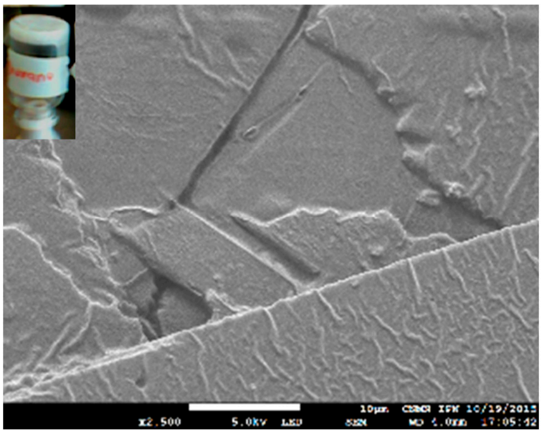

**5d** urea with xylene, toluene and 1,4-dioxane, respectively.

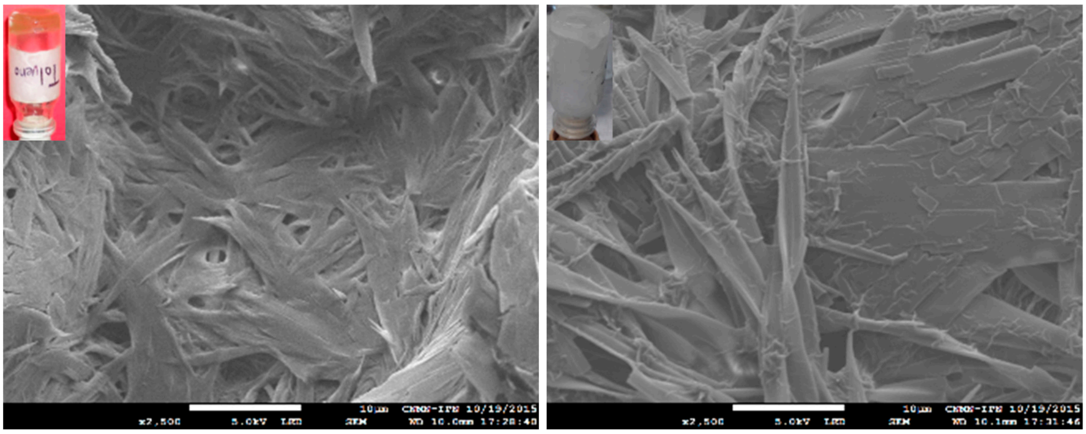

**5e** urea with toluene and 1,4-dioxane

Figure S49. Photographs of the flasks and scanning electron microscopy micrographs of the gels obtained by interacting **5a-5e** ureas with different organic solvents.

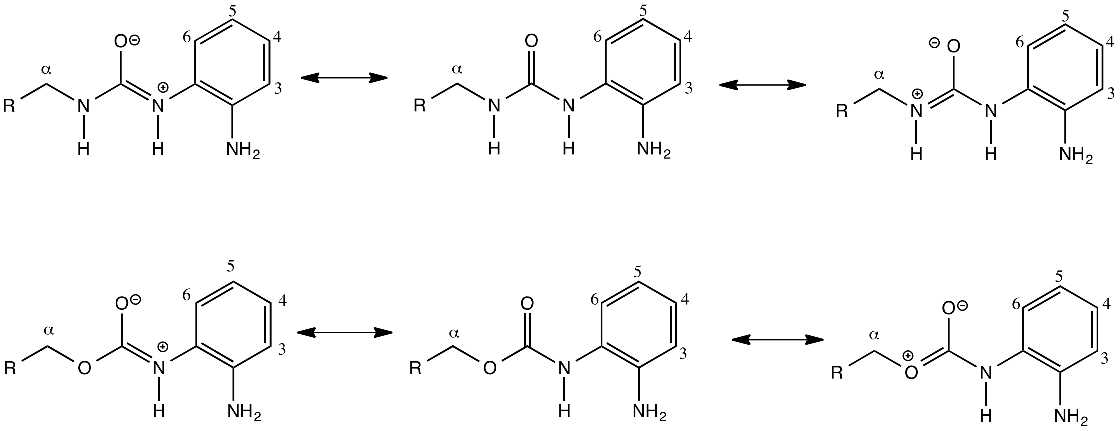

Scheme S3. Resonant effect on the nitrogen and oxygen atoms on the double bond of the carbonyl

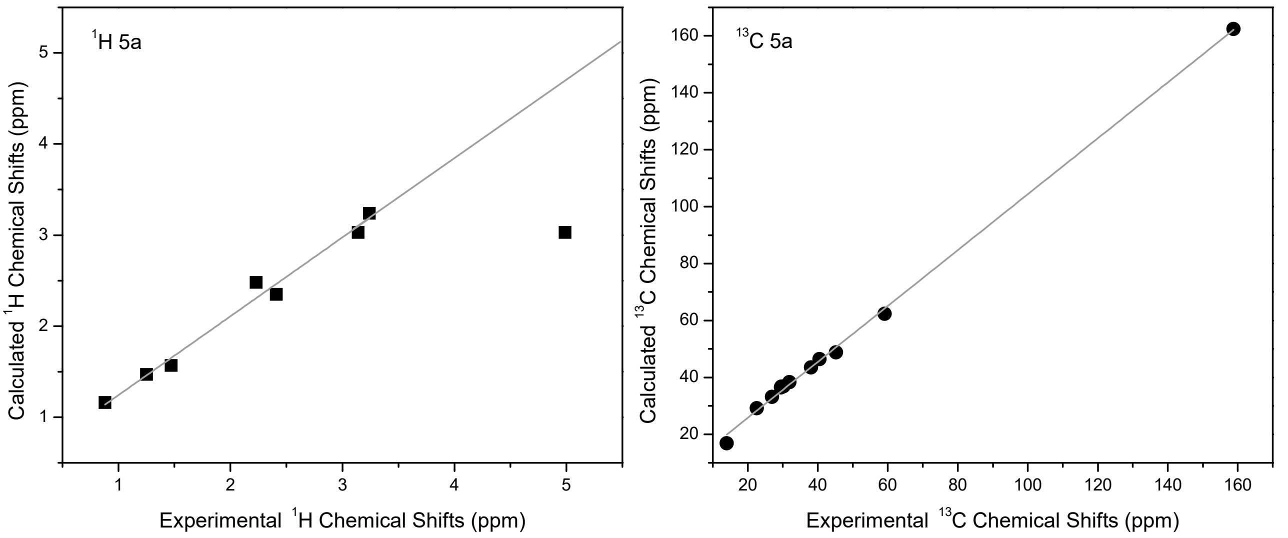


Figure S50. Linear correlation plots of a $^{13}C$ and $^{1}H$ chemical shifts values of organogel with **5a** urea.

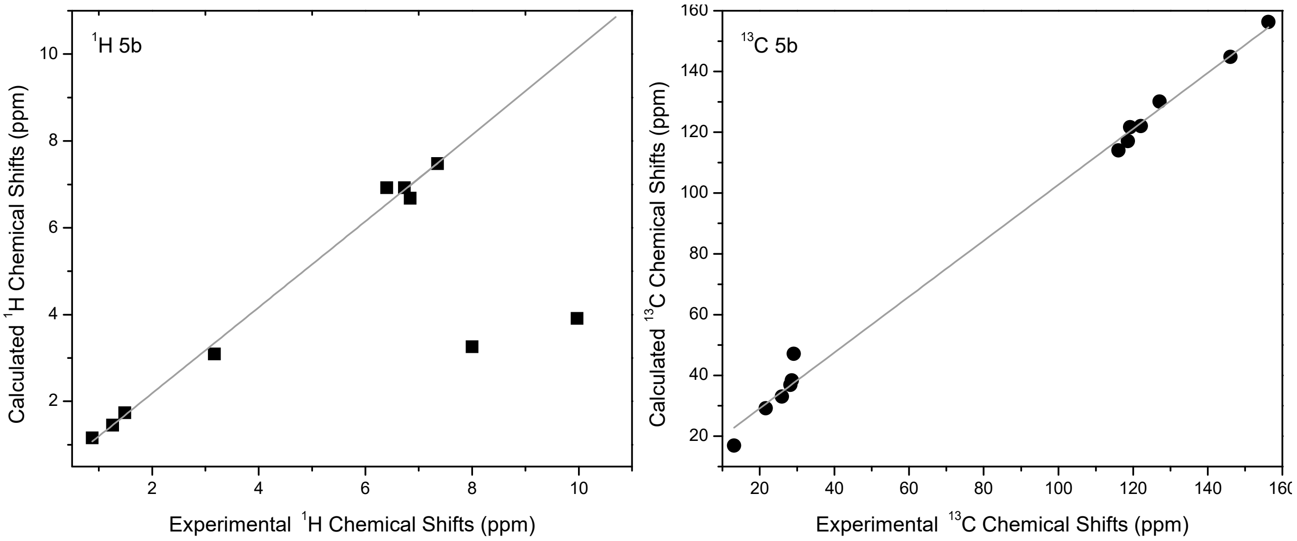


Figure S51. Linear correlation plots of a $^{13}C$ and $^{1}H$ chemical shifts values of organogel with **5b** urea.

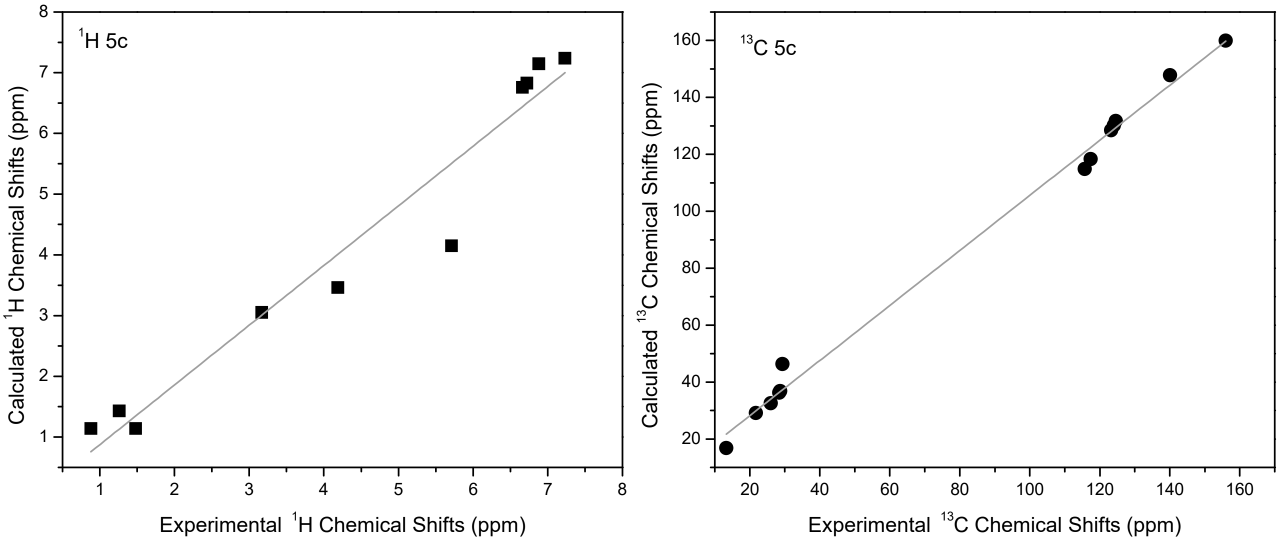


Figure S52. Linear correlation plots of a $^{13}C$ and $^{1}H$ chemical shifts values of organogel with **5c** urea.

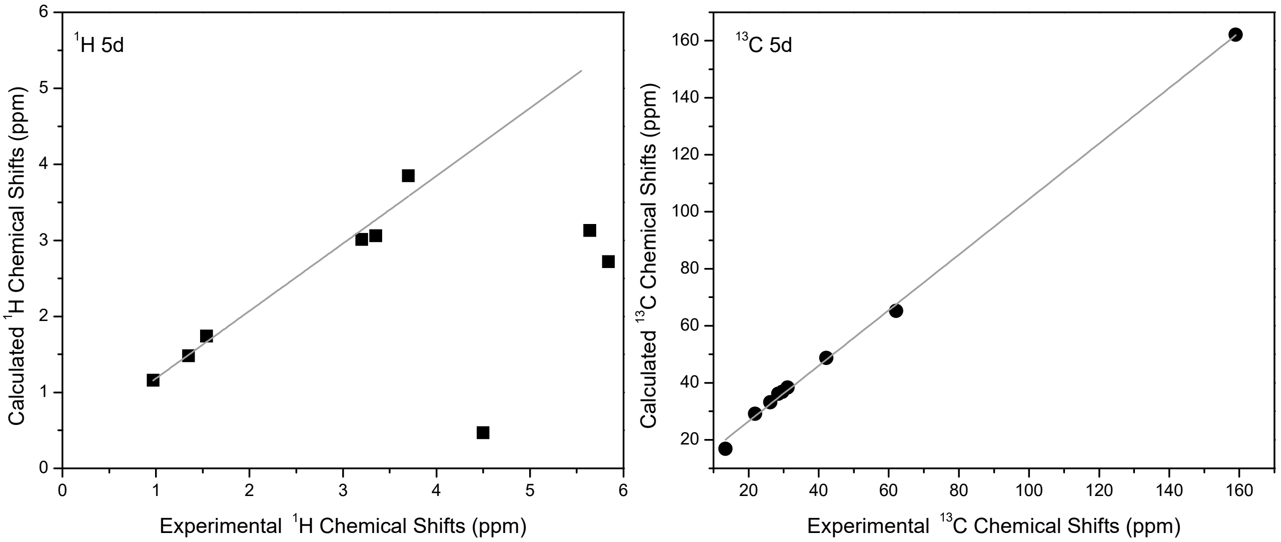


Figure S53. Linear correlation plots of a $^{13}C$ and $^{1}H$ chemical shifts values of organogel with **5d** urea.

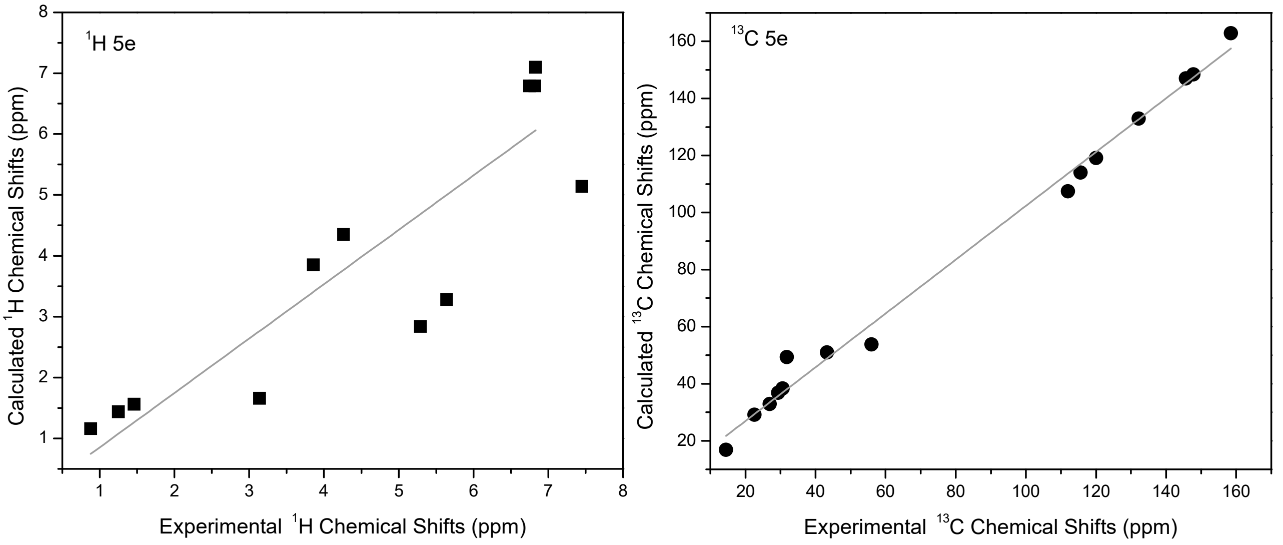


Figure S54. Linear correlation plots of a $^{13}$C and $^{1}$H chemical shifts values of organogel with **5e** urea.

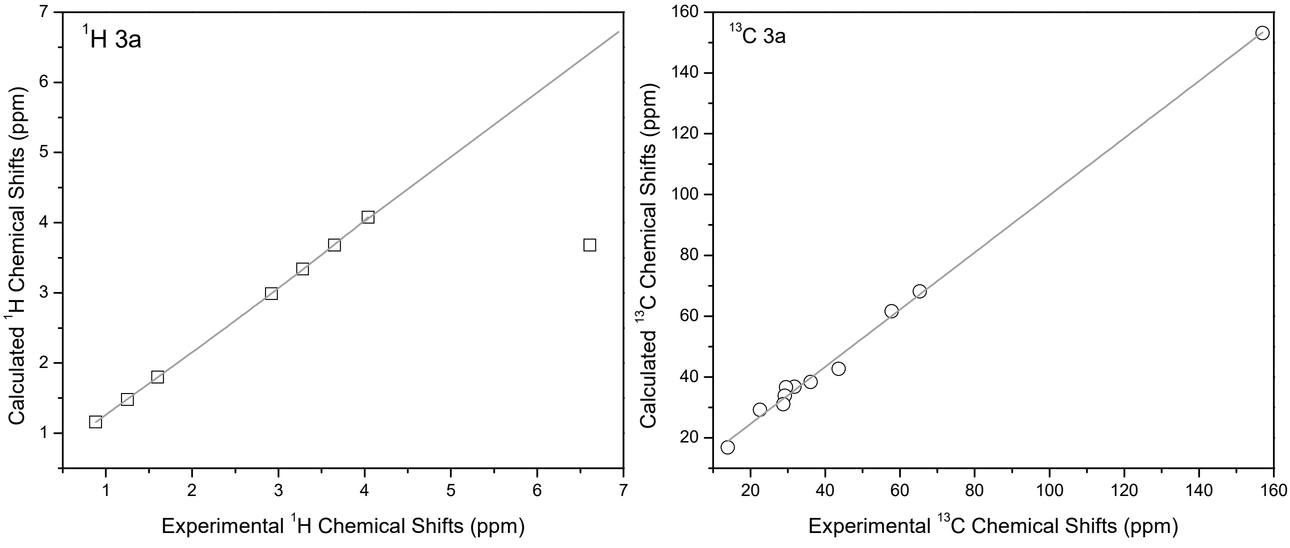


Figure S55. Linear correlation plots of a $^{13}$C and $^{1}$H chemical shifts values of organogel with **3a** carbamate.

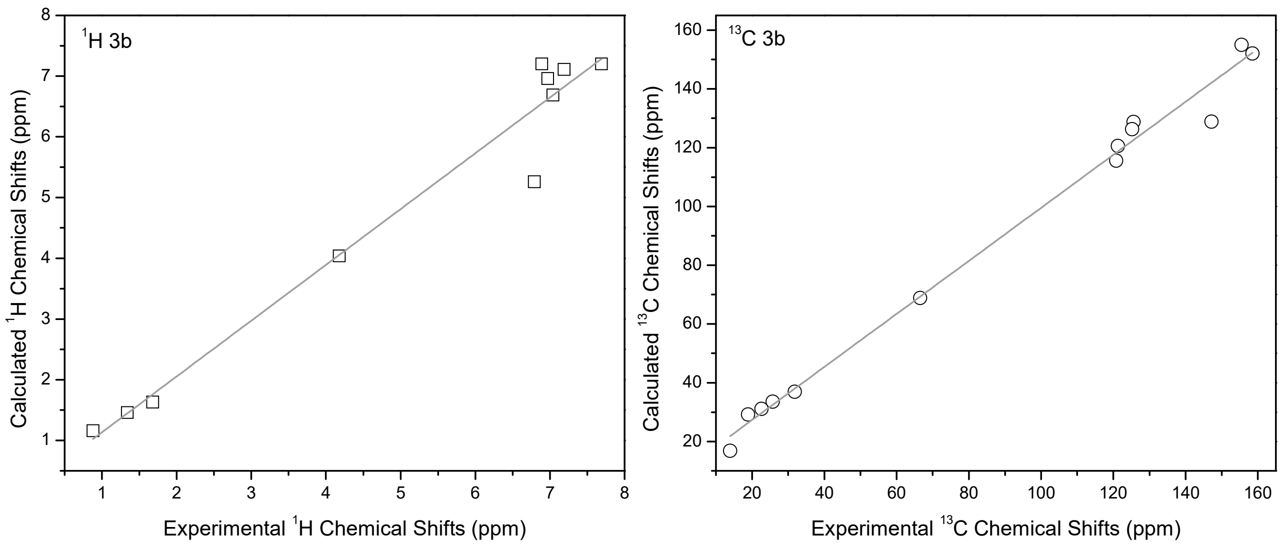


Figure S56. Linear correlation plots of a $^{13}$C and $^{1}$H chemical shifts values of organogel with **3b** carbamate.

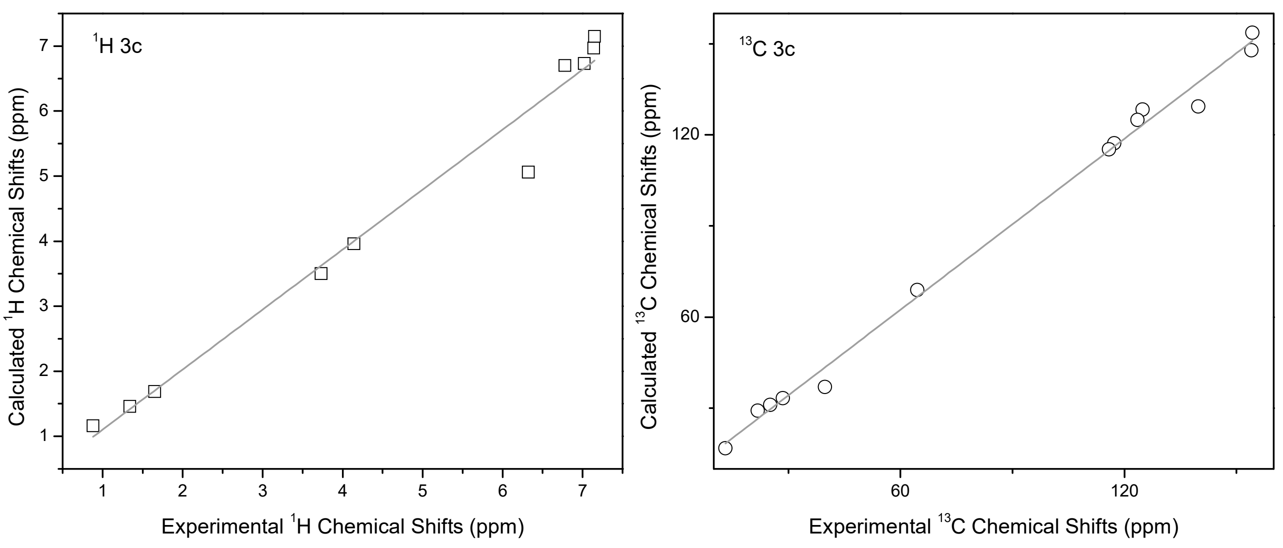


Figure S57. Linear correlation plots of a $^{13}$C and $^{1}$H chemical shifts values of organogel with **3c** carbamate.

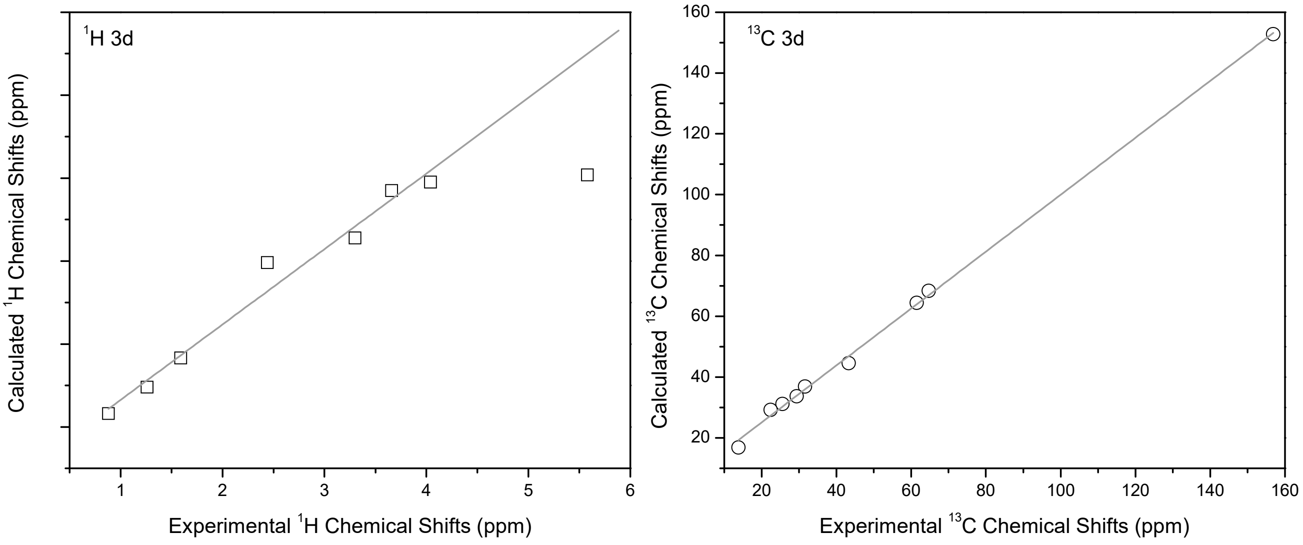


Figure S58. Linear correlation plots of a $^{13}C$ and $^{1}H$ chemical shifts values of organogel with **3d** carbamate.

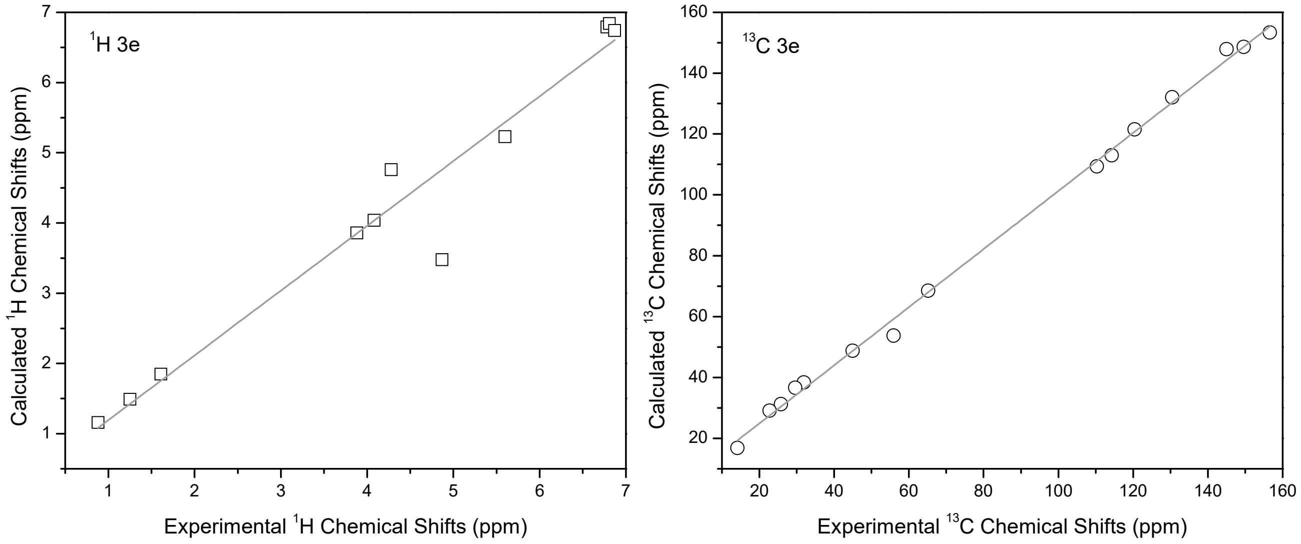


Figure S59. Linear correlation plots of a $^{13}C$ and $^{1}H$ chemical shifts values of organogel with **3e** carbamate.

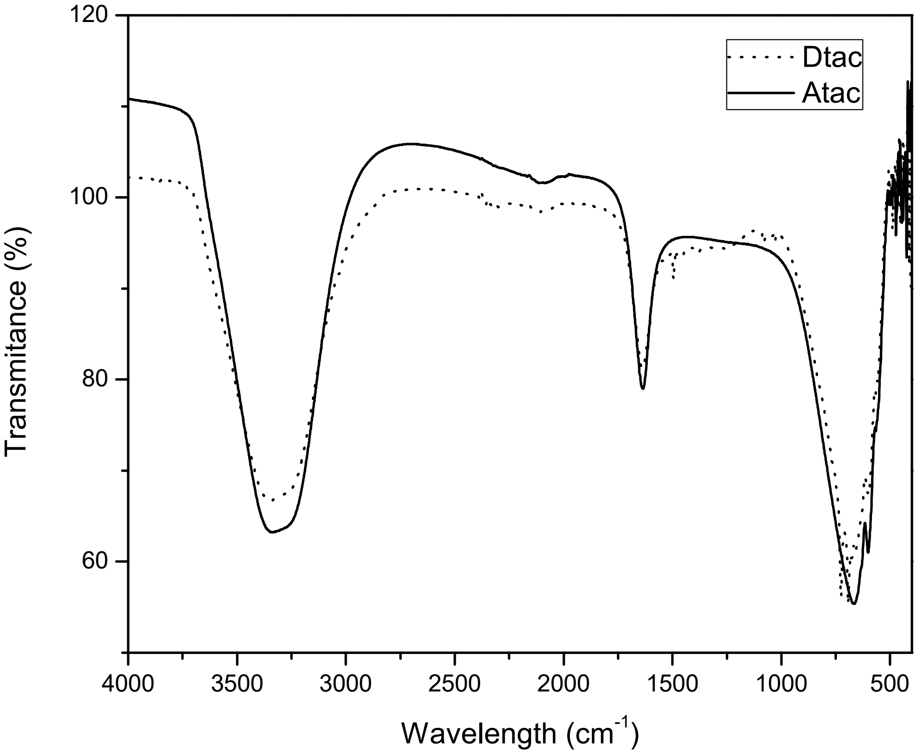


Figure S60. FT-IR spectrum of the traces of toluene in water before and after the treatment with **3d** carbamate.

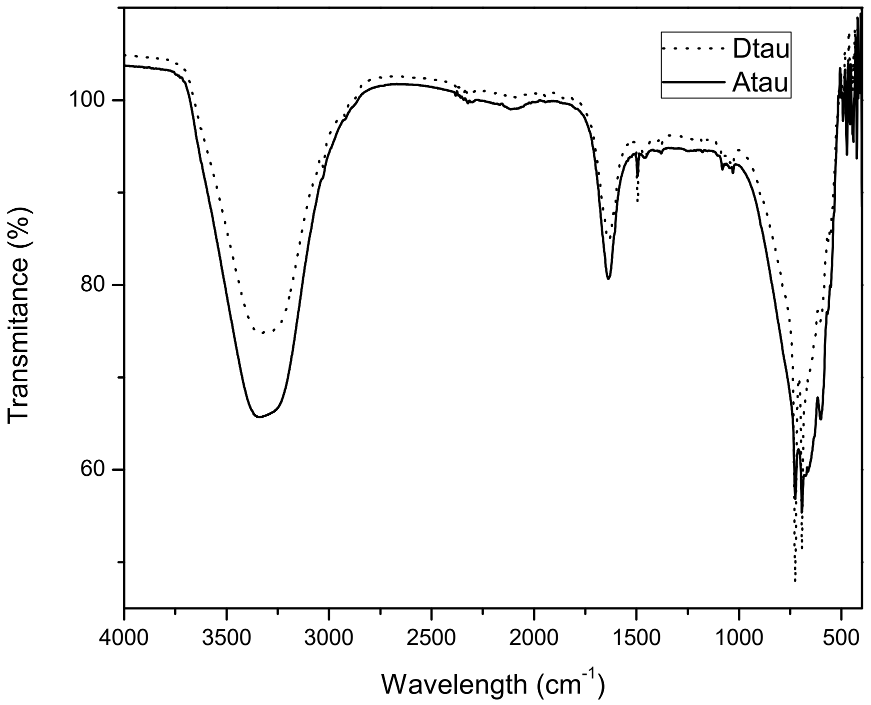


Figure S61. FT-IR spectrum of the traces of toluene in water before and after the treatment with **5d** urea.

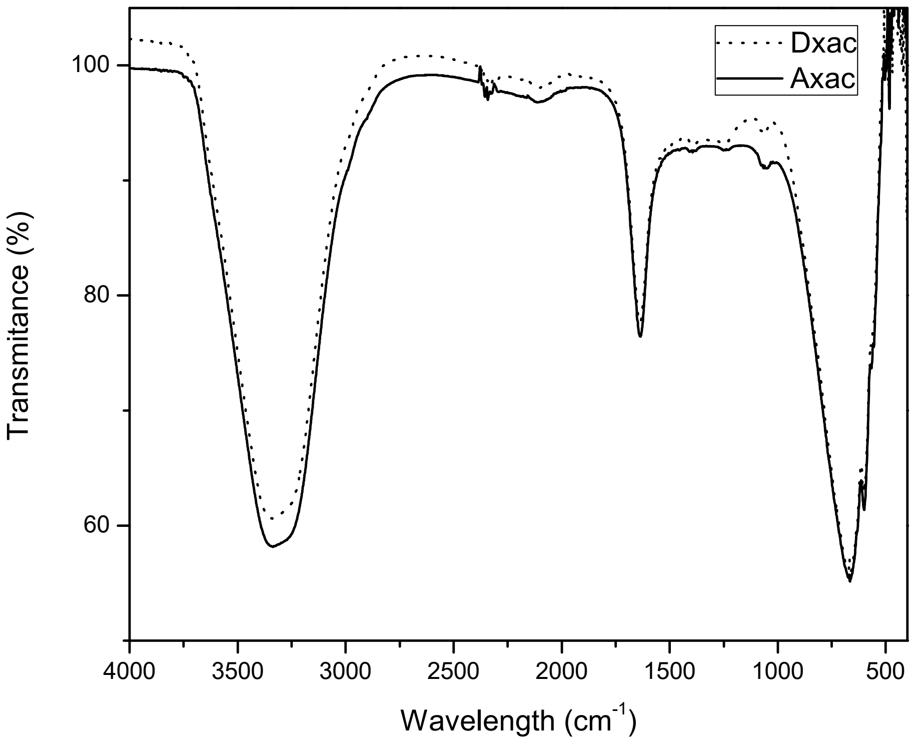


Figure S62. FT-IR spectrum of the traces of xylene in water before and after the treatment with **3d** carbamate.

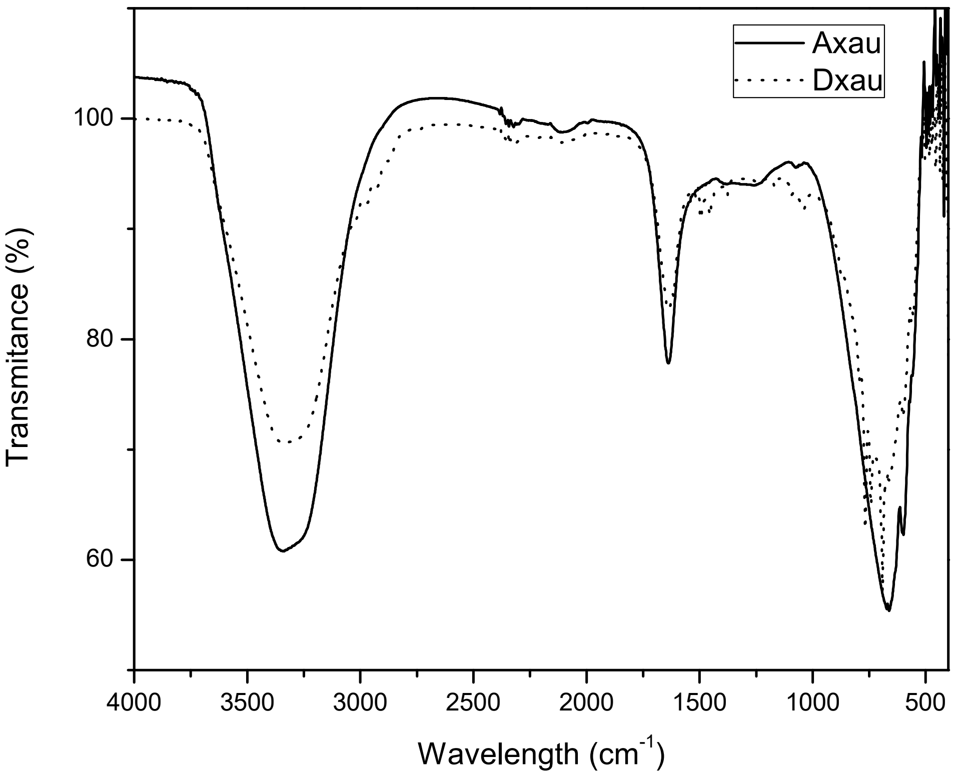


Figure S63. FT-IR spectrum of the traces of xylene in water before and after the treatment with **5d** ureas.

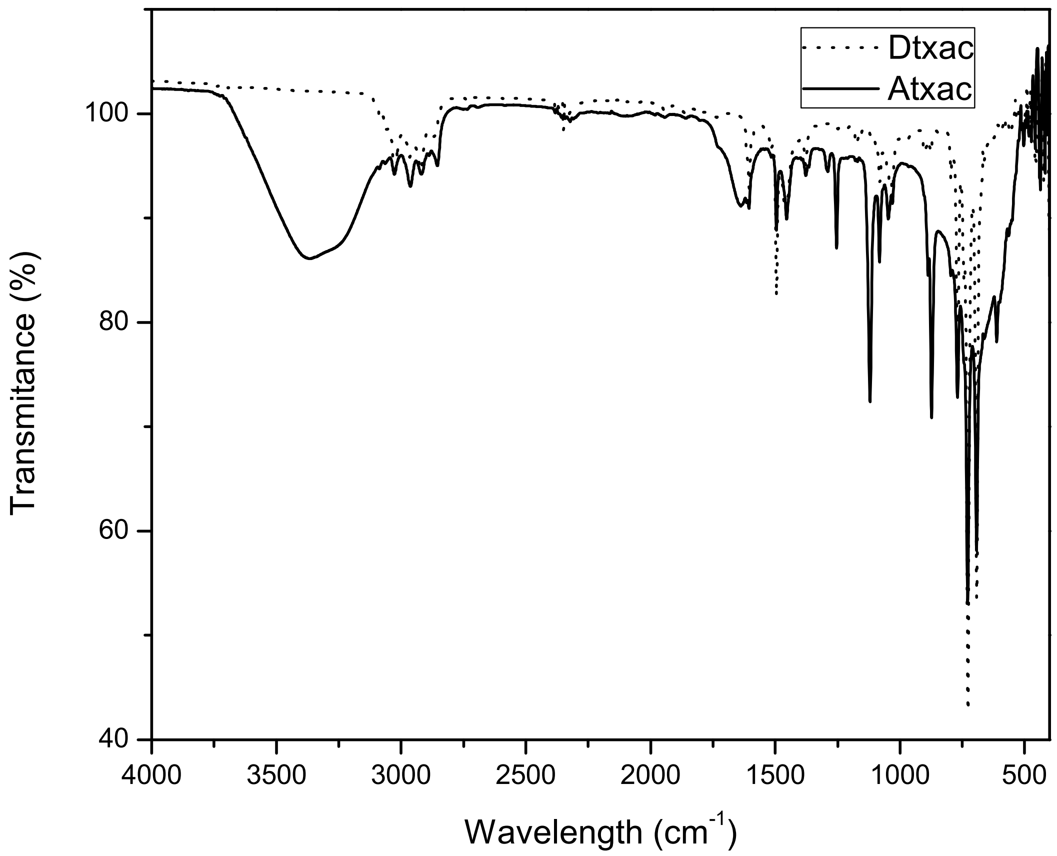


Figure S64. FT-IR spectrum of the traces of toluene- xylene in water before and after the treatment with **3d** carbamate.

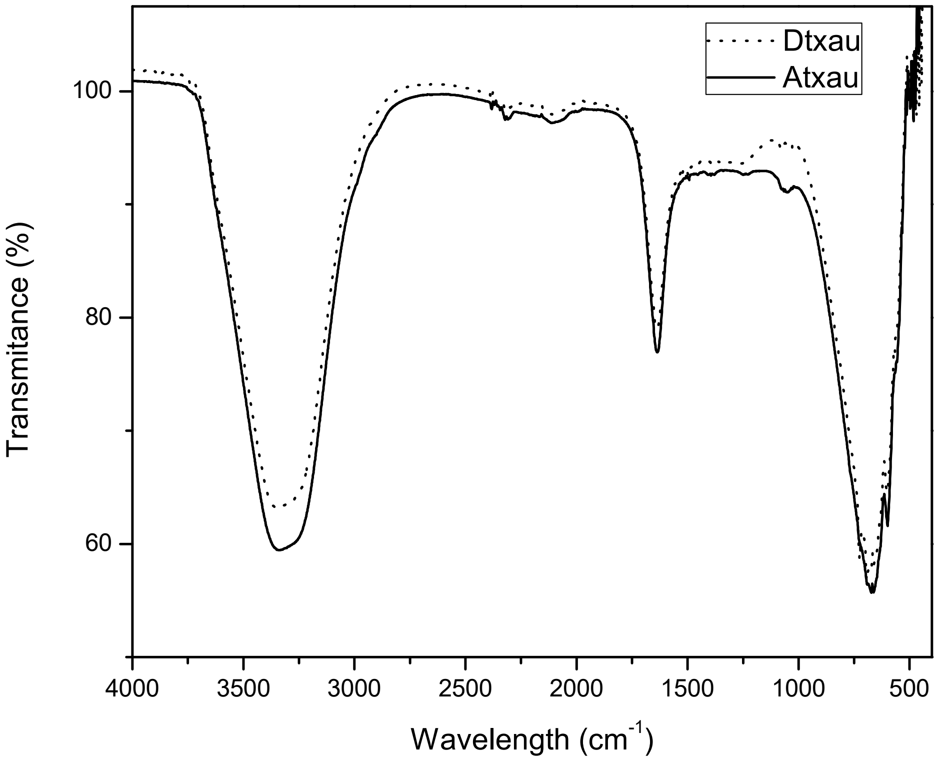


Figure S65. FT-IR spectrum of the traces of toluene- xylene in water before and after the treatment with **5d** urea.

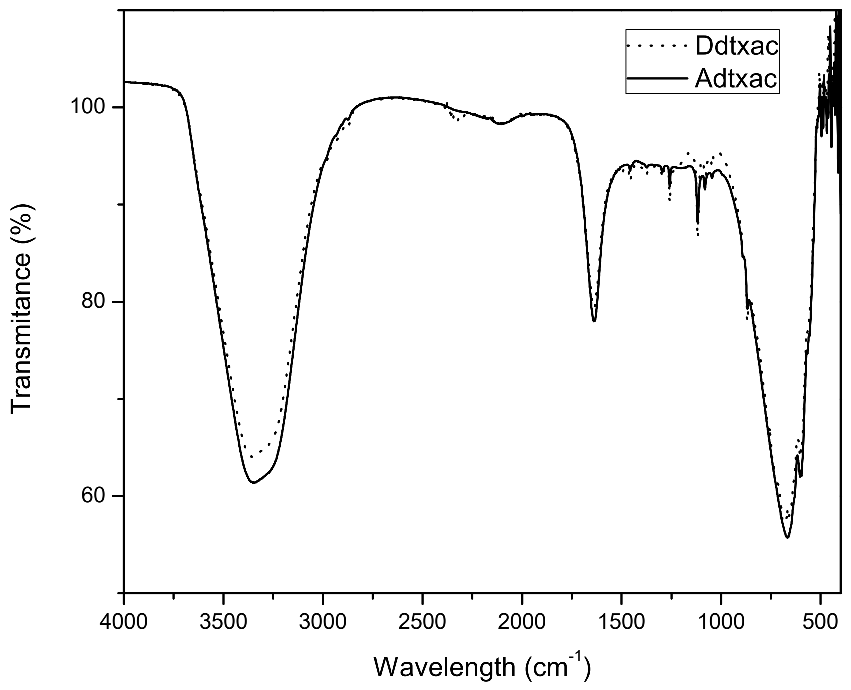


Figure S66. FT-IR spectrum of the traces of dioxane-toluene- xylene in water before and after the treatment with **3d** carbamate.

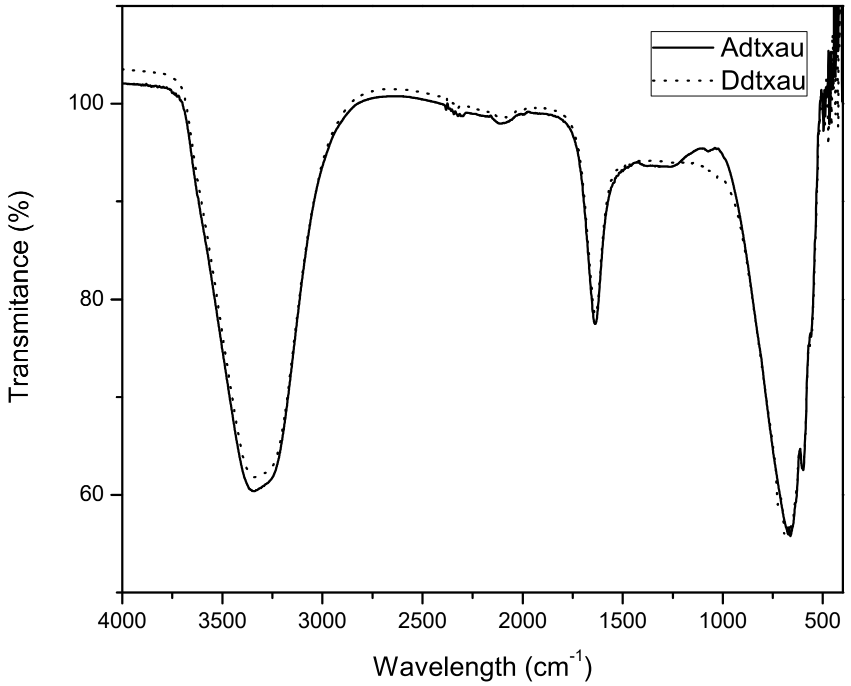


Figure S67. FT-IR spectrum of the traces of dioxane-toluene- xylene in water before and after the treatment with **5d** urea.

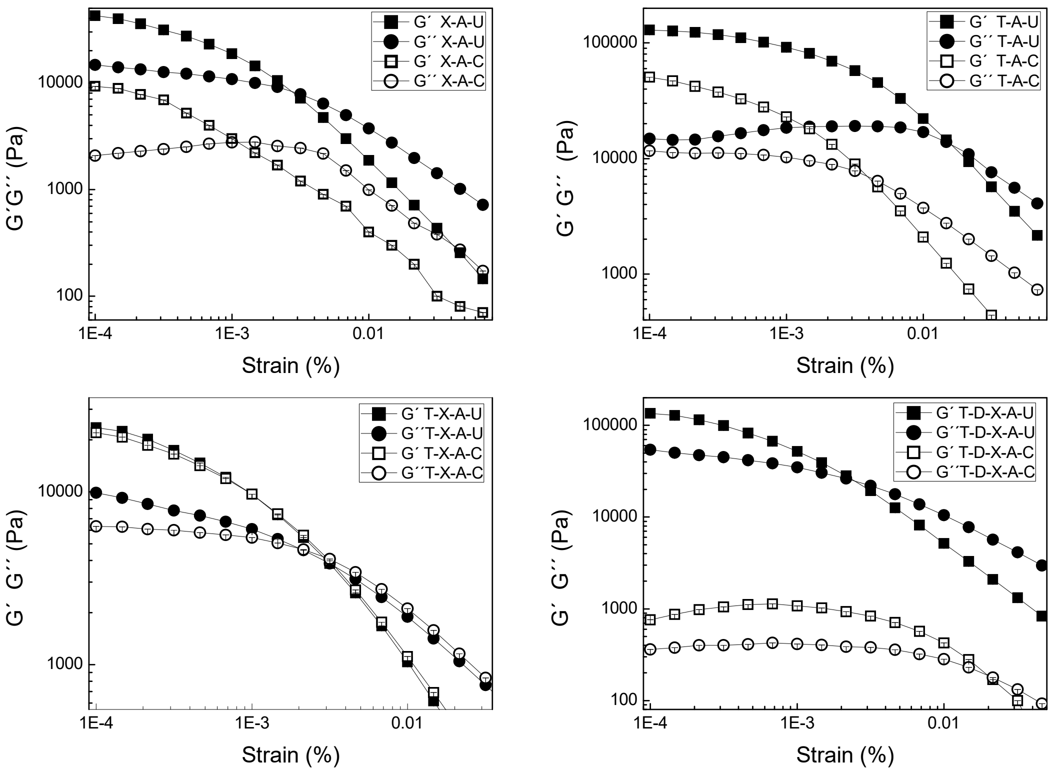


Figure S68. Storage modulus (G´) and loss modulus (G´´) as a function of strain for organogels of **5d** urea (G´-■) (G´´-●) and **3d** carbamate (G´-□) (G´´-○) with mixture of solvents with water. Urea (U) and carbamate (C). Xylene-water (X-W), toluene- water (T-W), toluene-xylene-water (T-X-W) and toluene-dioxane-xylene-water (T-D-X-W).

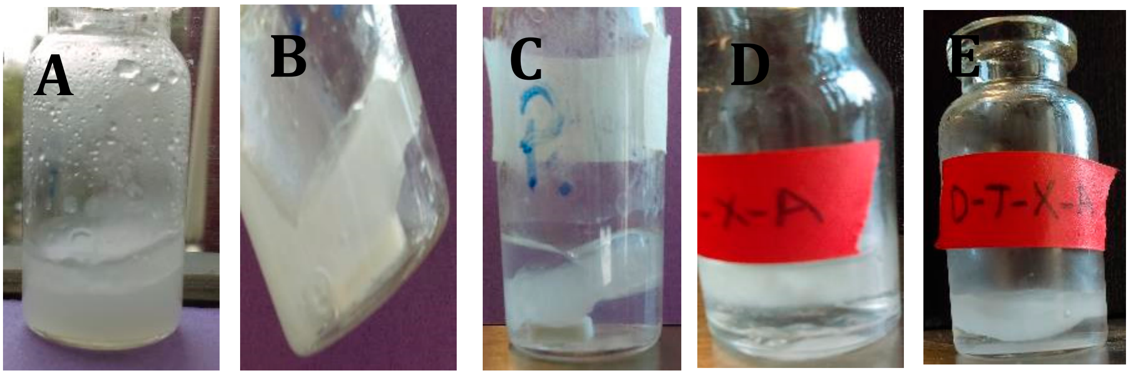


Figure S69. Photographs of removal organogels solvent of **3d** carbamate with mixture of solvents with water. (A). Dioxane-water, (B) toluene-water, (C) xylene-water, (D) toluene-xylene-water and (E) dioxane-toluene-xylene-water.

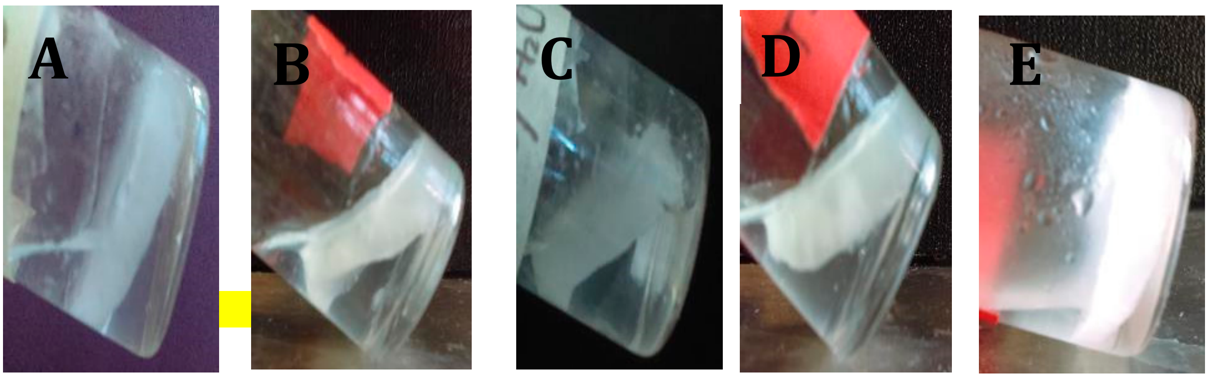


Figure S70. Photographs of removal organogels solvent of **5d** urea with mixture of solvents with water. (A). Dioxane-water, (B) toluene-water, (C) xylene-water, (D) toluene-xylene-water and (E) dioxane-toluene-xylene-water.
